# Structural dynamics of DNA mimic foldamers

**DOI:** 10.1039/d5sc08567e

**Published:** 2025-12-08

**Authors:** Manuel Loos, Lion Thurecht, Jiaojiao Wu, Valentina Corvaglia, Zhiwei Liu, Vojislava Pophristic, Martin Zacharias, Ivan Huc

**Affiliations:** a Department Pharmazie, Ludwig-Maximilians-Universität München Butenandtstr. 5-13 81377 München Germany ivan.huc@cup.lmu.de; b Physik-Department and Center of Protein Assemblies, Technische Universität München James Franck Str. 1 85748 Garching Germany martin.zacharias@mytum.de; c Department of Chemistry & Biochemistry, Rowan University 201 Mullica Hill Road Glassboro New Jersey 08028 USA

## Abstract

DNA mimic foldamers are helically folded aromatic oligoamides bearing negatively charged side chains that mimic the shape and charge distribution of double-stranded B-DNA. They have been shown to bind to some DNA-binding proteins better than DNA itself and thus have potential to interfere with DNA-protein interactions. Their structure has been previously characterized in detail by X-ray crystallography. We have now investigated their structural dynamics both computationally and experimentally. The force field parameters of the building blocks required for DNA mimicry were optimized and implemented in AMBER to perform molecular dynamics simulations of the foldamer helices. The position of the negatively charged side chains on the helix, the charge state of the side chains, and the presence of salt were systematically varied. The simulations revealed that the global flexibility parameters for twisting and bending of the foldamer helices are of similar magnitude to those of B-DNA, though distinct kinking events and motions are involved. A range of sequences were then prepared for experimental investigations using ^1^H NMR, UV-vis absorption and circular dichroism spectroscopies. Measurements revealed that the foldamer helices are stable over a broad range of temperature, pH and salt conditions in aqueous solutions, but that they nevertheless undergo structural changes when conditions are modified. An assay was developed to quantitatively assess foldamer helix stability through the measurement of the rate of interconversion between right-handed and left-handed diastereomeric conformers. Unexpectedly, suppressing some negatively charged side chains had a destabilizing effect on the helix, suggesting a more complex role of the side chains than electrostatic repulsions.

## Abstract 2

The structural dynamics of double stranded B-DNA and single helical foldamers that mimic DNA duplexes show differences that may play a role in the ability of the foldamers to outperform DNA in binding to some proteins.

## Graphical abstract

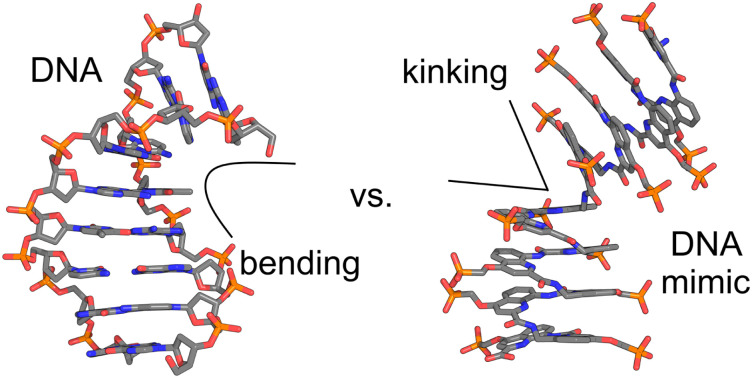

## Introduction

The molecular recognition properties of DNA include its ability to form duplex structures *via* A:T and G:C base pairs (bp) and the interactions of these duplexes with a large number of proteins required for the repair, packaging, regulation, transcription, and duplication of genetic information. Molecules that mimic the shape and surface features of DNA duplexes may engage interactions with these DNA-binding proteins, interfere with protein-nucleic acid interactions (PNIs), and serve as pharmacological, diagnostic, or even therapeutic tools. These mimics are to be distinguished from other analogues of DNA that reproduce its base pairing ability, such as peptide nucleic acids (PNAs) and locked nucleic acids (LNAs), whose target is DNA, not DNA-binding proteins.^1,2^ The most obvious analogues of DNA's overall shape are derived from DNA itself and called DNA decoys.^3–5^ In nature, some proteins that mimic DNA have been shown to interfere with PNIs.^6–8^ It is actually remarkable that the peptide backbone may generate shapes similar to a DNA duplex. Subsequently, coiled-coil peptides have been proposed to serve for that purpose,^9^ and some anionic polymers such as heparin are known to bind to DNA-binding proteins so much so that heparin-chromatography is used for their purification.^10^ Along this line, we have developed helically folded aromatic oligoamide foldamer-based DNA mimics that reproduce the overall shape and negative charge distribution of double-stranded B-DNA.^11–16^ These DNA mimic foldamers are chemically remote from DNA – they contain neither sugar nor nucleobase – but they are able to bind to some DNA binding proteins better than DNA itself and to inhibit PNIs.^11,12^ The structural resemblance between DNA and DNA mimic foldamers is essential to explain why the latter bind to DNA-binding proteins. Conversely, only structural differences between DNA and DNA mimic foldamers may explain why the mimics may outcompete DNA and inhibit PNIs. We have reported several solid-state structures of DNA mimic foldamers either alone or bound to a chromosomal protein,^11,12,14,17^ shedding some light on their structural features and gathering useful information to understand and further improve DNA-mimic foldamer inhibition of PNIs. However, information about their structural dynamics and how they compare with those of DNA is lacking. Here, we report a computational and experimental investigation of the structural dynamics of DNA mimic foldamers. We analyze parameters such as overall stability, shape, kinking, and bending properties, as a function of the charge state (pH), the presence of salt, and temperature and highlight both resemblances and differences between DNA mimic foldamers and DNA.

**Fig. 1:**
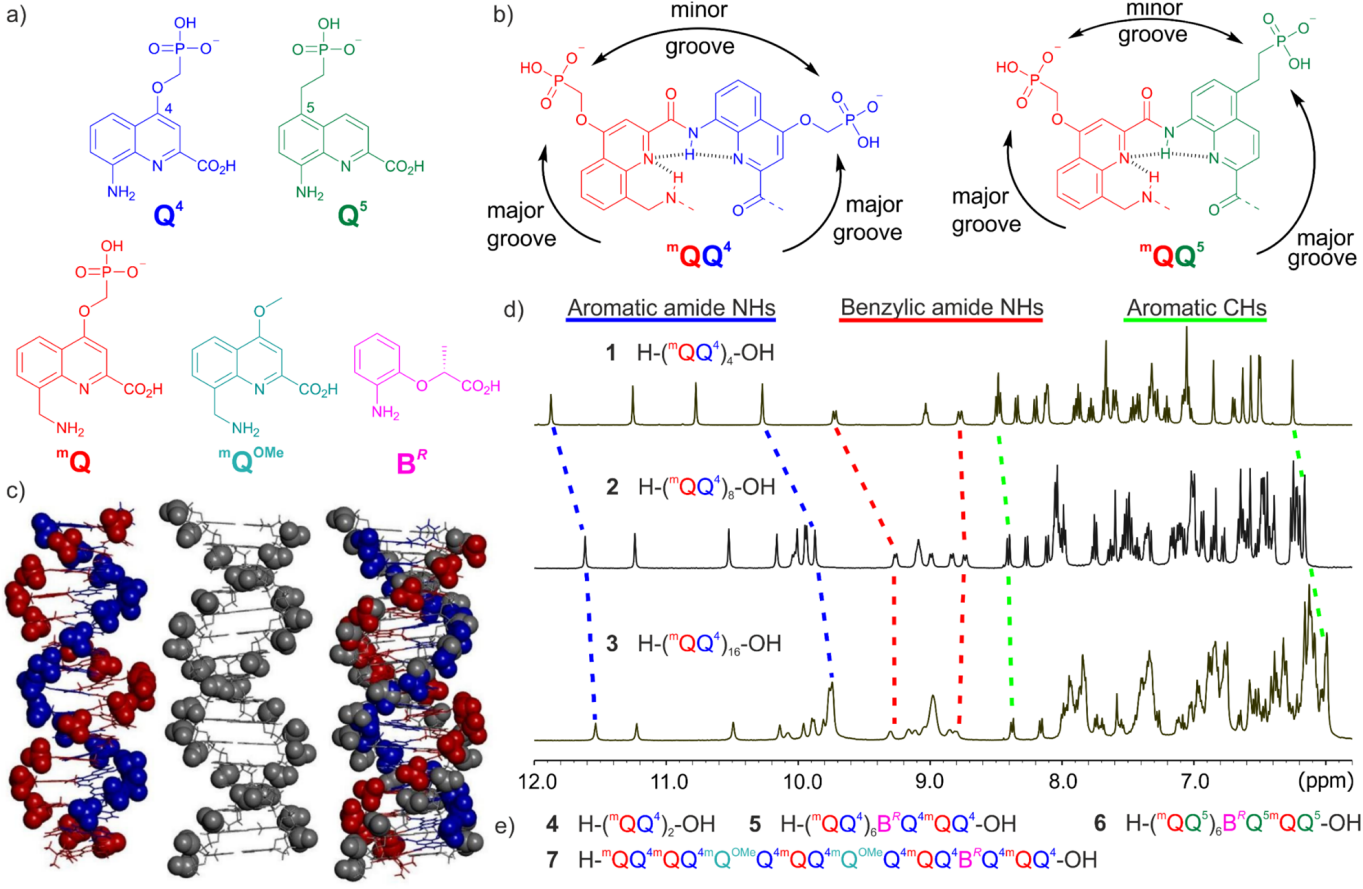
(a) Structural formulae of amino acid monomers Q^4^, Q^5^, ^m^Q, ^m^Q^OMe^, and B^*R*^. (b) Formulae of ^m^QQ^4^ and ^m^QQ^5^ repeat units. (c) Left: solid-state structure of the side chain-protected (^m^QQ^4^)_16_ (^m^Q units in red, Q^4^ units in blue, stick representation except the phosphonate groups shown in space filling representation, ethyl ester protection of the phosphonate omitted for clarity),^11^ center: structure of the 16-bp B-DNA duplex d(ACTGAACGGCTACGTA)·d(TGACTTGCCGATCAT) (gray, stick representation except the phosphonate groups shown in space filling representation), right: overlay of the two structures. (d) Part of the ^1^H NMR spectra of (^m^QQ^4^)_4_ (1), (^m^QQ^4^)_8_ (2), and (^m^QQ^4^)_16_ (3) in 50 mM ammonium bicarbonate in H_2_O/D_2_O (9 : 1 v/v) at 25 °C showing amide NH and aromatic CH resonances. (e) Foldamer sequences 4–7 used to investigate the kinetics of helix handedness reversal.

The original DNA mimic foldamer design consists of single-stranded oligoamides in which two types of amino acid monomers alternate: 8-amino-2-quinolinecarboxylic acid Q and 8-aminomethyl-2-quinolinecarboxylic acid ^m^Q (Fig. 1a). Q carries an aromatic amine whereas ^m^Q carries an aliphatic (benzylic) amine. Both types of monomers may be functionalized with a phosphonic acid-containing side chain that can be mono or dianionic in aqueous solution depending on pH, in contrast with phosphodiesters in DNA which cannot be dianionic. Q^4^ and Q^5^ have their side chain in position 4 and 5 of the quinoline ring, respectively, and also differ by an oxygen *vs.* a carbon atom as the first exocyclic atom of the side chain (Fig. 1a). Q_*n*_ oligomers are well known to adopt stable single-helical conformations both in the solid state and in solution,^18–21^ and so do (^m^QQ)_*n*_ DNA mimic oligomers. In both cases, helical structures are stabilized by hydrogen bonds between consecutive units in the sequence (Fig. 1b) and, in protic solvents, by hydrophobic effects associated with aromatic stacking. However, a unique feature of the (^m^QQ)_*n*_ single helix is that its curvature is such that side chains borne by ^m^Q and Q units form a double helical array of *exo*-helices^22^ and that their positions match the positions of phosphate groups in B-DNA (Fig. 1c). Thus, the single helix of (^m^QQ)_*n*_ features two grooves that resemble the minor and major grooves of B-DNA. The grooves are defined as shown in Fig. 1b which also highlights that the major groove is wider and the minor groove narrower in (^m^QQ^5^)_*n*_ than in (^m^QQ^4^)_*n*_. Solid state structures of (^m^QQ)_*n*_ single helices largely explained their resemblance with the shape of the B-DNA double helix.^11,12,14^ Each ^m^QQ dimer is similar in size to a DNA base pair, defining a foldamer helix diameter comparable to the diameter of B-DNA, and contributes about 0.9 helix turn, meaning consecutive ^m^QQ dimers are twisted by *ca.* 0.1 turn (36°), as are base pairs in B-DNA. Furthermore, the helix pitch in a foldamer helix, *i.e.,* the vertical rise per turn, equals the thickness of an aromatic ring (*ca*. 3.5 Å), like the base pair distance in B-DNA. In solution, the good solubility (absence of aggregation even at mM concentrations) and folding of (^m^QQ)_*n*_ give rise to sharp ^1^H nuclear magnetic resonance (NMR) spectra in which signals are spread over a wide range of chemical shift values despite the repetitive nature of the sequence (Fig. 1d).^21^ This spreading results from the upfield shifts of NMR signals due to ring current effects between intramolecularly stacked aromatic rings. The upfield shifts become more pronounced as the helix length increases (Fig. 1d), indicating a cumulative effect. Because ring current effects quickly decay with distance,^23,24^ cumulative effects are possible only if the helix is conformationally very well defined.^21^

As shown in the following, computations and experiments concur to support that ^m^QQ^4^ and ^m^QQ^5^ oligomers adopt stable helical conformations over a wide range of temperature and pH. Their global flexibility parameters for twisting and bending were found to be of similar magnitude as those of B-DNA. However, their structural dynamics reflect the properties of the aromatic amide backbone and differ from those of DNA. For example, reversible unstacking events occur within the aromatic helix, leading to temporary kinks with transient redirection of the helical axis. Some of these events can last longer than 100 ns. We report that pH, *i.e.* the charge state of the phosphonate side chains appears to have the greatest effect on these structural dynamics, but also that the effect of the charged phosphonate side chains is more complex than simple helix-destabilizing electrostatic repulsions. In case of singly-anionic phosphonates, hydrogen bonding between neighboring phosphonates may also stabilize close contacts. Eventually, removing a charged side chain was found to have a stabilizing effect on the helix. Finally, experiments and simulations revealed that helices in the ^m^QQ^5^ series are more stable than those in the ^m^QQ^4^ series.

## Computational methods

### Construction of arylamide residues for computations

**Fig. 2:**
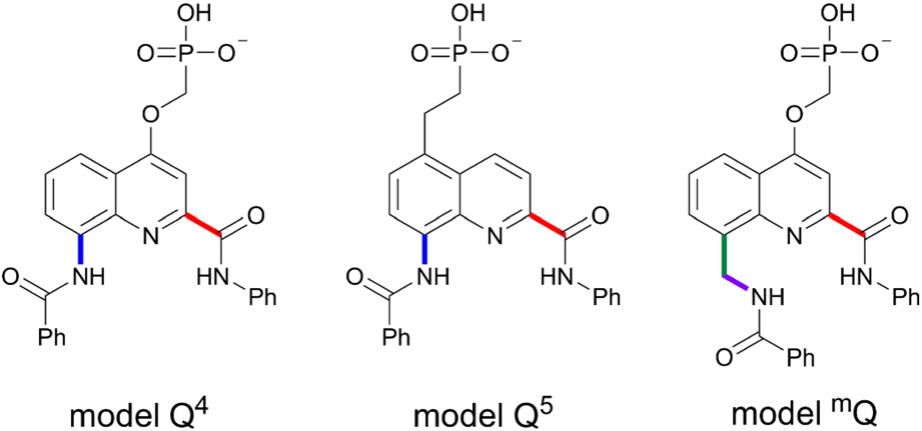
Model compounds used for RESP charge fitting. The terminal Ph–CO– and Ph–NH– groups have a sum of charges set to 0 and are removed to build the arylamide residues. Depicted in this figure are residues with a −1 charged side chain. Residues with a −2 charged side chain (further deprotonated) were also created following the same procedure. Bold bonds in color indicate re-parameterized torsions.

The arylamide residues were constructed using model compounds shown in Fig. 2 and following a protocol similar to the creation of amino acid residues in protein simulations. The partial atomic charges were derived using the multi-conformational fitting method provided by the R.E.D. tools^25^ for restrained electrostatic potential (RESP) charge derivation.^26^

### Optimization of torsional parameters

Several backbone torsional parameters were reparametrized by taking into consideration monomer specific effects such as the intramolecular hydrogen bonds between the quinoline endocyclic N and amide NH groups (Fig. 2, bonds shown in color). Specifically, the torsional parameters for the rotations about the aryl-amide bonds (blue and red bonds in Fig. 2), and about the aryl-aliphatic C (green) and aliphatic C-amide N (purple) bonds were re-parameterized using methods developed previously.^27,28^ Briefly, torsional energy profiles were calculated by a high level quantum mechanical (QM) method on model compounds bearing the same backbone functional groups. Next, non-bonded energies were evaluated using the general AMBER force field (GAFF)^29^ and RESP charges and subtracted from the QM torsional profile. Last, least square fitting was applied to fit the GAFF torsional term 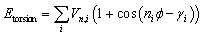 against the torsional profile with non-bonded corrections to obtain optimized parameters *V*_*n*,*i*_.

### Molecular dynamics simulations

For Molecular Dynamics (MD) simulations, we used (^m^QQ^4^)_18_ and (^m^QQ^5^)_18_ sequences consisting of thirty-six ^m^Q and Q^4^ or Q^5^ residues. Initial coordinates were generated by energy minimization to yield a starting straight helix geometry (without any kinking or bending) with a helical pitch of 3.5 Å. In the energy minimized structure, the pitch of the *exo*-helices spans ∼12 ^m^QQ units (∼24 residues), instead of ∼10 ^m^QQ in crystal structures. Sequence length was chosen to span more than one full *exo*-helix turn and, at the same time, kept short enough to limit the size of the simulated system in order to perform simulations in the microsecond regime. All phosphonate groups within a chain were either in a singly- or doubly anionic state. In solution, the second p*K*_a_ of an isolated phosphonic acid is expected to be near 6.5 but we have measured that the second p*K*_a_ of (^m^QQ^4^)_*n*_ oligomers is shifted to higher values centered above 8.5,^11^ probably due to the negative charge density of the helix. We performed MD simulations with both possible protonation states resulting in a total of four simulation systems. All simulations were performed with the Amber18 package.^30^ The systems were solvated with TIP3P water^31^ in an octahedral box with a minimum of 12 Å (for monoanionic phosphonates) and 25 Å (for dianionic phosphonates) between the DNA mimic foldamers and the box boundaries. A larger box size was chosen for the double charged system because we noticed a possible stretching of the molecule during test simulations. The ion concentration was adjusted to ∼0.1 M with added Na^+^ and Cl^−^ ions (neutral system) for most simulations. After energy minimization (2500 steps) using the sander module of the Amber18 package, the systems were heated in steps of 100 °C (each 0.1 ns) up to a temperature of 26.85 °C (300 K), keeping positional restraints on all non-hydrogen atoms with respect to the start structure. The positional restraints were removed within another 2 ns equilibration at 26.85 °C and constant pressure of 1 bar. Data gathering production simulations were extended to up to 2–3 µs for each system. All MD-simulations were performed using the pmemd.cuda program of Amber18 in combination with hydrogen mass repartition^32^ allowing a time step of 4 fs. Trajectory analysis for recording root-mean-square deviation (RMSD) *vs.* time, root-mean-square-fluctuations relative to the mean (RMSF) and analysis of dihedral angles was performed using the cpptraj module of Amber18. For the analysis of bending, twisting and stretching of the DNA mimics in-house scripts were used based on coarse graining of the chain. The averages over heavy atoms in two consecutive units were used to define the coarse-grained centers that follow the helical axis of the systems. The distance between these centers defines the helical rise (pitch) along the helical axis. The twist was calculated as the dihedral angle between the segments linking the helical axis and the geometric centers of aromatic rings. It can also be used to calculate the bending angle along the chain at each chain unit. The bending persistence length was calculated from the mean scalar product of the unit vector along the helical axis at the beginning of the chain and the axis vector at the end of the chain. Together with the geometric centers for each of the aromatic ring segments of each residue it is possible to define a rotation angle per unit along the chain and to calculate an overall periodicity or twist of the DNA mimics.

## Computational study

### General outcome of MD simulations

**Fig. 3:**
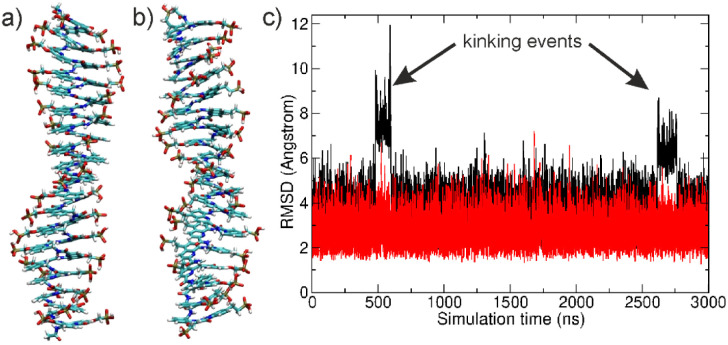
(a) Equilibrated start structure of the (^m^QQ^4^)_18_ DNA-mimic (36 residues = 18 repeat units) in stick representation. (b) Same as (a) for the (^m^QQ^5^)_18_ DNA-mimic. (c) RMSD of non-hydrogen atoms from the start structure *vs.* simulation time for the ^m^QQ^4^ (black) and ^m^QQ^5^ (red) DNA-mimics.

**Fig. 4:**
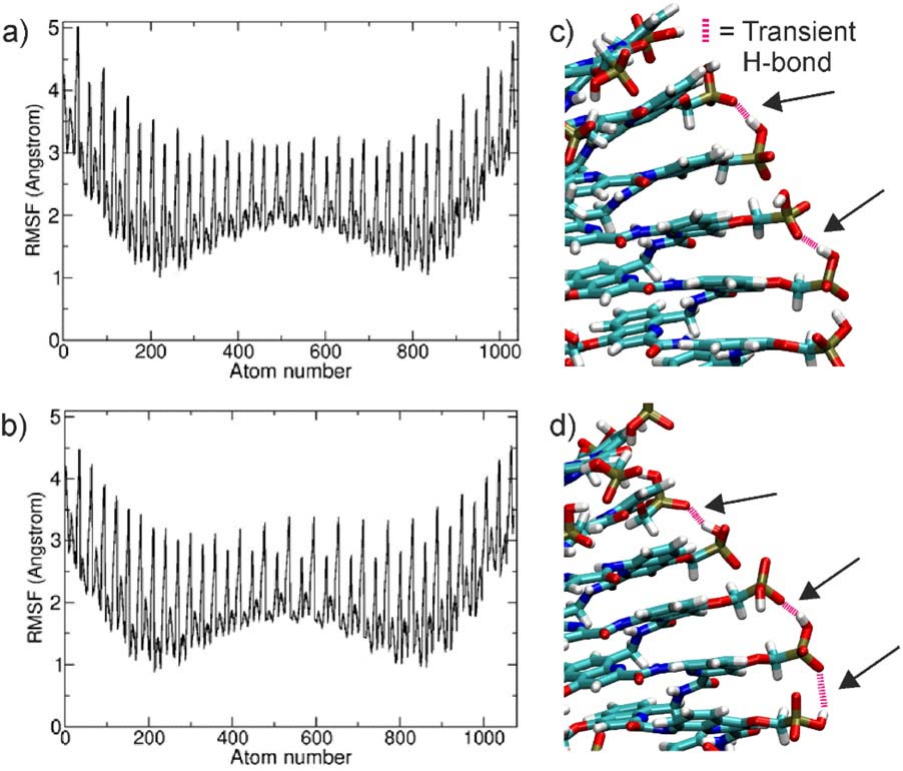
(a) Root-mean-square fluctuations (RMSF) of non-hydrogen atoms *vs.* atom number observed during (^m^QQ^4^)_18_ simulations (excluding the simulation parts with significant kinking). (b) Same as (a) for simulations on (^m^QQ^5^)_18_. The maxima in the plots indicate fluctuations of the phosphonate groups and the less fluctuating regions correspond to the main chain aromatic amide atoms. (c and d) Transient hydrogen bond formation (pink dashed lines and black arrows) from two snapshots of a segment of the (^m^QQ^4^)_18_ structure.

The simulations of (^m^QQ^4^)_18_ and (^m^QQ^5^)_18_ (also named ^m^QQ^4^ and ^m^QQ^5^ oligomers in the following paragraphs) with singly charged phosphonates resulted in rapid equilibration to conformations that remained within an RMSD of ∼3–4 Å relative to the start structure (Fig. 3). In the case of ^m^QQ^4^, two transitions to conformations with larger RMSD of ∼6 Å were observed that will be discussed in a separate paragraph below (Fig. 3c). The mean equilibrium twist as defined in the Methods section was larger for ^m^QQ^5^ than for ^m^QQ^4^ (28.8° *vs.* 25.5°, Table 1) and also indicated some unwinding relative to the experimental solid-state structure where the twist angle is closer to 36°,^11^ as between base pairs in B-DNA. We are not aware of such unwinding in simulations of B-DNA. In the foldamers, it results in a mean *exo*-helical periodicity of the structures of 14 units for ^m^QQ^4^ and 12.5 for ^m^QQ^5^ instead of 10 in the solid-state structures. A possible explanation for the unwinding is the frequent formation of hydrogen bonds between neighboring phosphonate groups that may influence the mean twist (Fig. 4c and d). On average 10–60% of the phosphonate groups were involved in hydrogen bonding (with *d*_H–O_ < 2.5 Å). Such stabilization of close contacts between phosphonate groups is not possible in the crystal structures where phosphonates were protected as ethyl esters. The mean helical pitch of 3.65 Å observed during the simulations is close to the pitch found in the crystal structure (3.5 Å). For the RMSF of all heavy atoms, a regular pattern was observed with the terminus showing on average larger fluctuations than the central part of the DNA mimics (Fig. 4a and b).

**Table 1:** Mean helix parameters and global flexibility of foldamers and DNA

Molecule	Twist	Rise	Bending persistence	Twist persistence	Rise fluctuations
(^m^QQ^4^)_18_	25.5°	3.66 Å	377 Å	365 Å	0.035 Å^2^ per step
(^m^QQ^5^)_18_	28.8°	3.65 Å	620 Å	489 Å	0.025 Å^2^ per step
DNA^33^	35.2°	3.30 Å	450 Å	1090 Å	—

The phosphonate groups of the side chains form the peaks in the RMSF distribution indicating larger mobility compared to atoms belonging to the aromatic and amide groups of the main chain helix (Fig. 4a and b). The phosphonate groups move mostly in a direction perpendicular to the helical axis, resulting in distance fluctuations due to transient hydrogen bond formation and longer-range repulsion due to the negative charge of the neighboring phosphonate residues (Fig. 4c and d). However, in the case of unstacking events, the distance between phosphonate groups also increases in the direction of the helical axis (see also next paragraph). In addition, there is no complete equivalence of adjacent phosphonate groups, indicating a nearest-neighbor influence of the fluctuations.

### Global bending, twisting and stretching flexibility

**Fig. 5:**
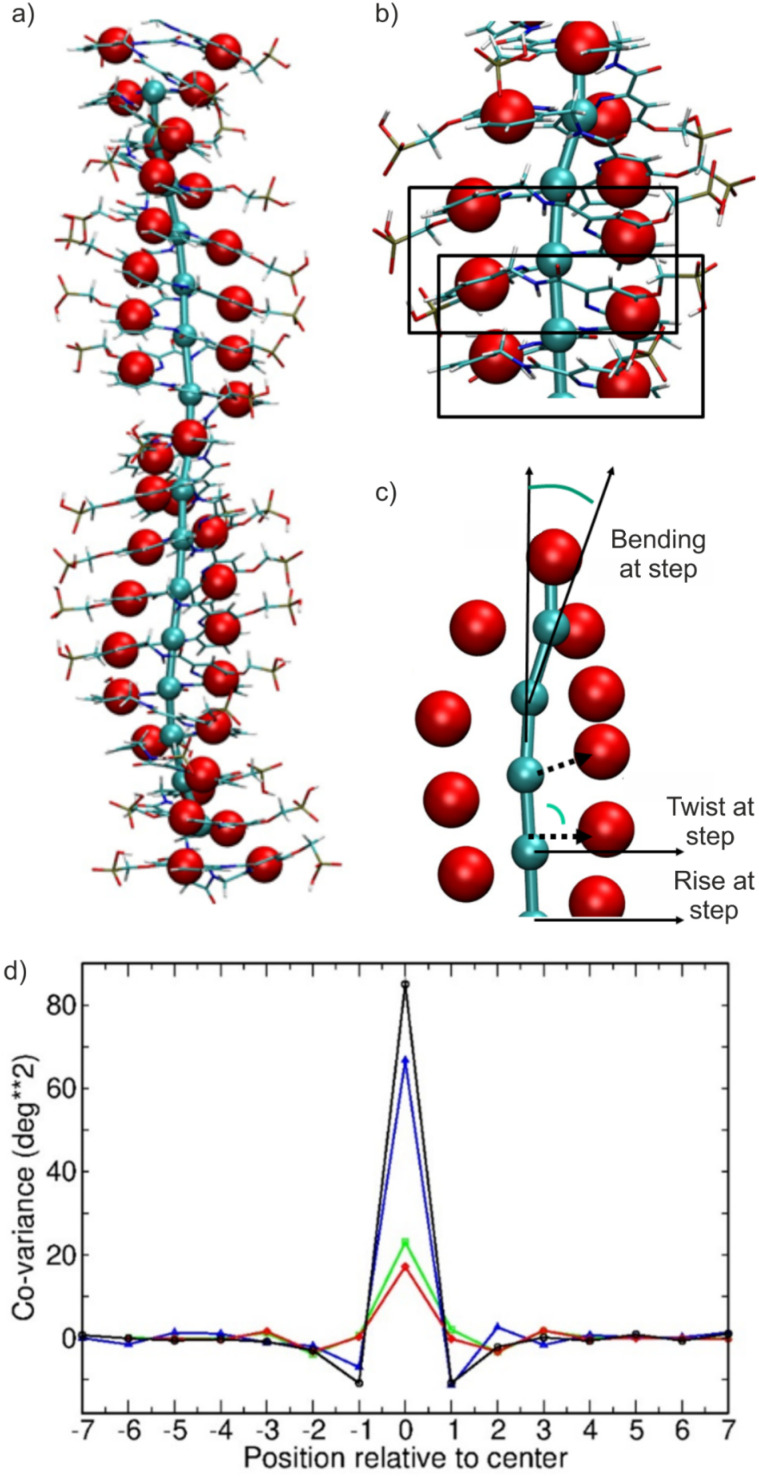
Illustration of a coarse-grained representation of the DNA mimic foldamers to calculate twisting, helical axis bending, and helical rise along the helix. (a) Superposition of an atomistic structure of (^m^QQ^4^)_18_ and of its coarse-grained representation with red spheres representing the geometric centers of the quinoline rings and cyan spheres delineating the helical axis. (b) Definition of the helical axis spheres as geometric centers of four consecutive residues, equivalent to two consecutive ^m^QQ repeat units. (c) The helical rise was calculated as the distance between consecutive spheres along the helical axis. The twist was calculated as the dihedral angle between the segments linking the helical axis and the geometric centers of aromatic rings. The bending persistence length was calculated as the scalar product of the helical direction vector at the end of the chain relative to the start of the chain. (d) Covariation of local twist and bending fluctuations. The covariance between the twist fluctuation at the central unit step (0 on the *x*-axis) of (^m^QQ^4^)_18_ (black line) and (^m^QQ^5^)_18_ (blue line) chain and the twist fluctuations at neighboring steps is plotted. Positive numbers indicate covariation in forward direction and negative numbers in reverse direction of the chain. The covariation at the center indicates the squared twist fluctuation. Local bending covariation is also indicated (^m^QQ^4^: green line; ^m^QQ^5^: red line). Local bending angles are calculated from the position of three consecutive coarse-grained centers defining the helical axis.

To calculate the global bending, twisting and stretching of the foldamer helices, we used a coarse-grained model illustrated in Fig. 5 (see also Methods section). Each quinoline ring was simplified by its geometric center (red spheres in Fig. 5). The helix axis was generated by creating coarse-grain centers (cyan spheres) each defined as the geometric center of four consecutive residues (Fig. 5b). The chain contour axis formed by these centers can be used to calculate the bending angle along the chain at each ^m^QQ unit and the distance (helical rise or pitch) between consecutive ^m^QQ units. Using the geometric centers of the quinoline rings, it is possible to define a rotation angle per ^m^QQ unit along the chain and to calculate an overall periodicity or twist of the DNA mimics. The average twist, rise (along the contour of the chain), and bending of contour elements were calculated for each trajectory (Table 1). Note that phases of the trajectories with significant structural transitions (RMSD > 6 Å, *e.g.*, as seen for ^m^QQ^4^) were excluded from the analysis.

### Transient kinking of the foldamer helix

**Fig. 6:**
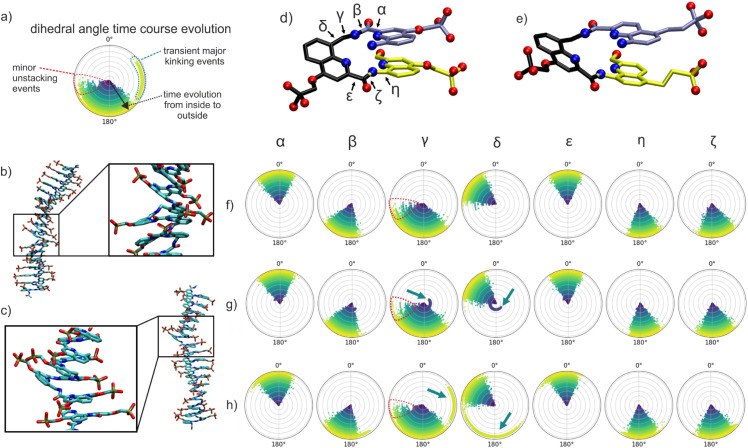
(a) Depiction of the time evolution of dihedral angle distributions during MD simulations (0° at top and 180° at bottom of the circular diagram). The start corresponds to a zero radius in each circular distribution (blue dots) and the final distribution (at end of simulation) corresponds to the largest radius (yellow dots). (b and c) Kink conformations observed during MD simulations. (b) Local unstacking event between neighboring repeating units (indicated by a rectangular box) that approximately doubles the distance between adjacent phosphonate groups along the chain. An enlarged view of the local unstacking is shown in the inset of (b). Such minor kinking events occurred transiently with lifetimes of tens of picoseconds and involved small shifts (<60°) of dihedral angles. (c) Major kinking events occurred in the simulations of the ^m^QQ^4^ DNA mimic, resulting in a transient disruption of the stacking of adjacent units (inset of (c)) and strong kinking with a break in the helical axis. Two such events with lifetimes >100 ns were observed in the simulation of (^m^QQ^4^)_18_ with singly charged phosphonates. (d and e) Chemical structures (stick model of three consecutive residues, hydrogens omitted) of a Q^4m^QQ^4^ segment (d) and a Q^5m^QQ^5^ segment (e). Relevant dihedral torsion angles along one repeat unit are indicated. Oxygen atoms are shown as red spheres and nitrogens as blue spheres. (f) Time evolution of dihedral angle distributions for a ^m^QQ^4^ unit without a major kink event during the simulation of (^m^QQ^4^)_18_ with singly charged phosphonates. The occurrence of minor kinking events is mainly associated with changes in the *γ* dihedral angle (dots encircled in red). (g) Same as (f) but for an ^m^QQ^4^ unit with a major kinking event early in the simulation that involves coupled changes of *δ* and *γ* dihedral angles (indicated by teal arrows). (h) Same as (f) for an ^m^QQ^4^ unit that underwent a major kinking event towards the final stages of the simulation (marked with teal arrows). The major kinks involved also small changes in the *ζ* dihedral angle.

Inspection of the trajectories indicates that even within the simulation parts with no major RMSD change, there are occasional local short-lived (<0.1 ns) kinking events (minor kinks) illustrated in Fig. 6b. When they are not hydrogen-bonded, the negative phosphonate groups possibly repel each other, and one can observe significant distance fluctuations of adjacent phosphonate residues. Typically, the motion occurs perpendicular to the helix axis. However, the phosphonate groups occasionally separate in the direction of the helical axis, and this leads to a local unstacking and kinking of the chain (Fig. 6b and c). The structure of the DNA mimics is mainly determined by dihedral angle rotations associated with the bonded geometry of the connections between the repeating units. These variable dihedral angles are termed *α*, *β*, *γ*, *δ*, *ε*, and *ζ* (Fig. 6d) and adopt characteristic mean values associated with modest fluctuations. An analysis of the associated changes in all dihedral torsion angles along the chain indicates that this is mostly due to a change in the dihedral angle *δ* coupled to smaller changes of the dihedral angle *γ* both in the ^m^QQ^4^ and ^m^QQ^5^ simulations (Fig. 6f). Counting all kinks (by considering all corresponding changes in *γ*) that occurred during the MD simulations resulted in 120 and 95 kinks per 1 µs simulation time for ^m^QQ^4^ and ^m^QQ^5^, respectively. Fewer kinks were observed for ^m^QQ^5^, hinting at a higher stability that was confirmed by experiments (see below).

During the ^m^QQ^4^ simulation, two reversible conformational transitions to longer lived kinked states (major kinks) were observed (large shift in the RMSD for ∼100–150 ns at time point 480 ns and 2500 ns). The associated structural changes are illustrated in Fig. 6c, g and h. It leads to a local break in the periodic structure, giving rise to two helix segments that have different directions with a kink angle of ∼40°. The observed local transient kink distortion involves coupled transitions of several dihedral angles (mostly *γ* and *δ* and smaller changes in other dihedral angles) and leads to an increased distance between phosphonate groups for three consecutive phosphonate groups along the chain (Fig. 6c). The significant dihedral angle flips mediate a transient yet longer-lived conformational state (lifetime ∼100 ns) separated by an energy barrier from the otherwise regular structure of the ^m^QQ^4^ chain. It also leads to a different partially disrupted stacked structure at the kink location (Fig. 6c). However, it eventually reverts to the original continuously stacked ^m^QQ^4^ helix. No such transition was observed during the ^m^QQ^5^ simulation, suggesting a higher stability that was verified experimentally (see below).

### MD simulation of (^m^QQ^4^)_18_ and (^m^QQ^5^)_18_ oligomers with doubly-charged phosphonates

**Fig. 7:**
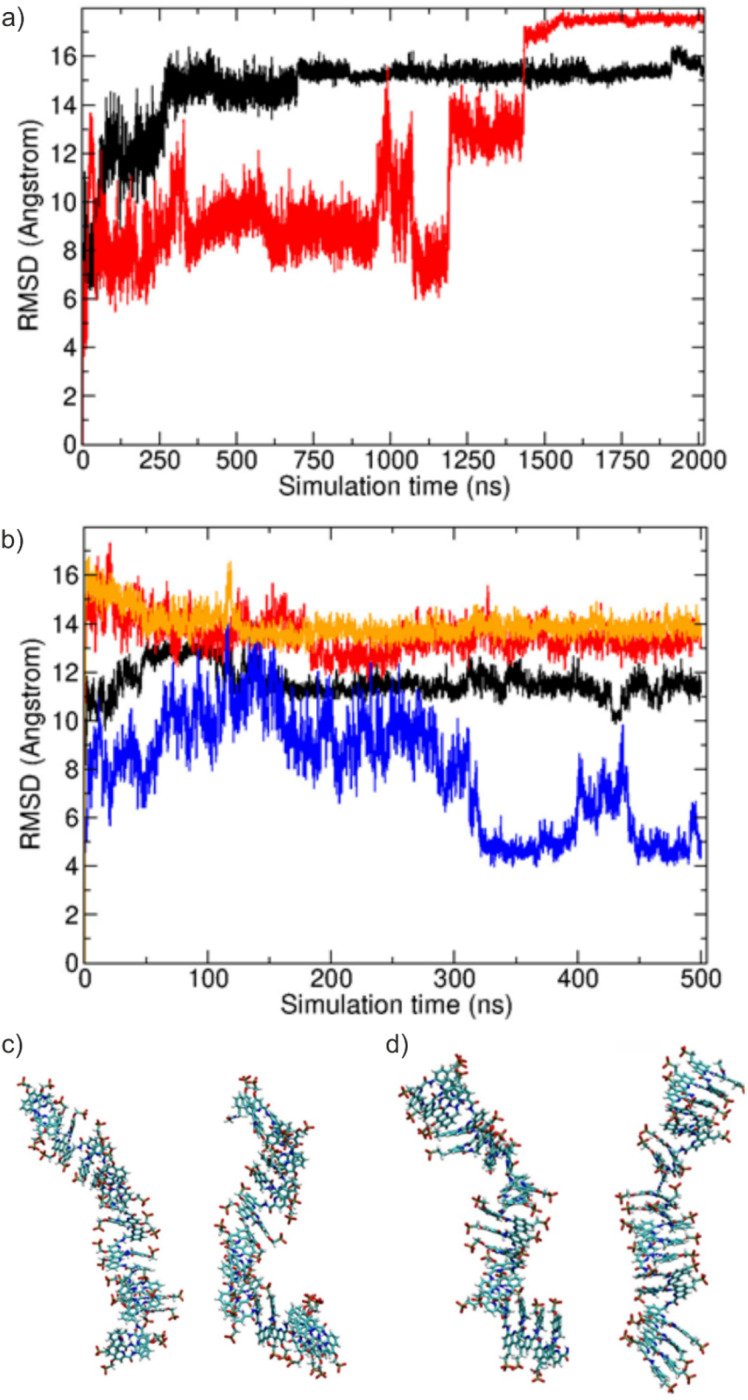
MD simulations of ^m^QQ^4^ and ^m^QQ^5^ with doubly-charged phosphonate groups. (a) RMSD (non-hydrogen atoms) from the start structure *vs.* simulation time for the ^m^QQ^4^ (black line) and for the ^m^QQ^5^ (red line) oligomers with two negative charges per phosphonate group. (b) RMSD from start structure *vs.* simulation time for ^m^QQ^4^ at high salt (0.5 M NaCl, black line), in 0.1 M LiCl (red line), ^m^QQ^5^ at high salt (0.5 M NaCl, blue line) and in 0.1 M LiCl (orange line). (c) Representative snapshots of ^m^QQ^4^ conformations (2 negative charges per phosphonate) sampled during the simulations shown in (a). (d) Same as (c) for the ^m^QQ^5^ oligomer.

In contrast to the MD simulations with singly charged phosphonate groups, the simulations with doubly charged phosphonate residues resulted in many strong kinking events along the chain and eventually in the formation of partially collapsed states with several “unstacked” connections within the ^m^QQ^4^ as well as ^m^QQ^5^ sequences (Fig. 7a, c and d). The RMSD *vs.* time shows much larger deviations from the initial regular structure compared to the simulations using single charges on the phosphonate groups. Yet again, RMSD values appeared to be lower for ^m^QQ^5^. Also, the RMSF plots indicate a much less regular pattern than for mono-protonated phosphonate residues in the chains (Fig. S1). To further investigate the potential electrostatic origin of the observed conformation transitions, we performed simulations at higher salt concentration (500 mM NaCl) or using smaller highly hydrated monovalent Li^+^ ions (100 mM LiCl). Still, for both conditions large deviations from the start structure were sampled already in the equilibration phase. However, the average deviation from the start structure was slightly smaller compared to the standard conditions (Fig. 7b). It is likely that the increased repulsion between phosphonate groups along the chain drives the unstacking and kinking events. The final often-collapsed conformations are stabilized by ion-mediated interactions between phosphonate groups (Fig. S1).

## Experimental study

### Foldamer synthesis

Foldamers 1–4 were synthesized as previously described.^11^ New chiral foldamer sequences 5–7 (Fig. 1e) were synthesized on solid phase and purified by reversed-phase high-performance liquid chromatography (HPLC) following published procedures^15,34^ from previously described building blocks.^14,35^ For sequences 6 and 7, two new building blocks – the variants of ^m^Q^OMe^ and Q^5^ suitably protected for solid phase synthesis – were necessary. Their synthesis along with the characterization of all new compounds are presented in the SI.

#### pH and salt dependence of NMR spectra

To correlate the MD results with the experimental data, 1D and 2D NMR experiments were carried out on H-(^m^QQ^4^)_8_-OH (sequence 2, with sodium as counterions) in water at pH 8 where the phosphonate side chains are in part dianionic and in a 50 mM sodium hydroxide solution at pH 12.5 in which the phosphonate side chains are all dianionic.^11^ This sequence does not contain any stereogenic center and thus exists as a racemic mixture of right-handed (*P*) and left-handed (*M*) conformers. A full assignment of the NMR spectra was not attempted but the spectra were compared to those of shorter sequence 1 for which a full assignment was reported previously.^11^ In addition, some resonances of 2 could be partly assigned using 2D COSY NMR spectra. For example, the benzylic NH resonances can unequivocally be distinguished from the aromatic NH resonances because only the former are coupled to benzylic methylene protons (Fig. S2 and S3). These scalar couplings also allow one to distinguish the latter from methylene protons belonging to the side chains.

**Fig. 8:**
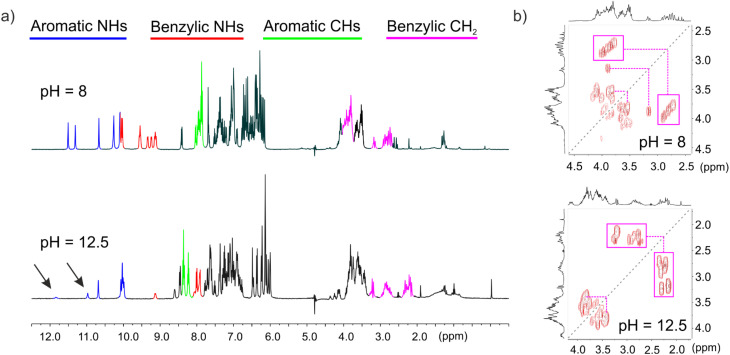
(a) ^1^H NMR spectra of 2 recorded with water suppression at 25 °C in H_2_O/D_2_O (9 : 1 v/v) at pH 8 and 50 mM sodium hydroxide H_2_O/D_2_O (9 : 1 v/v) at pH 12.5. Blue, red, green and pink lines indicate the NMR resonances of aromatic amide protons, benzylic amide protons, aromatic CH protons, and benzylic CH_2_ protons, respectively. (b) Excerpts of 2D COSY NMR spectra of 2, same solution as in (a). In both cases, diagonal suppression was applied. The spectra show strong anisochronicity (Δ*δ* > 1 ppm for some signals) of the main chain benzylic CH_2_ protons of the ^m^Q monomers that are marked with pink boxes and are upfield-shifted at pH 12.5.

The ^1^H NMR spectra of 2 at pH 8 and 12.5 show significant differences (Fig. 8a), but both are indicative of a folded helical structure. They are sharp; NMR signals are spread over a broad range of chemical shift values despite the repetitive nature of the sequence. Benzylic CH_2_ are anisochronous, consistent with their diasterotopic nature in a chiral helical structure and indicating slow exchange on the NMR timescale between *P* and *M* helices. In contrast, the benzylic methylene protons of short sequence 4 appear as a singlet indicating fast exchange between *P* and *M* helices (Fig. S4). Thus, in agreement with the MD simulations, different charge states of the side chains may give rise to changes in the helix structure and structural dynamics, but the overall helix folding is preserved, unlike in α-helical peptides that are destabilized by charge repulsions.^36^

Differences between the NMR spectra of 2 at pH 8 and 12.5 include both upfield and downfield shifts of the resonances and cannot be interpreted simply in terms of a reduction of ring current effects at higher pH due to more frequent kinking events that temporarily disrupt aromatic stacking. More aryl-CH resonances are found above 8 ppm at pH 12.5 than at pH 8 (Fig. 8a). Concomitantly, the benzylic methylene protons are upfield-shifted by over 0.5 ppm at pH 12.5, some even resonate as low as 2.2 ppm as a result of ring current effects (Fig. 8b). The benzylic amide NH resonances, but not the aromatic NH resonances, are downfield-shifted at pH 12.5. We also note that increasing pH results in a decrease of the intensity of some amide NH signals (arrows in Fig. 8a). This is due to an enhanced exchange with water, yielding suppression of the NH resonances by the water suppression part of the pulse sequence. The decrease of signal intensity is stronger with aliphatic and aromatic NHs found at lower field in both series. These signals can reasonably be assigned to amide protons near the ends of the helix, where fraying can enhance exposure to, and exchange with, water.

The effect of monovalent ions was also assessed. Spectra at pH 8 and 12 were measured in the presence of increasing concentrations of NaCl and LiCl (from 12.5 mM to 100 mM). For both salts, changes in chemical shift values were marginal compared to the changes observed upon changing pH (Fig. S5–S8). The stabilizing effects suggested by MD simulations did not apparently translate into notable changes in chemical shift values of the DNA mimic foldamers.

#### Effect of temperature

**Fig. 9:**
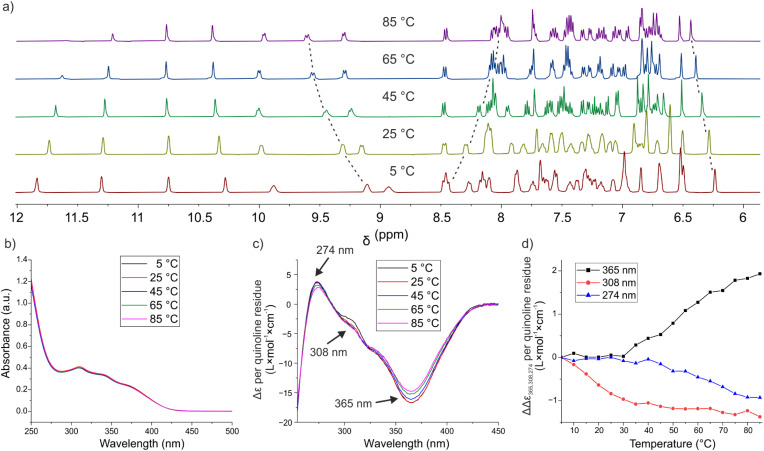
(a) Excerpts of the ^1^H NMR spectra from 12.0 to 6.0 ppm of H-(^m^QQ^4^)_4_-OH (compound 1) recorded in 50 mM ammonium bicarbonate in H_2_O/D_2_O (9 : 1 v/v) from 5 °C to 85 °C. (b) Variable temperature UV-vis spectra of (^m^QQ^4^)_4_ in 10 mM KH_2_PO_4_ (pH 7.0), 20 mM KCl buffer. (c) Variable temperature CD spectra of compound 5 in 20 mM Tris (pH 7.5), 100 mM NaCl. (d) Variations of absorbance as a function of temperature extracted from the experiment shown in (c) for three different wavelengths. Variations are with respect to values recorded at 5 °C.

We investigated the effect of temperature. Even though single-stranded foldamers cannot dissociate in the way double-stranded B-DNA does, increasing temperature should enhance structural dynamics, *e.g.* the kinking events observed in MD simulations, and this may be reflected in their spectroscopic properties. We therefore analyzed 1 by variable temperature (VT) UV-vis and NMR measurements (Fig. 9). NMR spectra showed some chemical shift variations suggesting structural changes such as differences in helix dynamics or altered helix curvature, but no sign of unfolding at temperatures up to 85 °C (Fig. 9a). The signals of some amide protons shift more than others (Δ*δ* ranging from 0.1 ppm or less to *ca.* 0.5 ppm), which may reflect fraying at the end of the helix. The reduced intensity of some NH resonances was assigned to enhanced exchange with water at higher temperature together with the water signal suppression. Concomitantly, UV-vis spectra were essentially unchanged over the same temperature range (Fig. 9b), in great contrast with the typical melting curves of B-DNA duplexes. Finally, we measured the CD spectra of chiral sequence 5 (the helix of 5 is left-handed, see next section) at various temperatures (Fig. 9c). CD is a particularly sensitive method to detect changes in the chiral environment of chromophores, and some small variations of CD band intensities were observed, but the trends were different at different wavelengths (Fig. 9d), suggesting that the changes reflect local variations rather than a transition from one state to another.

#### Quantitative assessment of helix stability

The NMR and UV-vis measurements presented above gave qualitative insights about how some of the structural dynamics observed in MD simulations translate into variations of the spectroscopic properties of the DNA mimic foldamers. We next sought a quantitative assessment of foldamer helix stability. We have previously assessed the stability of aromatic foldamer helices by measuring the rate of interconversion between *P* and *M* helical conformations.^20,21,37^ For organic-soluble Q_*n*_ oligomers, it was possible to separate the *P* and *M* enantiomeric conformers of an achiral sequence using HPLC on a chiral stationary phase at low concentration. Racemization of a one-handed conformer was then monitored by observing the decay of circular dichroism absorption bands.^20,21^ For water soluble oligomers containing Q units, another approach was developed where a chiral group is introduced in the sequence that biases helix handedness in favor of *P* or *M* diastereomeric conformations in a solvent dependent manner.^38,39^ Upon incubating the sequence in a first solvent, a certain *P*/*M* ratio was reached at equilibrium that translates into CD bands of a certain intensity. When that solvent was removed and the sequence dissolved in a new solvent, the CD bands intensity changes can be monitored as the *P*/*M* ratio readjusts to another equilibrium value in that new solvent.

**Fig. 10:**
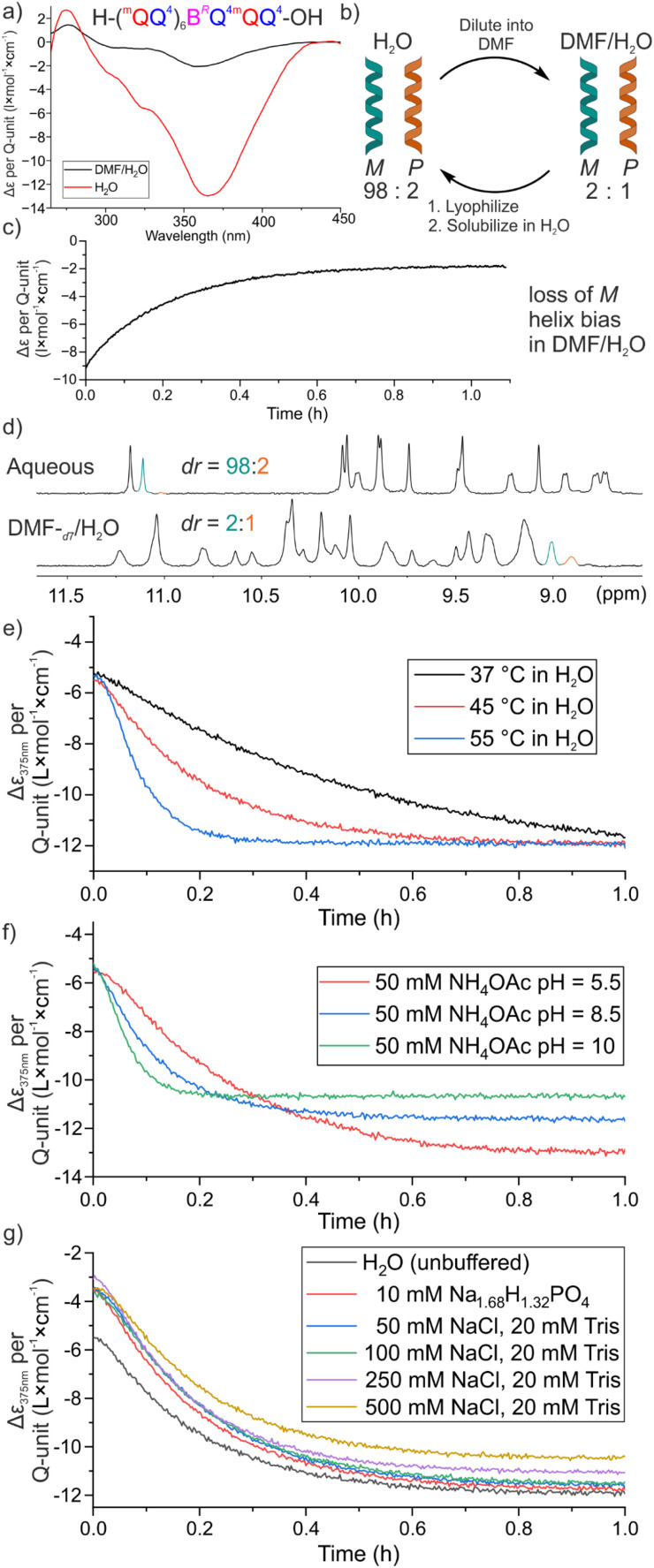
(a) Chiral DNA mimic foldamer sequence 5 with the chiral unit incorporated (top) and its per quinoline normalized CD spectra in aqueous solution and DMF/H_2_O at 25 °C. (b) Schematic display of diastereomeric enrichment assay to quantify foldamer helix dynamics. (c) Time-dependent CD monitoring at 375 nm of a sample initially dissolved in water immediately after its dilution with 9 times its volume of DMF displaying enrichment of the foldamer *P* helix. (d) Excerpts of ^1^H NMR spectra in NH_4_HCO_3_ (50 mM in H_2_O/D_2_O, 9 : 1; v/v) buffer (top) and DMF-*d*_7_/H_2_O (9 : 1; v/v) of the amide region from 8.5 to 11.5 ppm. (e) Monitoring *M* helix enrichment in H_2_O at varying temperatures. (f) Monitoring *M* helix enrichment in an NH_4_OAc buffer system at different pH at 45 °C. (g) Monitoring *M* helix enrichment at different salt concentrations. All samples were buffered at pH 7.5, unless stated otherwise.

We thus developed such an assay for (^m^QQ)_*n*_ helices. It was previously shown that introducing a single chiral B^*R*^ unit (Fig. 1a) in an (^m^QQ)_*n*_ sequence strongly biases helix handedness in water.^14,34^ For example, in an ammonium bicarbonate buffer, the ^1^H NMR spectrum of sequence 5 (Fig. 1e, ^m^QQ^4^ series) shows two distinctive sets of signals corresponding to the *P* and *M* diastereomeric conformers in a ratio of 98 : 2 (Fig. 10d). This strong bias is reflected in the CD spectrum with an intense negative band with a maximum at 365 nm as a result of the preferred *M* helicity (Fig. 10a). We screened a small number of water-miscible organic solvents and found that in a DMF/H_2_O mixture (9 : 1, v/v), that is, with enough water to maintain solubility, the CD band is much less intense. Dissolving a sample of 5 in this solvent and monitoring the CD spectrum showed a clear drop in intensity (Fig. 10a–c), an effect that was not observed by 10-fold dilution in water (Fig. S9). The NMR spectrum in DMF-*d*_7_/H_2_O showed that the proportion between the two diastereomeric conformers is reduced to *ca.* 2 : 1 in favor of the *M*-helix (Fig. 10d), consistent with the intensity of the CD bands. It is interesting to point that similar effects were not observed with Q_*n*_ oligomers with which a single B^*R*^ unit was shown to quantitatively bias handedness in water, methanol, DMSO, and chloroform.^34,40^ We hypothesize that the helices of ^m^QQ^4^ oligomers are destabilized in organic solvents when hydrophobic effects are weakened (here in 90% DMF) due to the flexibility of the main chain benzylic methylene groups.^41,42^ The extent of destabilization is sufficient to lower the effectiveness of B^*R*^ at biasing helix handedness. We repeated these experiments with sequence 6 (Fig. 1e, ^m^QQ^5^ series) and found that the diastereomeric ratio was 9 : 1 in favor of the *M*-helix in DMF/H_2_O (9 : 1, v/v) *vs.* at least 98 : 2 in water. The drop in CD intensity was thus much weaker (Fig. S10 and S11). Consistent with the observations presented both above and below, ^m^QQ^5^ oligomers appear to be more stable than ^m^QQ^4^ oligomers. Here, this is reflected in a weaker destabilizing effect of DMF that results in a stronger handedness bias by B^*R*^. Nevertheless, even if the drop in CD intensity was weaker in the ^m^QQ^5^ series, resulting in noisier data and lower quality fits (Fig. S12), it was sufficient to monitor helix handedness inversion in water.

Experiments were thus implemented where a solution of B^*R*^-containing foldamers 5 or 6 was first let to equilibrate in DMF/H_2_O (9 : 1 v/v), then diluted in water and immediately frozen with liquid nitrogen and lyophilized to obtain a mixture of the *P* and *M* diastereomeric conformers in solid form. After dissolving this material in water at different pH, salt concentration, and temperature, the increase of CD intensity was monitored as a function of time as helix handedness inversion takes place to reach the new equilibrium diastereomeric ratio (Fig. 10). The data were fitted to a single-exponential decay function (1) to obtain their respective half-lives by eqn (2).1
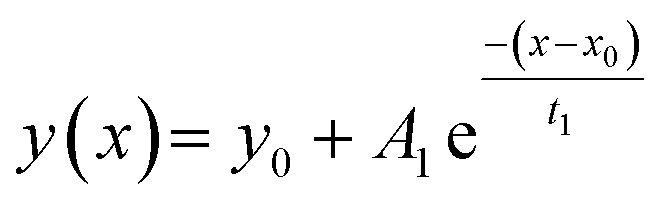
2
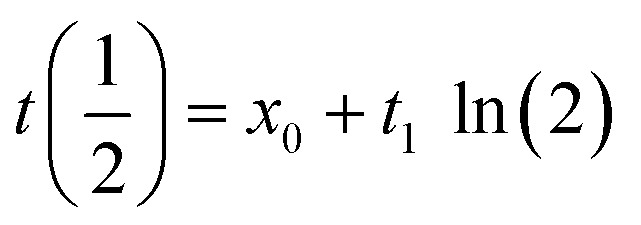


First, we analyzed the effect of temperature on helix handedness dynamics (Fig. 10e, S13, Table 2). Half-lives ranged from minutes at 55 °C to hours at 20 °C, highlighting the considerable stability of ^m^QQ helices, and explaining why the equilibrium between *P* and *M* helices is slow on the NMR timescale unless the oligomer is very short (as for 4, see Fig. S4). The results also consistently showed the higher stability of ^m^QQ^5^ sequences with respect to ^m^QQ^4^ sequences. To keep balance between measurement time and accuracy, further measurements were carried out at 45 °C. Upon fitting the data, the initial time for the sample to reach 45 °C was accounted for by cutting the data off until an exponential decay was observed.

**Table 2:** Half-lives of helix handedness inversion of 5 and 6 at different temperatures in H_2_O

*T* (°C)	20	37	45	55
5 (^m^QQ^4^ series)	207 min	22.3 min	9.4 min	4.6 min
6 (^m^QQ^5^ series)	526 min	66.1 min	18.5 min	8.1 min

We then varied the pH from 5.5 to 10 (Fig. 10f, S14, Table 3) using adequate buffer systems. Increasing pH led to significantly faster helix handedness inversion kinetics, which appears to be in line with the stronger conformation dynamics observed in simulations. The destabilizing effect of increasing pH probably reflects an increase of electrostatic repulsions between phosphonate side chains when they are doubly charged.

**Table 3:** Half-lives of helix handedness inversion of 5 at different pH at 45 °C

pH	5.5	8.5	10
5 (^m^QQ^4^ series)	207 min	22.3 min	9.4 min

Nevertheless, one should keep in mind that the foldamer helices are still fully folded at high pH as reflected by their NMR spectra (Fig. 8) and their CD spectra (Fig. S14). Furthermore, the results presented below show that the effect of charges is more complex than just electrostatic repulsions. For instance, adding salt (NaCl up to 500 mM) had little effect on the helix handedness kinetics (Fig. 10g, Table 4), in line with the small effects seen on NMR spectra. Of note, adding Ca^2+^ or Mg^2+^ divalent cations led to precipitation of the foldamers.

**Table 4:** Half-lives of helix handedness inversion of 5 at different salt concentrations at 45 °C

[Na^+^] in mM	0	16.8	50	100	250	500
5 (^m^QQ^4^ series)	9.4 min	9.8 min	9.4 min	10.7 min	9.5 min	10.5 min

**Fig. 11:**
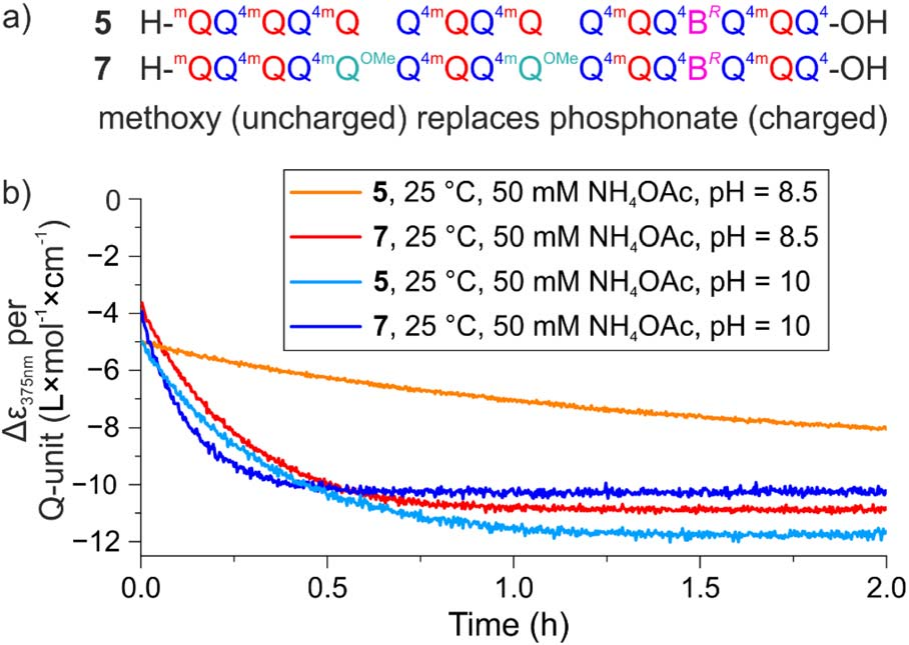
(a) Chiral DNA mimic foldamer sequences 5 and 7. Compared to 5, 7 has two side chain phosphonic acids replaced by methoxy side chains that lack charged residues. (b) Monitoring of *M* helix enrichment of 5 and 7 in NH_4_OAc-buffer at 25 °C, pH 8.5 and pH 10.

To further investigate how the side chains and their charge state influence helix conformational dynamics, we prepared sequence 7, an analogue of 5 in which two phosphonic acid-containing ^m^Q residues have been replaced by ^m^Q^OMe^ residues, whose side chains consist of a simple methoxy group (Fig. 1 and 11a). Helix handedness inversion was eventually found to be faster with 7 than with 5, so that measurements had to be carried out at 25 °C because they were too fast to measure at 45 °C. The half-life of helix handedness inversion at pH 8.5 was calculated to be 11.2 min for 7 compared to 54.6 min for 5 (Fig. 11b, S15). The same trend was observed at pH 10, though kinetics were overall faster (7: *t*_1/2_ = 5.5 min, 5: *t*_1/2_ = 14.0 min). The fact that removing charges gives rise to faster helix handedness inversion kinetics shows that the negatively charged side chains do not have an exclusively destabilizing effect. Whether this directly relates to hydrogen bonding between side chains observed in MD simulations (Fig. 4) remains to be demonstrated.

## Discussion

Several notable trends emerge from the combined computational and experimental investigations presented above. One important trend is that DNA mimic foldamers have an overall (in)flexibility, *e.g.*, bending persistence and twist persistence, similar to that of B-DNA despite their completely different chemical constitution (Table 1). The distinct backbone of DNA mimic foldamers is associated with local fluctuations and kinks that are unique to them, but their overall shape persistence – not just their overall shape – nevertheless resembles that of DNA. Yet important differences include the absence of melting behavior in the DNA mimics, beyond the fact that they are single-stranded and cannot dissociate like DNA duplexes. Heating certainly enhances their internal dynamics but no transition to an unfolded state was observed and both CD and NMR indicate that the DNA mimics are still helically folded at 85 °C.

**Fig. 12:**
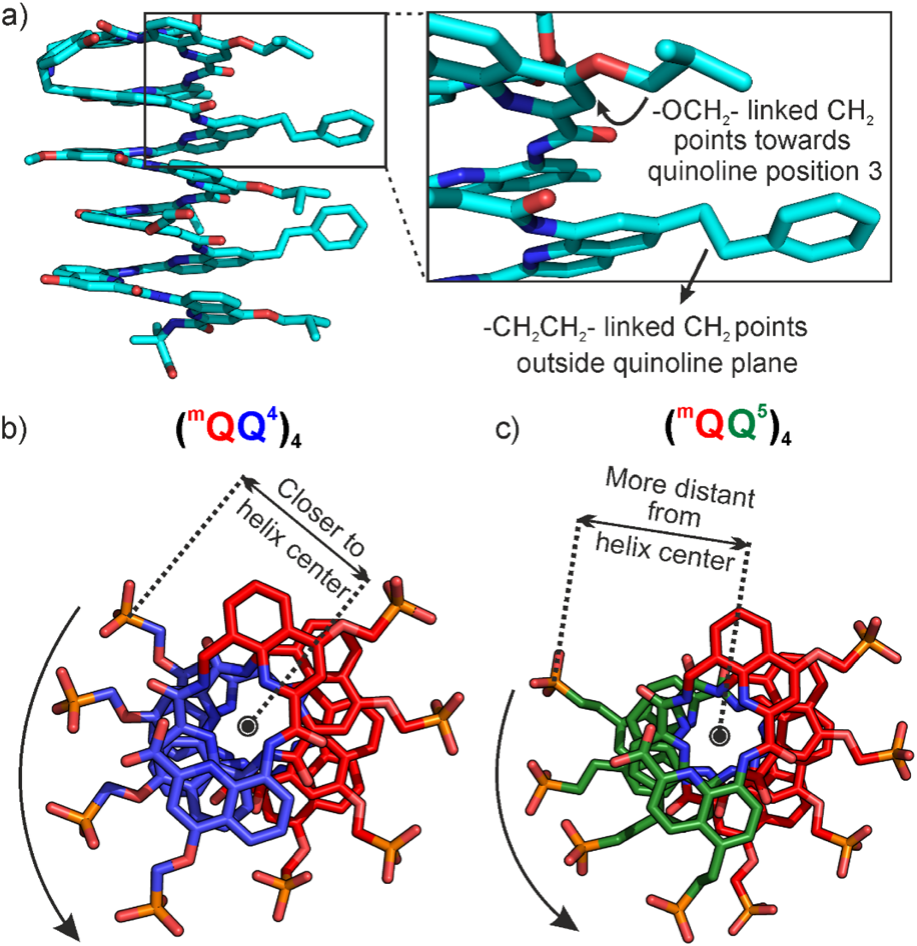
(a) Crystal structure of a foldamer^43^ that has side chain attached by both –CH_2_CH_2_– and –OCH_2_– linkages. Typical orientation of CH_2_ bridges are depicted in the close-up. Crystal structures of diethyl phosphonate ester protected Q^4^ and Q^5^ based octamers (b) and (c).^11^ Diethyl esters as well as C- and N-terminal protecting groups are omitted for clarity. The distance between Q^4^ side chains at *i*, at *i* + 2 and *i* − 2 positions is smaller than the distance between Q^5^ side chains at *i*, at *i* + 2 and *i* − 2 positions.

A second trend is the consistent observation that the ^m^QQ^5^ oligomers are more stable than the ^m^QQ^4^ oligomers. This is reflected in a lesser occurrence of kinks during MD simulations, and in longer bending and twist persistence along the ^m^QQ^5^ helix (Table 1). It is also reflected in the lower destabilizing effect of an organic solvent such as DMF, resulting in a higher helix handedness bias in chiral ^m^QQ^5^ sequence 6 compared to ^m^QQ^4^ sequence 5 in that solvent. In addition, helix handedness inversion is slower in ^m^QQ^5^ helices, regardless of the temperature considered (Table 2). Though it is not easy to directly relate these effects to the structural differences between Q^4^ and Q^5^, it is possible to highlight these differences. The reason why the phosphonic acids are not connected to the quinoline ring with the same linker in Q^4^ (–OCH_2_–) and in Q^5^ (–CH_2_CH_2_–) is not a design consideration but the result of different synthetic approaches. Yet it gives rise to distinct structural properties. The ethylene linker is less polar and more hydrophobic than the oxymethylene linker, possibly decreasing affinity for water molecules that fill the space between neighboring aromatic units during unstacking events. Furthermore, the two linkers have different conformational preferences. We have obtained the solid-state structures of numerous foldamer helices with Q units bearing a side chain in position 4 connected by an –OCH_2_– linker. The general observation is that the CH_2_ carbon is found in the plane of the quinoline ring and close to position 3 (opposite to position 5). In contrast, the same carbon in –CH_2_CH_2_– linkers is generally found out of the plane of the quinoline ring (Fig. 12a).^43^ Another difference between Q^4^ and Q^5^ concerns the protrusion of their side chain from the helix. As mentioned in the introduction, the design of Q^5^ was driven by the need for DNA mimic foldamers with a wider major groove and a narrower minor groove (Fig. 1).^11^ Yet a difference that was not highlighted before is that phosphonic acids lie closer to the helix axis in Q^4^ monomers than in Q^5^ and ^m^Q monomers. This is clearly visible in top views of solid-state structures and molecular models (Fig. 12b and c). That the side chains of Q^4^ residues are somewhat closer to the helix axis implies that they are also slightly closer to each other.

A third trend is the strong effect of pH on the DNA mimic foldamer structural dynamics. Increasing the proportion of doubly charged phosphonate residues – *i.e.*, increasing pH – results in more frequent kinks in MD simulations. It is also reflected in changes in NMR spectra though these confirm that helix folding is maintained even at pH > 12. Increasing pH also enhances the kinetics of handedness inversion. However, these effects cannot all be interpreted simply in terms of electrostatic repulsions between negatively charged side chains. First, adding monovalent salts had little effect on the NMR spectra and on the rate of helix handedness inversion. Second, removing some negatively charged side chains resulted in faster helix handedness inversion. To interpret this fact, one must consider that the kinetics of helix handedness inversion are not directly the reflection of helix stability as would be the transition between a folded and an unfolded state. Instead, they reflect the energy difference between the folded state and a higher energy state enabling handedness inversion. Modeling studies suggested that this higher energy state is a helix with a local misfold – a local reversal of handedness can be generated by flipping two residues – that can propagate along the helix.^21,44^ It may well be that these misfolded states involve energetically costly contacts between phosphonate side chains and hydrophobic quinoline faces. When using a short nonpolar side chain as in ^m^Q^OMe^, more favorable contacts may occur, leading to a stabilization of these intermediate states and thus to faster kinetics.

## Conclusion

DNA mimic foldamers, aromatic oligoamides composed of ^m^QQ^4^ or ^m^QQ^5^ repeat motifs that adopt helical conformations reproducing the structure and charge distribution of B-type double-stranded DNA, have recently been designed. These molecules are promising candidates as competitive inhibitors of protein-DNA complexes and have potential to serve as pharmacological tools. We have investigated their structural dynamics both computationally and experimentally. For this purpose, force field parameters were optimized and an assay to experimentally assess helix stability was developed.

The helical conformations of the DNA mimic foldamers are stable over a wide range of temperature and pH in aqueous solutions and no melting behavior was observed upon heating. The ^m^QQ^5^ oligomers show a higher stability than the ^m^QQ^4^ oligomers. MD simulations revealed different types of kinking in the foldamer helices associated with more or less long-lived unstacking events between the aromatic monomers. At higher pH, when the side chains are doubly negatively charged, kinking events become more frequent and lasting. This resulted in a chain containing small helical segments interrupted by kinked steps and an overall irregular conformation but in which the overall helix integrity – its handedness – was preserved, unlike in polyanionic or polycationic α-helical peptides. The structural dynamic parameters of the DNA mimic helices, including bending, twisting and stretching flexibility, were found to be in the same range as those of DNA. This result extends the resemblance between double stranded B-DNA and DNA mimic foldamer beyond structure to some dynamic properties. This resemblance is remarkable considering that they are based on completely different backbones and that local conformational changes associated with these backbones are also completely different.

Progress is being made both in the structural elucidation of DNA mimic foldamer-protein complexes^17^ and in the production of hybrid molecules combining a foldamer and a DNA segment.^16^ In this context, the results and methods reported here will be useful to design distinct foldamers that specifically recognize DNA-binding proteins as well as other backbones that may also mimic B-DNA or other nucleic acid structures.

## Author contributions

ML, JW, and VC performed syntheses and experimental studies. LT, ZL, and MZ performed computations. VP, MZ and IH supervised the research. ML, VC, LT, MZ and IH wrote the manuscript. All authors reviewed and edited the manuscript and approved its final version.

## Conflicts of interest

There are no conflicts to declare.

## Supplementary Material

SC-017-D5SC08567E-s001

## Data Availability

The data that support the findings of this study are available from the corresponding author upon reasonable request. Supplementary information (SI): SI figures, detailed experimental protocols, characterization of new compounds. See DOI: https://doi.org/10.1039/d5sc08567e.
