# Supplementary material for: Structural dynamics of DNA mimic foldamers
Source: Chem Sci. 2025 Dec 8;17(6):3198–211. doi: 10.1039/d5sc08567e (PMC12709542; doi:10.1039/d5sc08567e)
Supplement: SC-017-D5SC08567E-s001 [file SC-017-D5SC08567E-s001.pdf]

Supplementary Information (SI) for Chemical Science.
This journal is © The Royal Society of Chemistry 2025

Supplementary information for:

Structural dynamics of DNA mimic foldamers

Manuel Loos, Lion Thurecht, Jiaojiao Wu, Valentina Corvaglia, Zhiwei Liu, Vojislava Pophristic, Martin Zacharias and Ivan Huc

Table of contents

1 Supplementary figures 2

2 Materials and Methods 11

2.1.1 Materials 11

2.1.2 Small molecule nuclear magnetic resonance spectroscopy and mass spectrometry 11

2.1.3 Oligomer NMR spectroscopy 11

2.1.4 RP-HPLC chromatography and LC-MS 11

2.1.5 Circular dichroism (CD) and UV-Vis absorption spectroscopy 12

2.2 Foldamer building blocks 13

2.3 Synthetic procedures 13

2.4 Foldamer synthesis 18

2.4.1 General solid-phase foldamer synthesis 18

2.4.2 Removal of the diethyl-phosphonate protecting groups 19

2.4.3 Cation exchange chromatography 20

2.4.4 Synthesized foldamers 20

3 Spectra & Chromatograms 23

3.1 NMR spectra 23

3.1.1 Small molecules spectra 23

3.1.2 Oligomer NMR spectra 28

3.2 HPLC chromatograms 31

3.3 Mass spectra 33

3.3.1 Foldamer mass spectra 33

4 References 35

1 Supplementary figures

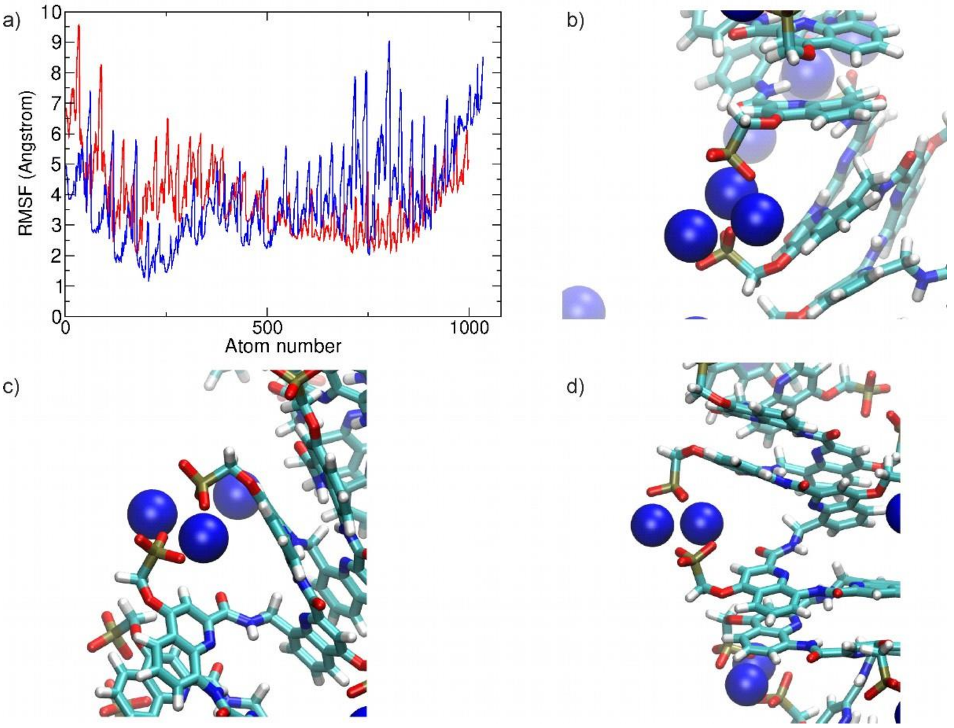

Figure S 1 (a) Root-mean-square fluctuations (RMSF) of non-hydrogen atoms *vs.* atom number observed during ($^{m}QQ^{4}$)$_{18}$ simulations (blue line) and ($^{m}QQ^{5}$)$_{18}$ simulations (red) with doubly charged phosphonate groups. (b-d) Simulation snapshots indicating trapped binding of several sodium ions (blue spheres) between double anionic phosphonate groups (stick models, $^{m}QQ^{4}$ case).

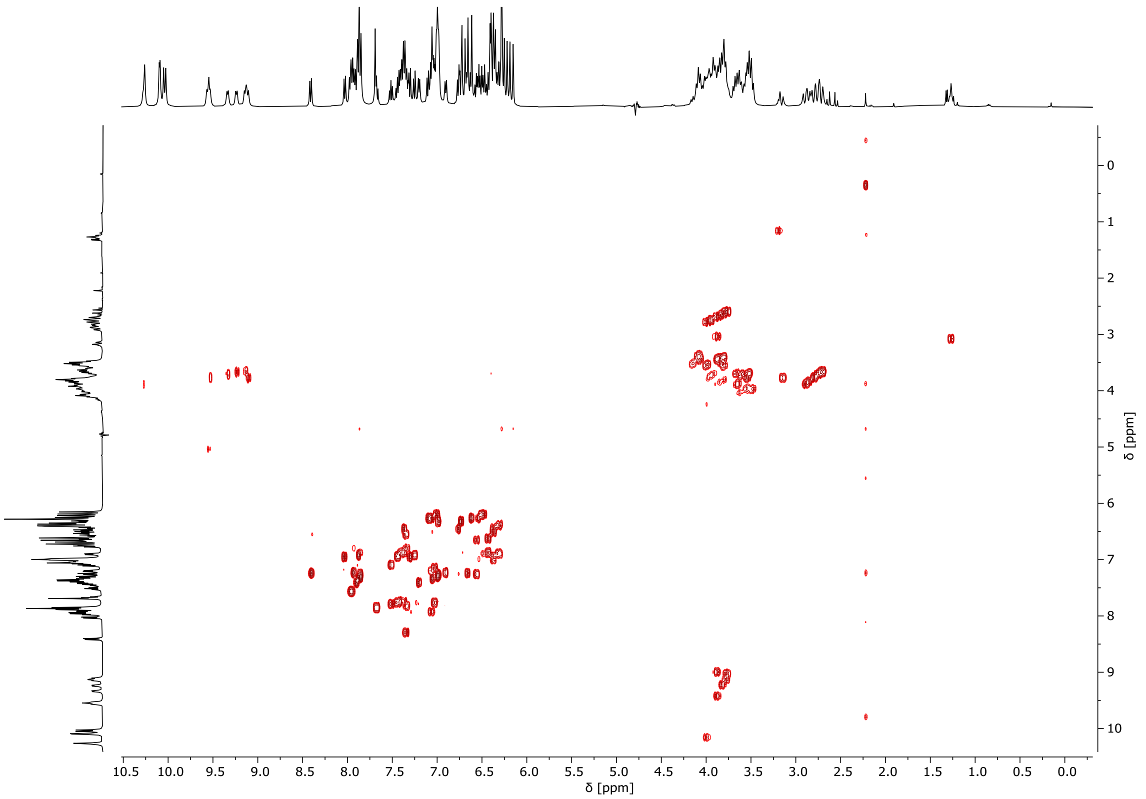

Figure S 2 2D COSY NMR spectrum of **2** recorded with water suppression at 25 °C in H_2O/D_2O (9:1, v/v). After dissolving, the solution was measured to be at pH 8.

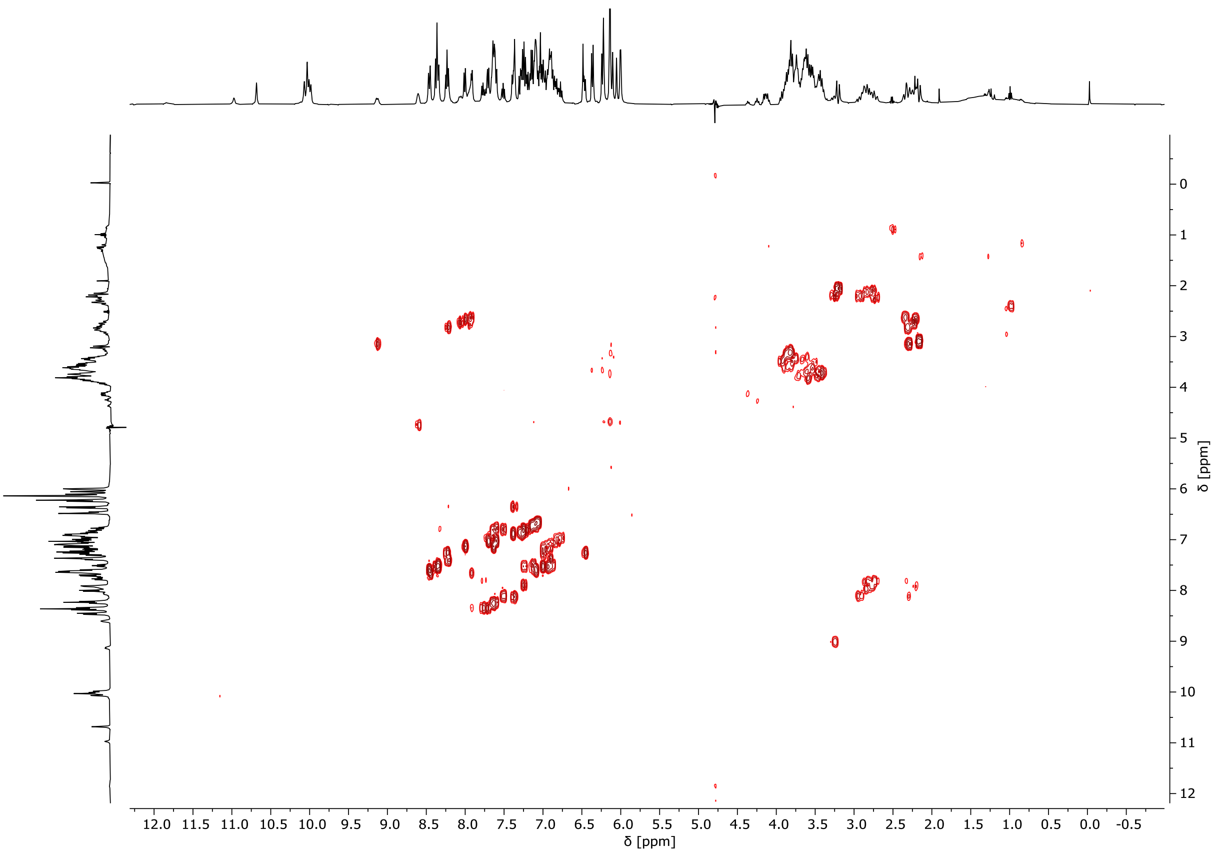

Figure S 3 2D COSY NMR spectrum of **2** recorded with water suppression at 25 °C in 50 mM sodium hydroxide H_2O/D_2O (9:1, v/v) at pH 12.5**.**

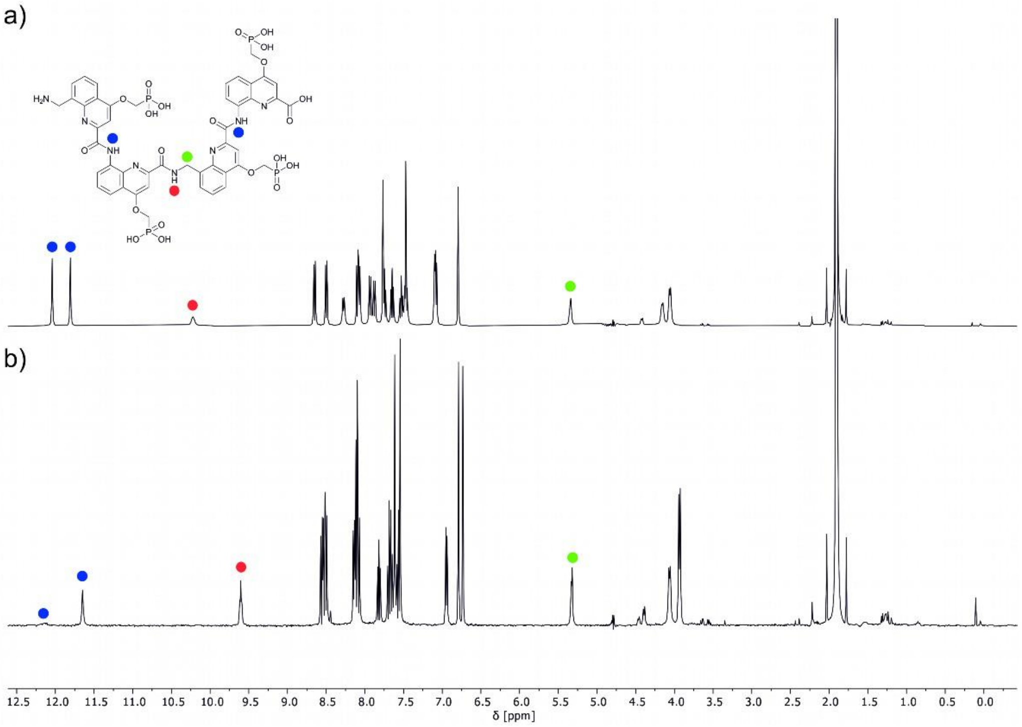

Figure S 4 ^{1}H NMR spectra of **4** recorded with water suppression at 25 °C in H_2O/D_2O (9:1, v/v) at pH 8 (a) and 50 mM sodium hydroxide H_2O/D_2O (9:1 v/v) at pH 12.5 (b). Blue, red and green balls indicate aromatic amide protons, benzylic amide protons and benzylic CH_2 protons, respectively.

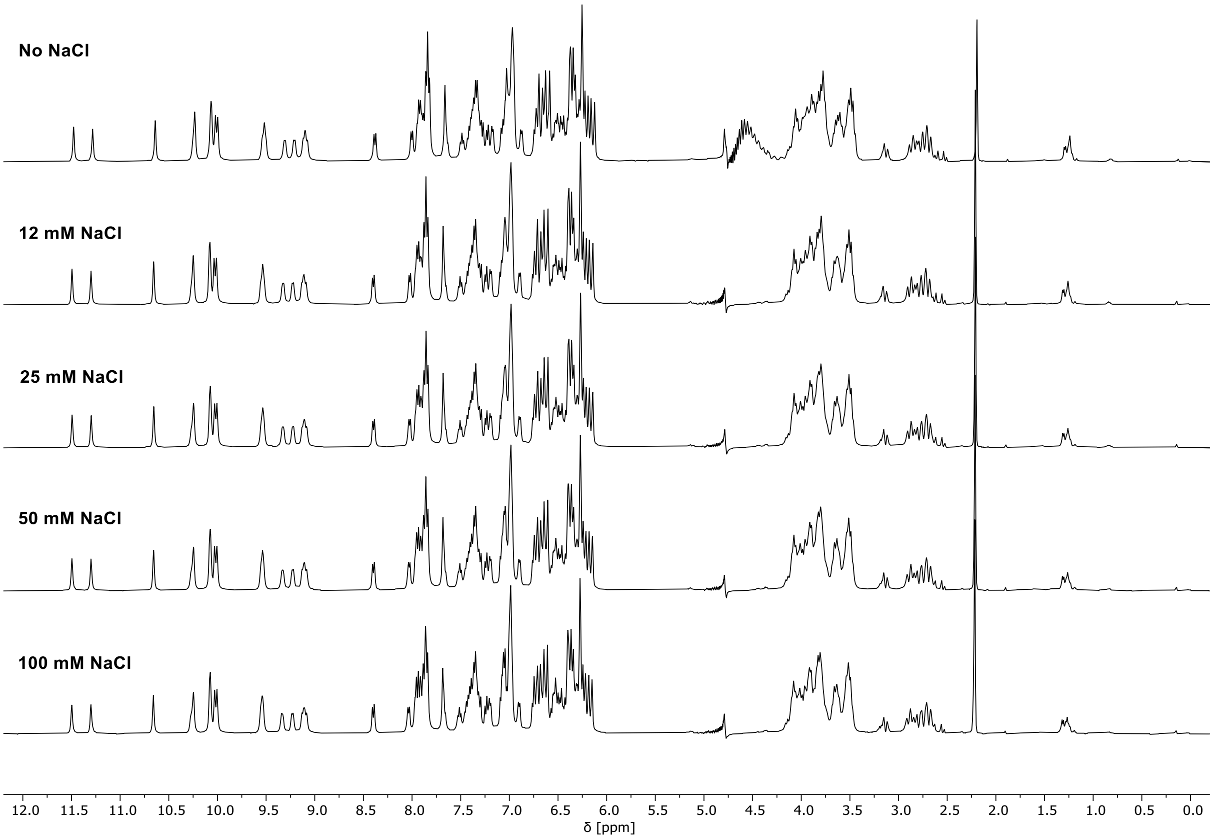

Figure S 5 ^1H NMR spectra of **2** recorded with water suppression at 25 °C in H_2O/D_2O (9:1, v/v) at pH 8, with increasing NaCl concentration.

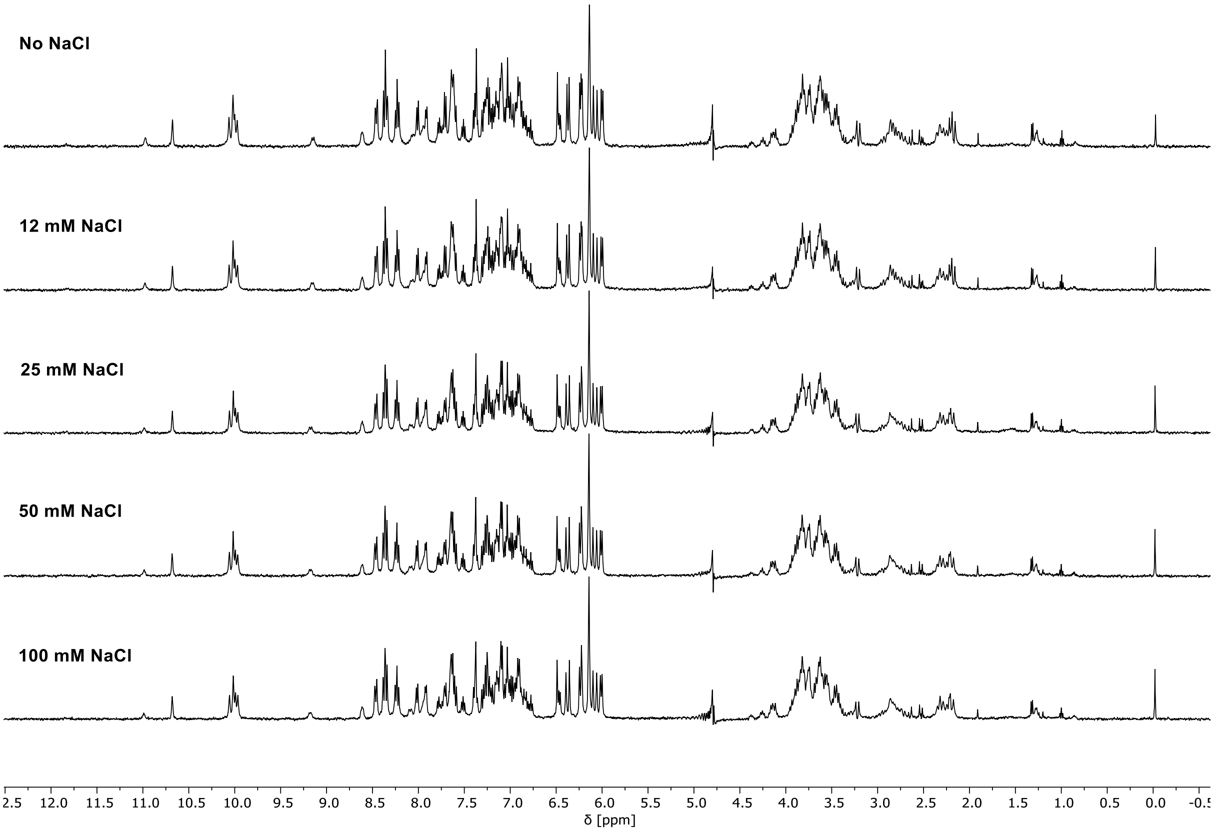

Figure S 6 ^1H NMR spectra of **2** recorded with water suppression at 25 °C in H_2O/D_2O (9:1, v/v) at pH 12.5, with increasing NaCl concentration.

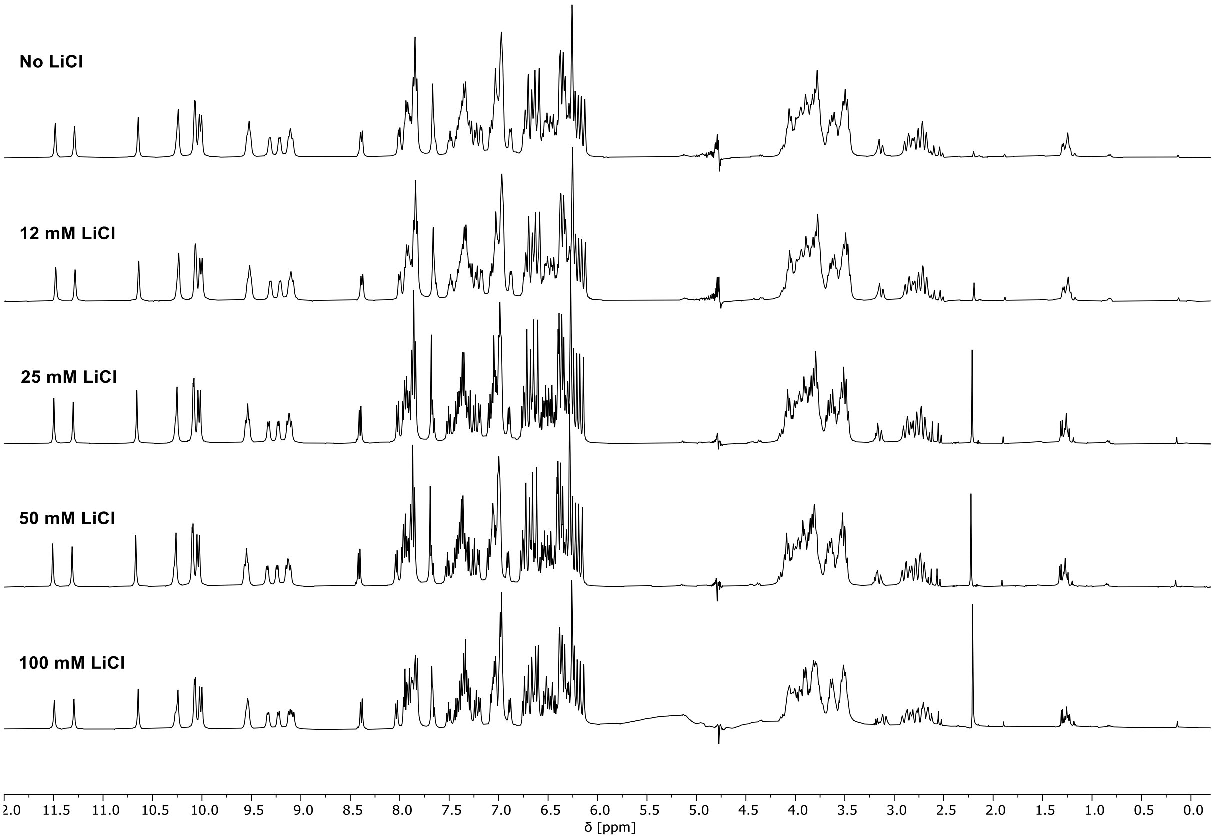

Figure S 7 ^{1}H NMR spectra of **2** recorded with water suppression at 25 °C in H_2O/D_2O (9:1, v/v) at pH 8, with increasing NaCl concentration.

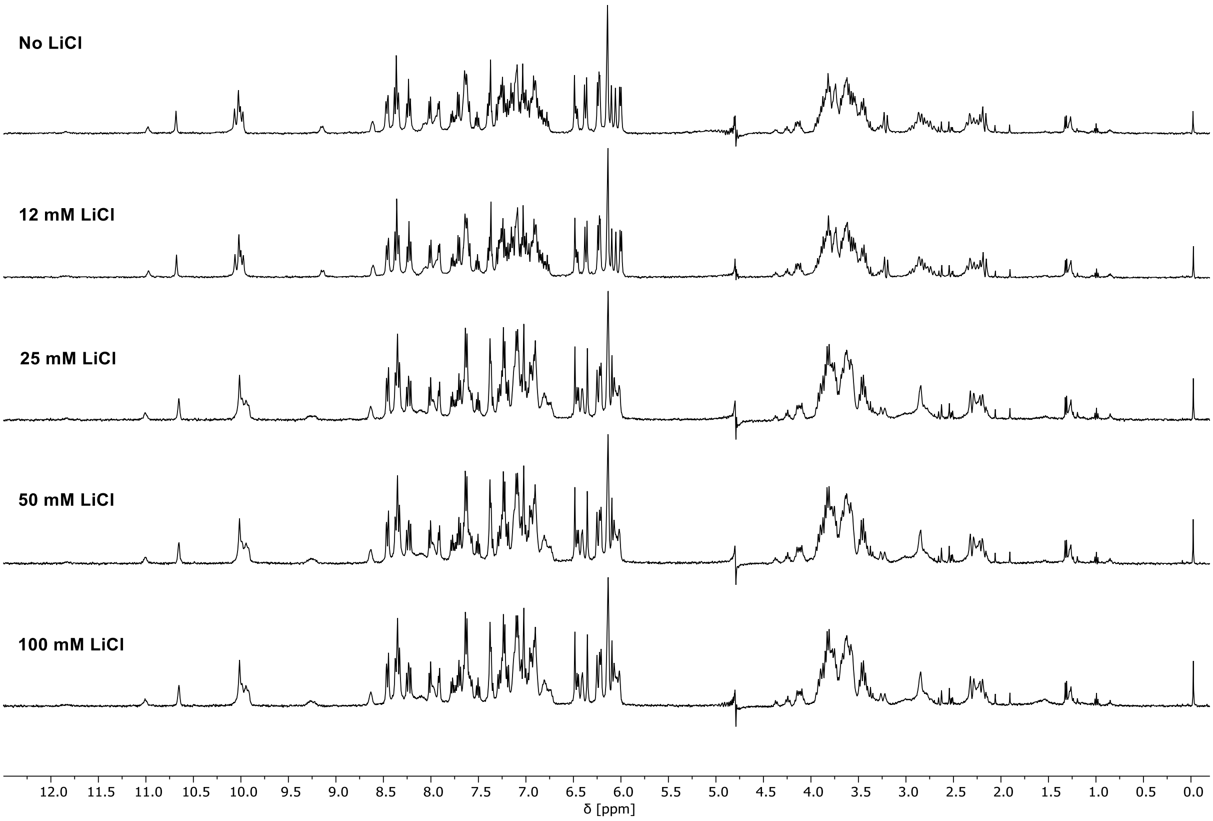

Figure S 8 ^{1}H NMR spectra of **2** recorded at 25 °C in H_2O/D_2O (9:1 v/v) at pH 12.5 with increasing LiCl concentration.

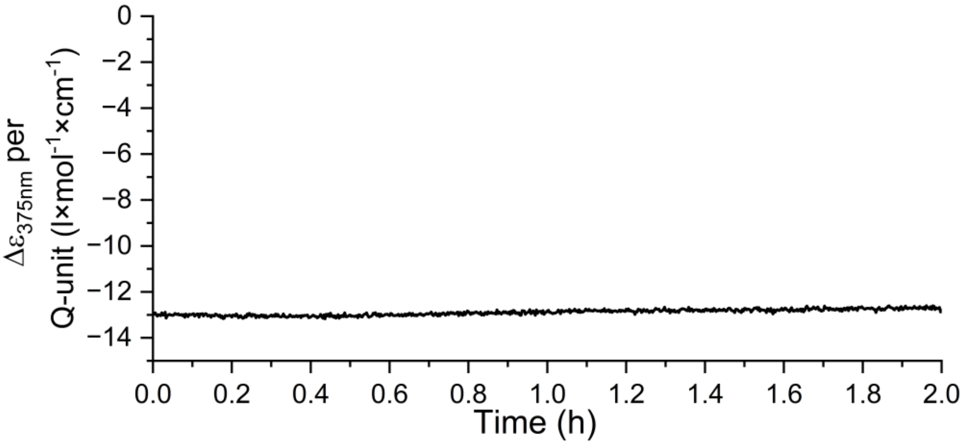

Figure S 9 Time-dependent monitoring of the CD-band at 375 nm of compound **5**, which was dissolved in H_2O and freshly diluted into nine times its volume of H_2O.

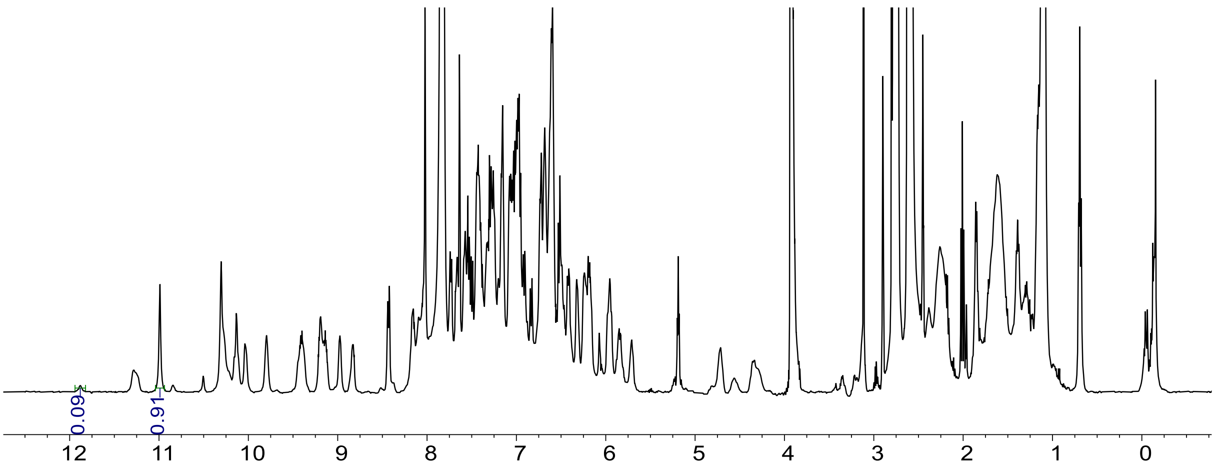

Figure S 10 ^{1}H NMR of compound **6** in (DMF-d_7/H_2O, 9:1, v/v) at 25 °C.

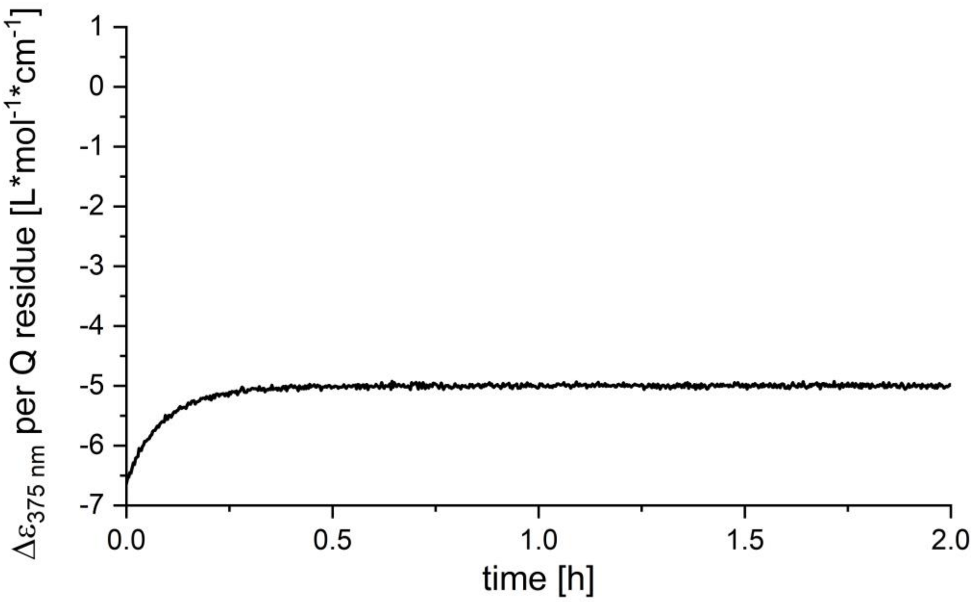

Figure S 11 Time-dependent monitoring of the CD-band at 375 nm of compound **6**, which was dissolved in H_2O and freshly diluted into nine times its volume of DMF, showing enrichment of the foldamer P-helix.

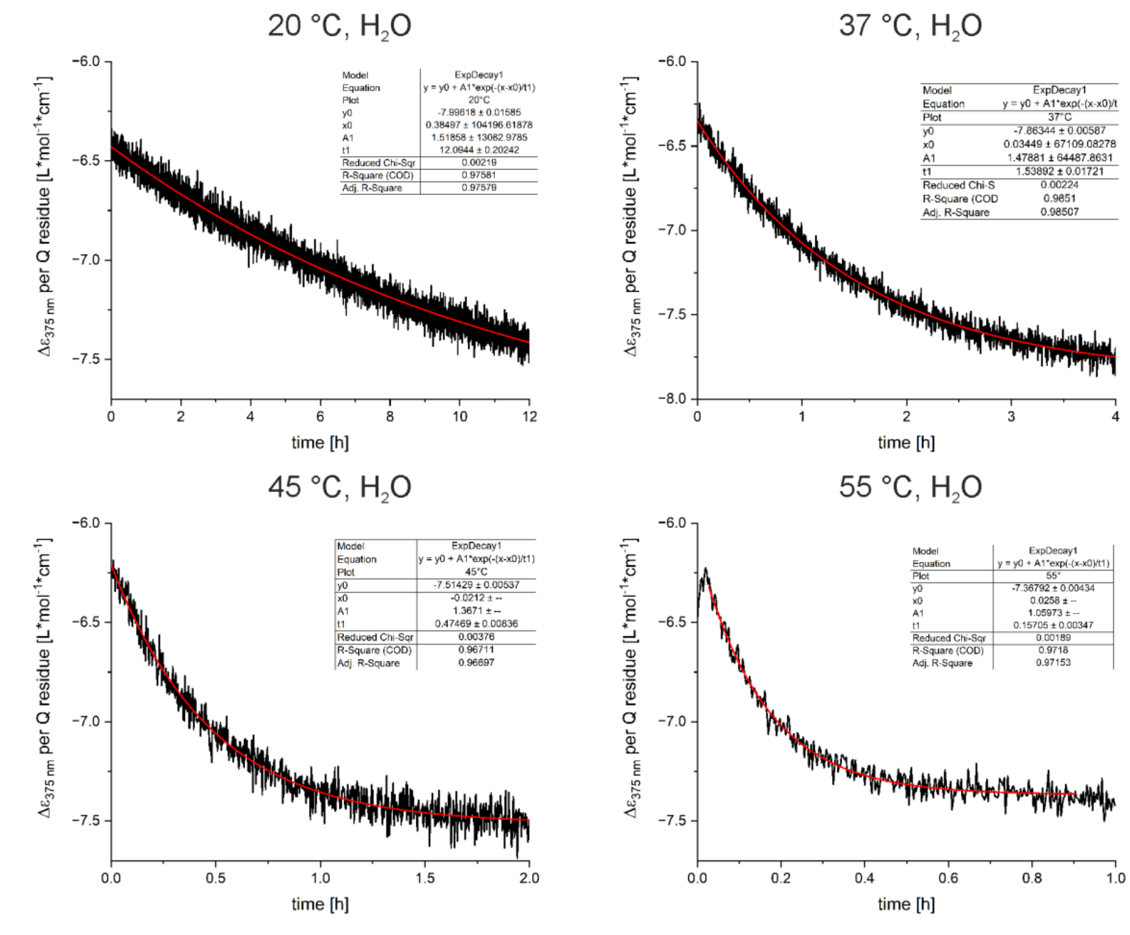

Figure S 12 Helix-handedness enrichment of **6**, showing conversion of excess *P*-helix to *M*-helix (black), and a single-exponential decay fit to the corresponding data (red) in H_2O at different temperatures.

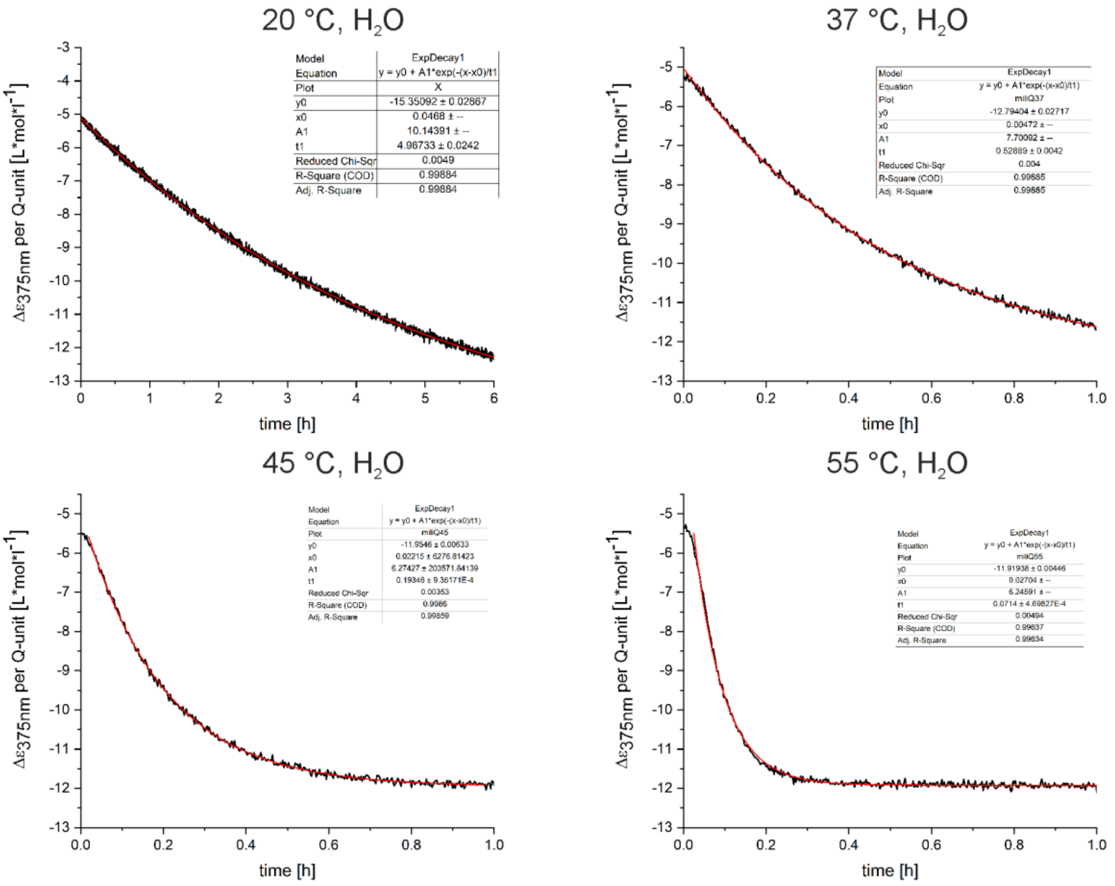

Figure S 13 Helix-handedness enrichment of **5**, showing conversion of excess *P*-helix to *M*-helix (black), and a single-exponential decay fit to the corresponding data (red) in H_2O at different temperatures.

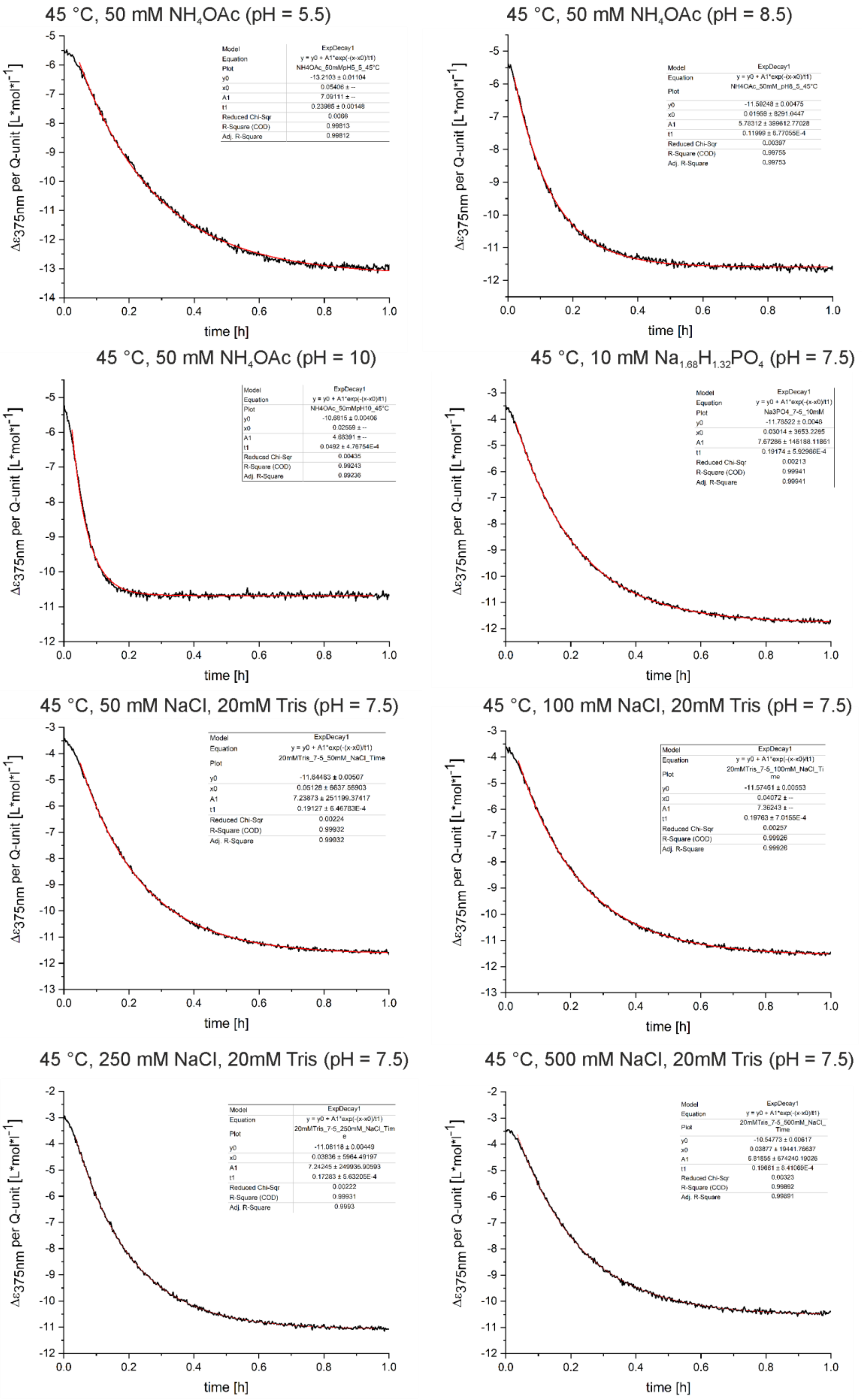

Figure S 14 Helix-handedness enrichment of **5**, showing conversion of excess *P*-helix to *M*-helix (black), and a single-exponential decay fit to the corresponding data (red) in H_2O at different pH values and salt-concentrations.

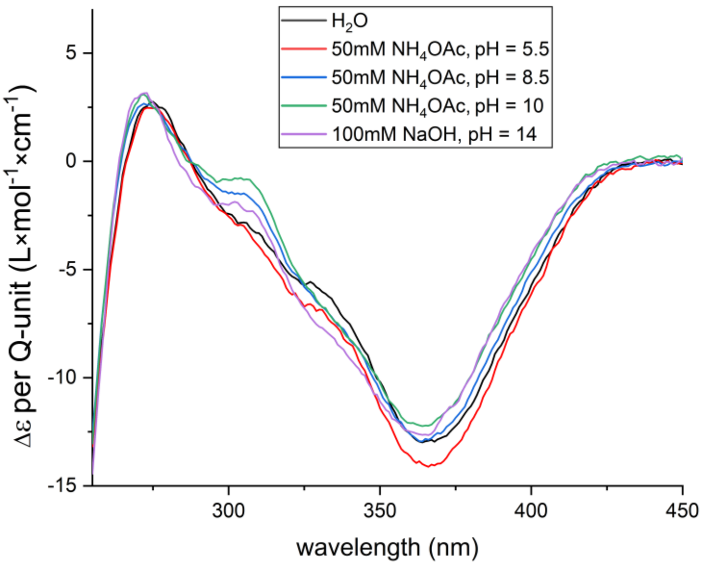

Figure S 15 CD-spectra of **5** at different pH values at 25 °C.

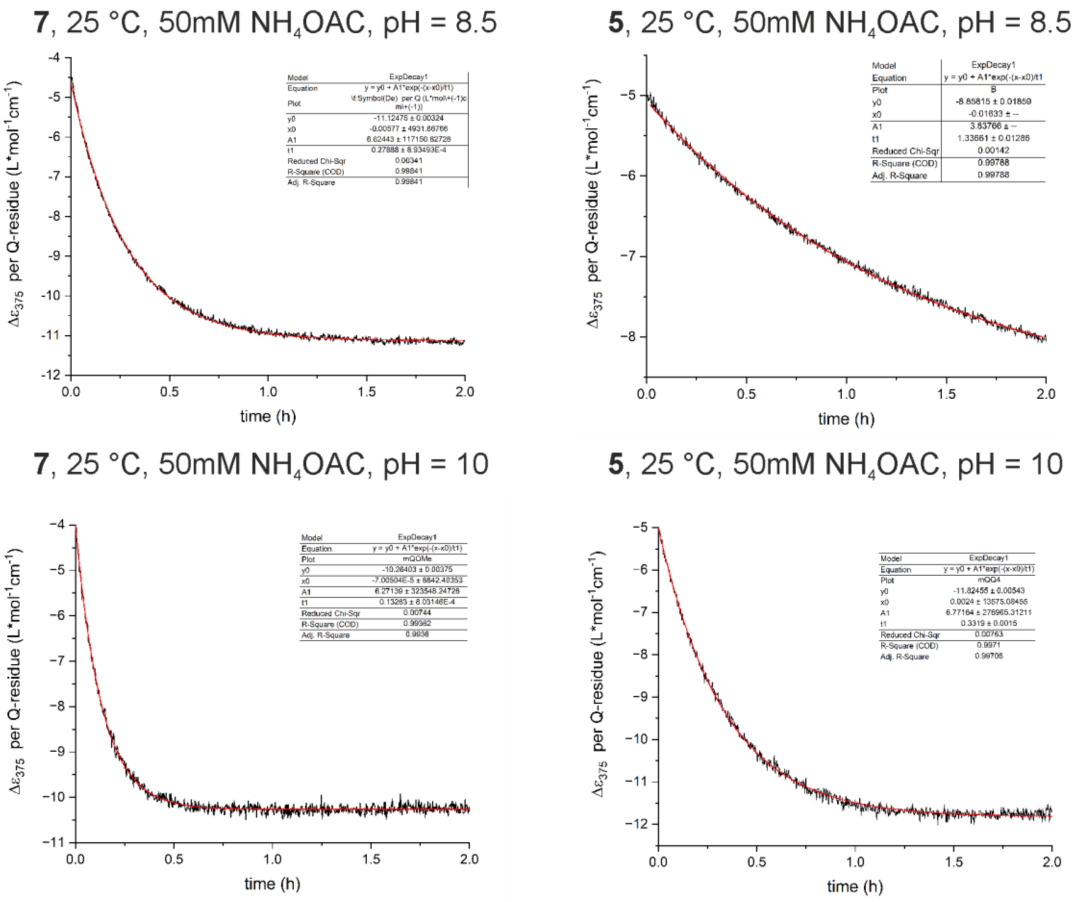

Figure S 16 Helix-handedness enrichment of **5** and **7**, showing conversion of excess *P*-helix to *M*-helix (black), and a single-exponential decay fit to the corresponding data (red) in H_2O at different pH values.

2 Materials and Methods

2.1.1 Materials

Reagents were used as purchased from commercial sources without further purification. Column chromatography purifications were performed on silica gel (230-400 mesh, 40-63 µm, Merck). Thin-layer chromatography was performed on silica gel plates (60-F254, Merck). Reactions requiring anhydrous conditions were performed under nitrogen with commercial anhydrous solvents unless stated otherwise. Anhydrous THF for solid-phase synthesis was dispensed from a *MBRAUN SPS-800* solvent purification system using alumina columns for drying. $CHCl_3$ was freshly distilled over CaH_2 under N_2-atmosphere. Ultrapure water was obtained via a Stakpure OmniaPure-T UV-TOC ultrapure water system.

2.1.2 Small molecule nuclear magnetic resonance spectroscopy and mass spectrometry

NMR spectra were recorded on AVANCE NEO NMR spectrometer 500 MHz (Bruker BioSpin) with CryoProbe™ Prodigy and a BCU II. $CDCl_3$ (δ_H: 7.26, δ_C: 77.0), DMSO-d_6 (δ_H: 2.50, δ_C: 39.4) were used as solvents and their residual solvent signals were used as internal standards.[1] The derived data signals are stated with chemical shift in ppm, their multiplicity (s = singlet, bs = broad singlet, d = doublet, t = triplet, q = quadruplet, m = multiplet, or a combination of these), their coupling constant in Hz and their integrated values. Small molecule mass spectra were recorded on a micrOTOF II mass spectrometer by Bruker Daltonics and ionized by ESI.

2.1.3 Oligomer NMR spectroscopy

One- and two-dimensional spectra were recorded at 25 °C in NH_4HCO_3 (50 mM, H_2O/D_2O (9:1 v/v)) at pH 8 or adjusted to pH 12.5 with NaOH on an Avance III HD 500 MHz Bruker BioSpin spectrometer equipped with a CryoProbe^{TM} Prodigy. For small molecule analysis and measurements with variable temperature (VT) control, an AVANCE NEO NMR spectrometer 500 MHz (Bruker BioSpin) with CryoProbe™ Prodigy and a BCU II were used. Chemical shifts are reported in ppm and are referenced against an internal standard. Data processing was performed with MestReNova NMR processing software (v.12.0.0) from Mestrelab Research. 2D homonuclear correlation spectroscopy (COSY) spectra were recorded with a phase-sensitive pulse sequence with water suppression employing a Watergate pulse scheme from the Bruker pulse program library (cosygpphwpp5). Data acquisition was performed with 1K (F2) x 256 (F1) data points. The recycling delay was 1.0 s and 64 transients per increment were applied at a sweep width of 8 kHz in both dimensions resulting in an acquisition time of 0.1204 s. Automatic phase correction as well as baseline correction was applied in both dimensions.

2.1.4 RP-HPLC chromatography and LC-MS

Analytical RP-HPLC analysis and semi-preparative purifications were performed with 4 different buffer systems. TEAA- and NH_4OAc buffer systems were adjusted to the given pH-values by adding NEt_3 for TEAA and aqueous NH_3 (1 M) NH_4OAc on a Mettler ToledoTM SevenCompact pH meter.

Analytical RP-HPLC analysis and semi-preparative purifications (eg. Compound **5a** and **6a**) were performed with a TFA buffered system 0.1% TFA in ultra-pure water (referred to as mobile phase A) and 0.1% TFA in HPLC-grade acetonitrile (referred to as mobile phase B) were used on a Thermo Fisher

Scientific Ultimate 3000 HPLC system equipped with Macherey-Nagel Nucleodur C18 columns (4.6 × 100 mm, 5 µm) and a flowrate of 1 mL/min for analysis and a Waters system equipped with a 2545 Quaternary Gradient Module and a XBridge® Prep C8 OBD™ column (19 × 150 mm, 5 µm) and a flowrate of 25 mL/min for purifications. LC-MS analysis of these compounds were performed on a a Thermo Fisher Scientific Ultimate 3000 HPLC system equipped with Macherey-Nagel Nucleodur C18 gravity (2× 50 mm, 1.8 µm) and 0.18% formic acid and 0.02% TFA in ultra-pure water (referred to as mobile phase A) and 0.18% formic acid and 0.02% TFA in LCMS-grade acetonitrile (referred to as mobile phase B) and a flowrate of 0.33 mL/min. Analytical RP-HPLC analysis and semi-preparative purifications in basic conditions (e.g. compound **5** and **6**) were performed on a Thermo Fisher Scientific Ultimate 3000 HPLC system using Macherey-Nagel Nucleodur C18 columns (4.6 × 150 mm, 5 µm) with a triethylammonium acetate buffer system with a flowrate of 1 mL/min. The mobile phase was composed of 12.5 mM TEAA in water at pH 8.5 (A) and 12.5 mM TEAA in water: acetonitrile mixture (1:2, v/v) at pH = 8.5 (B). Purifications in basic conditions were done on a Thermo Fisher Scientific Ultimate 3000 HPLC system equipped with a a Kinetex C18 EVO column (10 x 100 mm, 5 µm) and a flowrate of 5 mL/min. LCMS analysis of these compounds were conducted on a Thermo Fisher Scientific Ultimate 3000 HPLC system with an NH_4OAc buffer system consisting of 12.5 mM NH_4OAc dissolved in ultra-pure water and adjusted to pH = 8.5 with aqueous ammonia (referred to as mobile phase A) and LCMS-grade acetonitrile (referred to as mobile phase B) on a Kinetex C18 EVO column (2.1 x 50 mm, 1.8 µm) column and a flowrate of 0.33 mL/min. In all cases, elution was monitored by UV detection at 254 and 300 nm with a diode array detector. TEAA- and NH_4OAc buffer systems were adjusted to the given pH-values by adding NEt_3 for TEAA and aqueous NH_3 (1 M) NH_4OAc on a Mettler ToledoTM SevenCompact pH meter. Formic acid/TFA and TFA buffer systems were not adjusted. For LCMS analysis, the LC system was coupled to a micrOTOF II mass spectrometer by Bruker Daltonics and molecules were ionized by ESI.

2.1.5 Circular dichroism (CD) and UV-Vis absorption spectroscopy

CD-spectra were recorded on a Jasco J-1500 CD spectrometer. Full CD-spectra were recorded from 450 to 250 nm with a continuous scanning rate of 50 nm/min, a Digital Integration Time (D.I.T.) of 0.5 seconds and a bandwidth of 1.00 nm. The data shown are the mean of two measurements and were smoothed using a Savitzky-Golay filter with a polynomal order of 3. Time-course measurements were recorded at 375 nm with a D.I.T of 2 seconds and a data pitch of 10.0 seconds with a Peltier element for temperature control. UV-Vis spectra were measured on a Jasco V-750 spectrophotometer with a peltier element for temperature control. Spectra were recorded from 500 to 250 nm, a bandwidth of 2.00 nm, a continuous scanning mode with a scanning speed of 400 nm/min and a UV-Vis response of 0.06 s. All spectra were recorded in 2 mm quartz glass cuvettes at a concentration range of 30-40 µM for CD spectra and 50 µM for UV spectra. Baseline correction with the respective solvent or buffer used was implemented. DNA mimic foldamers are readily soluble in water. Concentrations were determined by UV-absorbance using an average ε value at 375 nm per monomer of 2506 $Lmol^{-1}cm^{-1}$ for $^{m}QQ^{4}$ sequences and 2140 $Lmol^{-1}cm^{-1}$ for $^{m}QQ^{5}$ sequences.[2]

2.2 Foldamer building blocks

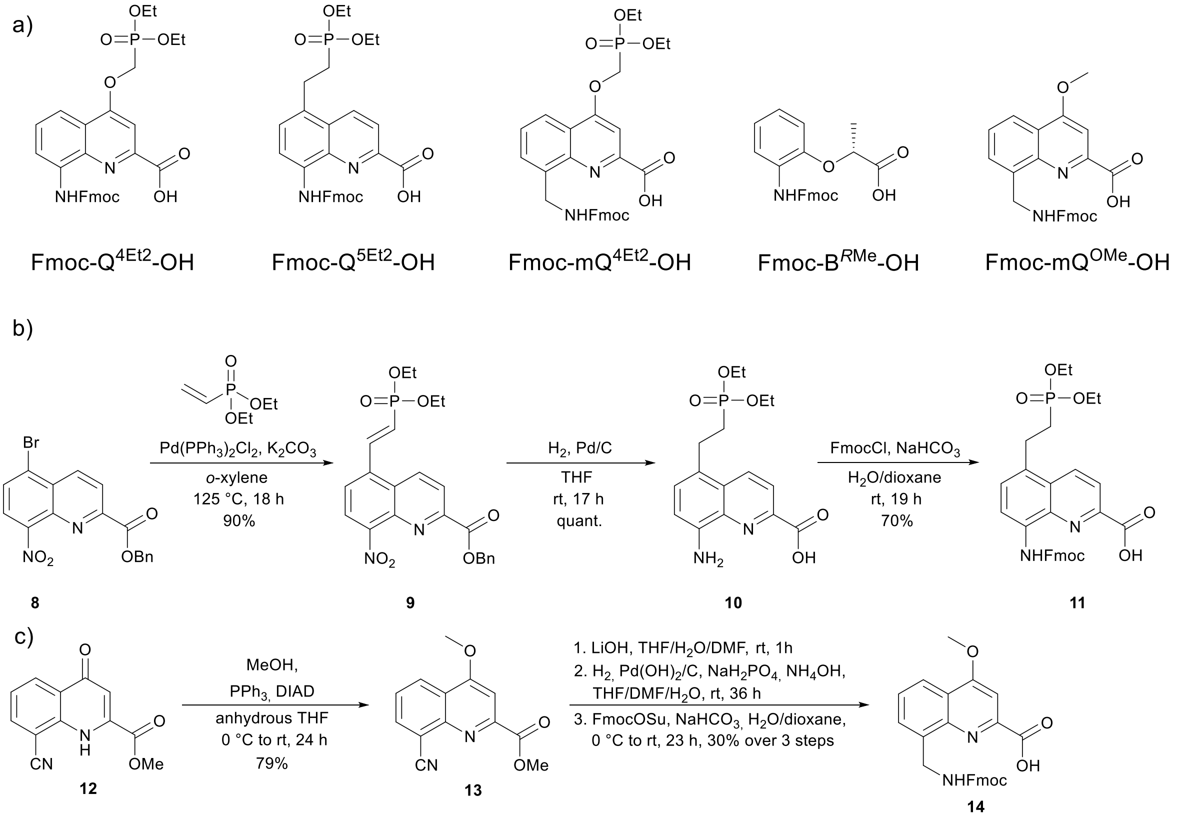

Figure S 17 a) Respective diethyl phosphonate Fmoc-protected monomers for solid-phase synthesis. The synthesis of monomers Fmoc-Q^{4Et2}-OH, Fmoc-mQ^{4Et2}-OH and B^{RMe} are described elsewhere.[3,4] b) Synthetic route towards monomer Fmoc-Q^{5Et2}-COOH **11** from described intermediate **8**, that was synthesized according to published procedures.[5] c) Synthesis of Fmoc-mQ^{OMe}-COOH monomer **14** from described intermediate **12**, that was synthesized according to published protocols.[6]

2.3 Synthetic procedures

Synthesis of 9 (Synthesis of precursor 8 is described here.[4])

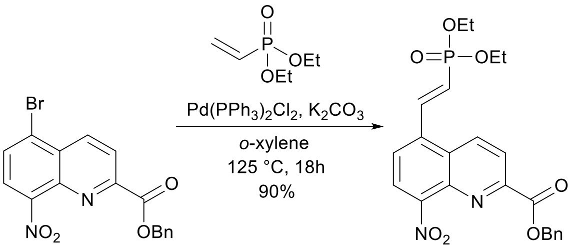

In a dry flask, freshly vacuum oven-dried (60 °C, overnight) **8** (1.50 g, 3.87 mmol, 1.0 eq.) and oven-dried (120 °C) K_2CO_3 (535 mg, 3.87 mmol, 1.0 eq.) were suspended in anhydrous o-xylene (15 mL) under N_2-atmosphere. To this, diethyl vinyl phosphonate (763 mg, 714 µL, 4.65 mmol, 1.2 eq.) and $Pd(PPh_3)_2Cl_2$ (81.6 mg, 116 µmol, 0.03 eq.) were added and the solution was stirred at 125 °C for 18 h under N_2-atmosphere until the HPLC analysis of an aliquot showed full conversion of the starting material. The black solution was diluted with EtOAc (200 mL) and washed with citric acid (2x, 150 mL,

5% w/v). The combined aqueous phases were extracted with EtOAc (2 x 100 mL) and the combined organic phases were washed with brine (200 mL), dried over Na_2SO_4, filtered and the solvent was removed *in vacuo* and dried at a high-vacuum line. The crude product was purified by column chromatography (dry load, 60% → 100% EtOAc in cyclohexane, starting R_F = 0.2) to give the title compound (1.64 g, 3.50 mmol, 90%) as yellow solid.

^{1}H NMR (500 MHz, $CDCl_3$) δ (ppm) = 8.71 (d, *J* = 8.9 Hz, 1H), 8.35 (d, *J* = 8.9 Hz, 1H), 8.17 (dd, *J* = 22.1, 17.3 Hz, 1H), 8.11 (d, *J* = 7.9 Hz, 1H), 7.90 (d, *J* = 7.9 Hz, 1H), 6.52 (t, *J* = 17.0 Hz, 1H), 5.50 (s, 2H), 4.27 – 4.16 (m, 4H), 1.40 (t, *J* = 7.1 Hz, 6H).

^{13}C NMR (126 MHz, $CDCl_3$) δ (ppm) = 164.30, 150.35, 149.28, 141.77 (d, *J* = 7.3 Hz), 139.29, 137.10 (d, *J* = 23.6 Hz), 135.48, 133.62, 128.82, 128.57, 128.33, 127.89, 125.54 (d, *J* = 2.0 Hz), 124.23, 123.29 (d, *J* = 188.9 Hz),123.15, 68.00, 62.53 (d, *J* = 5.6 Hz), 16.63 (d, *J* = 6.1 Hz).

^{31}P NMR (202 MHz, $CDCl_3$) δ (ppm) = 16.11.

HRMS: (ESI^+) *m/z* calc. for $C_{23}H_{24}N_2O_7P^+$: 471.1316 $[M+H]^+$; found: 471.1312.

Synthesis of 10

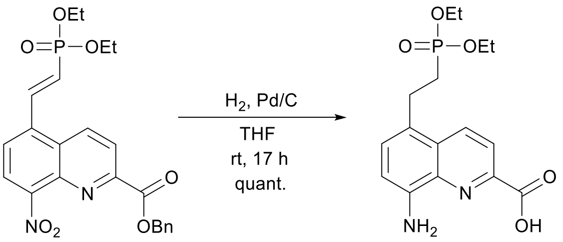

Compound **9** (1.93 g, 4.10 mmol, 1 eq.) was suspended in THF (contained no stabilizer and was freshly filtered over an alumina column from a solvent dispenser system, 20 mL, peroxide containing THF might undergo addition during hydrogenation) under N_2- atmosphere and the solvent was degassed for 10 min by bubbling N_2-gas through the solution while sonicating. Pd/C (200 mg) was added and the solvent was degassed for an additional 5 min. The reaction mixture was put under H_2-atmosphere and vigorously stirred at rt for 17 h. The crude product was filtered over celite and washed with THF (500 mL). The solvent was removed *in vacuo*, co-evaporated with DCM, and dried at the high-vacuum line to give the title compound (1.44 g, 4.10 mmol, quant.) as a yellow solid that was used in the next step without further analysis

Synthesis of 11

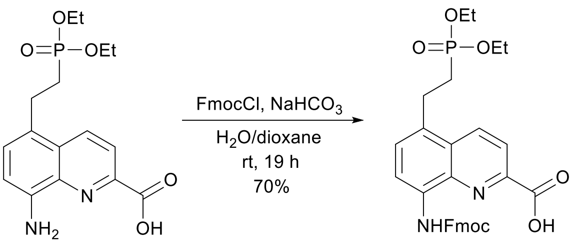

Amino acid **10** (1.44 g, 3.92 mmol, 1.0 eq., 96% purity by HPLC) was suspended in dioxane (80 mL) and $NaHCO_3$ (6.92 g, 82.4 mmol, 21 eq.) dissolved in water (80 mL) was added. The suspension was cooled to 0 °C and FmocCl (4.71 g, 4.71 mmol, 1.2 eq.) dissolved in dioxane (150 mL) was added dropwise over 1 h at 0 °C. The reaction mixture was stirred at 0°C for 2 h and then stirred at rt for 19 h. The solvents were removed *in vacuo* and the resulting solid was suspended in water (150 mL). The suspension was acidified to pH = 3 with a KHSO4-solution (saturated), extracted with DCM (3 x 200 mL) and dried over Na_2SO_4. After filtration, the solvent was removed *in vacuo*, co-evaporated with DCM, and the resulting solid was dried at the high-vacuum line to remove residual dioxane. The crude product was precipitated from MeCN (20 mL), sonicated shortly to allow full precipitation, filtered, washed with cold MeCN (−14°C) and lyophilized to give the title compound (1.57 g, 2.74 mmol, 70%) as a yellow solid in a yield over 2 steps.

^{1}H NMR (500 MHz, DMSO-d_6) δ (ppm) = 13.59 (s, 1H), 10.40 (s, 1H), 8.65 (d, J = 8.8 Hz, 1H), 8.25 (d, *J* = 8.7 Hz, 1H), 7.93 (d, *J* = 7.5 Hz, 2H), 7.78 (d, *J* = 7.4 Hz, 2H), 7.57 (d, *J* = 8.1 Hz, 1H), 7.43 (t, *J* = 7.4 Hz, 2H), 7.36 (t, *J* = 7.5 Hz, 2H), 4.61 (d, *J* = 6.8 Hz, 2H), 4.44 (t, *J* = 6.8 Hz, 1H), 3.22 (tt, *J* = 11.4, 6.8 Hz, 2H), 2.17 – 2.07 (m, 2H), 1.21 (t, *J* = 7.0 Hz, 6H).

^{13}C NMR (126 MHz, DMSO-d_6) δ (ppm) = 165.35, 153.48, 145.07, 143.70, 140.80, 137.03, 134.70, 134.40, 130.58 (d, *J* = 15.5 Hz), 128.93, 127.77, 127.42, 127.21, 125.14, 120.63, 120.24, 115.90, 66.34, 61.04 (d, *J* = 6.2 Hz), 46.60, 25.62 (d, *J* = 137.0 Hz), 24.05 (d, *J* = 4.0 Hz), 16.26 (d, *J* = 5.7 Hz).

^{31}P NMR (202 MHz, DMSO-d_6) δ (ppm) = 30.62.

HRMS: (ESI^+) *m/z* calc. for $C_{31}H_{32}N_2O_7P^+$: 575.1942.1472 $[M+H]^+$; found: 575.1937.

Synthesis of 13 (Synthesis of precursor 12 is described here.[5])

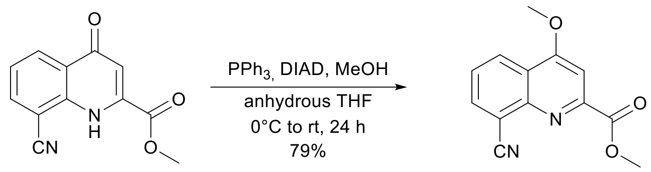

Synthesis of compound **12** is described here.[5] Compound **12** (8.00 g, 35.1 mmol, 1.0 eq.), PPh_3 (11.95 g, 45.6 mmol, 1.3 eq.) and MeOH (1.68 g, 2.13 mL, 52.6 mmol, 1.5 eq.) were suspended in anhydrous THF (70 ml) under N_2-atmosphere and cooled to 0 °C. DIAD (9.22 g, 8.95 mL, 45.6 mmol, 1.3 eq.) was added dropwise over 20 min at 0 °C. The resulting solution was stirred at 0 °C for 1 h, at rt for a further 2 h, and at 50 °C for 25 h. THF was removed *in vacuo* and the residuals taken up in MeOH and sonicated until full precipitation. The resulting solid was filtered off and dried overnight over under reduced pressure. The crude product was dissolved in $CHCl_3$, and a layer of MeOH was added on top. It was crystallized at –24 °C overnight, filtered and washed with cold MeOH. Crystallization was repeated twice, until the HPLC analysis of an aliquot showed no residual PPh_3O, to yield the title compound **2** (6.67 g, 27.54 mmol, 79%) as an off-white crystalline solid.

^{1}H NMR (500 MHz, $CDCl_3$) δ (ppm) = 8.48 (dd, *J* = 8.5, 1.4 Hz, 1H), 8.17 (dd, *J* = 7.2, 1.4 Hz, 1H), 7.69 (s, 1H), 7.66 (dd, *J* = 8.5, 7.2 Hz, 1H), 4.17 (s, 3H), 4.08 (s, 3H).

^{13}C NMR (126 MHz, $CDCl_3$) δ (ppm) = 165.94, 163.86, 151.31, 147.86, 136.85, 127.25, 126.76, 122.51, 117.01, 113.78, 101.87, 56.74, 53.62.

HRMS: (ESI^+) m/z calc. for $C_{13}H_{11}N_2O_3^+$: 243.0764 $[M+H]^+$; found: 243.0765.

Synthesis of 14

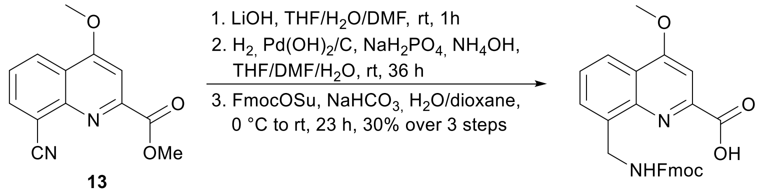

13 **14**

In a 2 liter round bottom flask **13** (5.5 g, 22.7 mmol, 1.0 eq.) was dissolved in 850 ml THF/DMF (90:10 v/v). LiOH (816 mg, 34.1 mmol, 1.5 eq.) dissolved in 310 ml H_2O was added and stirred at rt for 1 h until TLC showed full consumption of the starting material. $NaH_2PO_4{\cdot}(H_2O)_2$ (35.4 g, 227 mmol, 10 eq.) was added as a solid too the ternary solvent mixture. Then, the solvent was degassed for 15 min by bubbling N_2-gas through the solution while sonicating. Afterwards, $Pd(OH)_2/C$ (542 mg) and NH_4OH (20%, 12.93 mL, 68.13 mmol, 3.0 eq.) were added and the black suspension was further degassed for 15 min. Under vigorous stirring, a H_2-balloon was placed on top of the round bottom flask and the reaction mixture was stirred for 36 h at rt under H_2 atmosphere until no nitrile-containing intermediate product was detectable by HPLC in an aliquot. To the mixture (82% HPLC purity at 300 nm), $NaHCO_3$ (9.53 g, 114 mmol, 5 eq.) dissolved in H_2O (200 mL) was added, and FmocOSu (8.04 g, 23.8 mmol, 1.05 eq.) was added dissolved in THF (200 mL) at 0°C over 1 h, stirred at 0°C for 1 h and 21 h at rt. The

mixture was filtered through a paper filter, washed with THF and then concentrated *in vacuo* until a mostly aqueous suspension remained, which was then acidified with $KHSO_4$ (1.8 M) to pH = 2–3. Water (800 mL) and DCM (600 mL) were added and stirred until the mixture was dissolved. The organic phase was separated, and the aqueous phase was extracted with DCM (3x 500 ml). The combined organic phases were dried over $MgSO_4$ and filtered through a paper filter and the solvent was removed *in vacuo*. The resulting crude oil was purified by column chromatography (silica gel, 13% acetone in DCM + 0.1% AcOH (v/v). The solvent was removed *in vacuo* and the resulting solid was recrystallized from boiling MeCN and then recrystallized from boiling EtOAc to yield the title compound as white solid (2.51 g, 5.52 mmol, 30%).

^{1}H-NMR shows 2 conformers in a ratio of 85:15. Integrals are given with their respective integration. Overlapping NMRs signals are integrated as m and their integration is given. For ^{13}C NMR, only the major species is listed.

^{1}H NMR (500 MHz, DMSO-d$_6$): δ [ppm] = 8.11 (dd, *J* = 7.9, 1.9 Hz, 1H), 7.92 – 7.85 (m, 2.6H), 7.80 (d, *J* = 7.5 Hz, 0.3H), 7.69 (d, *J* = 7.5 Hz, 1.7H), 7.67 – 7.59 (m, 2.7H), 7.58 – 7.54 (m, 0.15H), 7.52 – 7.48 (m, 0.15H), 7.45 – 7.36 (m, 2H), 7.34 – 7.27 (m, 2H), 7.10 (t, *J* = 7.6 Hz, 0.3H), 4.84 (d, *J* = 6.2 Hz, 1.7H), 4.73 (d, *J* = 6.3 Hz, 0.3H), 4.40 (d, *J* = 6.8 Hz, 1.7H), 4.33 (d, *J* = 6.3 Hz, 0.3H), 4.25 (t, *J* = 6.8 Hz, 1H), 4.13 (s, 3H).

^{13}C NMR (126 MHz, DMSO-d$_6$): δ [ppm] = 166.09, 163.17, 156.53, 148.55, 145.18, 143.86, 140.76, 137.84, 128.27, 127.58, 127.42, 127.02, 125.10, 121.34, 120.31, 120.11, 99.90, 65.27, 56.47, 46.83, 40.59.

HRMS: (ESI$^+$) m/z calc. for $C_{27}H_{23}N_2O_5$: 455.1602 [M+H]$^+$; found: 455.1613.

.

2.4 Foldamer synthesis

2.4.1 General solid-phase foldamer synthesis

Solid phase synthesis (SPS) was performed manually under MW-irradiation on a CEM Discover (Liberty Bio) microwave oven using a reaction vessel and an internal fiber optic probe for temperature control as described below, or with a fully automated synthesizer followed by previously reported protocol.[7]

- Bromination of low loading Wang resin

Low loading-(LL) Wang resin (Novabiochem, 100-200 mesh, 1.00 g, 0.37 mmol, 1.0 eq.) was swollen in DMF (6 mL) for 30 min. PPh_3 (970 mg, 3.70 mmol, 10.0 eq.) and CBr_4 (1.23 g, 3.70 mmol, 10 eq.) were quickly added and the suspension was stirred slowly for 20 h at RT. The resin was filtered, washed with DMF (3 × 3 mL) and DCM (3 × 3 mL), and dried by passing N_2 through the resin and stored at 4 °C until usage.

- Loading of the 1st monomer unit & resin loading estimation

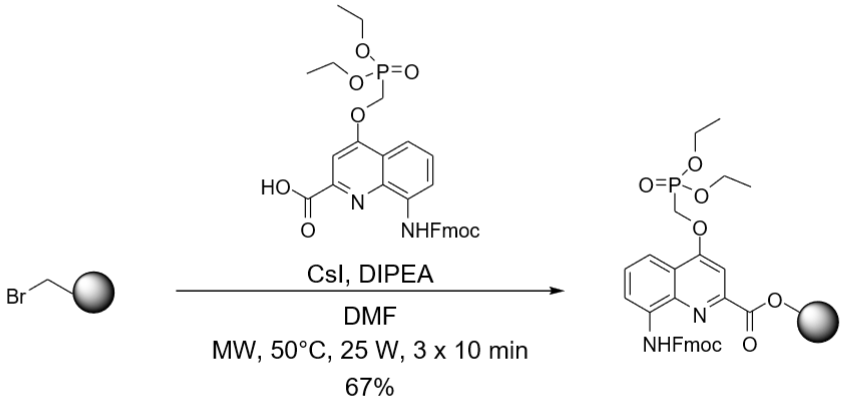

LL-brominated Wang resin (Novabiochem, 100-200 mesh, 100 mg, 37.0 μmol, 1.0 eq.) was swollen in anhydrous DMF (3 mL) for 30 min under N_2. After washing with anhydrous DMF (3 mL), Fmoc-Q_4-COOH (42.5 mg, 74.0 μmol, 2.0 eq.) dissolved in anhydrous DMF (2 mL), CsI (19.2 mg, 74.0 μmol, 2.0 eq.) and freshly distilled DIPEA (9.56 mg, 12.9 μL, 74 μmol, 2.0 eq.) were quickly added and the reaction vessel was placed under microwave irradiation (25 W, ramp to 50°C over 5 min, hold at 50°C for 10 min) while bubbling N_2 through the solution. The resin was washed with anhydrous DMF (3 × 3 mL) and the process was repeated twice. The resin was washed with DMF (3 × 3 mL) and DCM (3 × 3 mL) and dried by passing N_2 through the resin. For resin loading estimation, a solution of 20% piperidine in DMF (v/v, 3 mL) was added to a known mass of the previously dried resin (1–2 mg) and agitated for 5 min. Meanwhile, the absorbance at 290 nm of the piperidine/DMF-solution was measured. After agitation, the absorbance of the solution that contained the resin was measured at 290 nm.

$$Resin\ loading\ \left[\frac{mmol}{g}\right] = \frac{[Abs_{final} - Abs_{initial}]}{[2.00 \times m_{resin}]}$$

- Fmoc deprotection

To the pre-swollen loaded Wang resin (75.0 mg, 0.26 mmol g^{-1}, 19.5 µmol), a 20% solution of piperidine in DMF (3 mL, v/v) was added and the resin was mixed by bubbling N_2-gas through the solution for 3 min. The resin was filtered and washed with DMF (2 x 3 mL) and the deprotection was repeated once for 7 min to give the respective amine NH_2-Q-Wang resin. The resin was filtered, washed with DMF (5 x 3 mL), and washed with anhydrous THF (3 x 3 mL) prior to coupling.

- *In situ* acid chloride activation, coupling, and capping

The NH_2-Wang resin (75.0 mg, 0.26 mmol g^{-1}, 19.5 µmol, 1.0 eq.) was suspended in anhydrous THF (0.9 mmol) and collidine (23.0 µL, 176 µmol, 9.0 eq.) was added. Concurrently, in a glass vial, **Monomer** (35 mg, 58.6 µmol, 3.0 eq.) and PPh_3 (41 mg, 156 µmol, 8 eq.) were mixed and dissolved in anhydrous $CHCl_3$ (0.9 mL). Subsequently, trichloro acetonitrile (18.0 µL, 176 µmol, 9 eq.) was added to the vial, which was quickly shaken, and the mixture was added to the pre-swollen resin. After mixing, the reaction vessel was placed under microwave irradiation (25 W, ramp to 50°C over 5 min, hold at 50°C for 15 min). The resin was filtered off and washed with anhydrous THF (3 x 3 mL). The coupling step was repeated once. The resin was filtered off and washed with anhydrous THF (3 x 3 mL) and DCM (3 x 3 mL) prior to the capping step. The resin was suspended in a 50% solution of Ac_2O in DCM (v/v) and mixed by bubbling N_2-gas through the solution for 10 min. The resin was washed with DCM (2 x 3 mL) and DMF (2 x 3 mL).

- Resin cleavage

The resin-bound foldamer was placed in a syringe equipped with a filter and then suspended in TFA (3 mL). The resin was shaken for 2 h at rt. The resin was then filtered off and washed twice with TFA. The TFA was combined removed *in vacuo* and the resulting oil was precipitated by sonication in cold Et_2O. The precipitate was centrifuged, and the solvent was decanted to give a yellow solid. The decanted Et_2O was concentrated by rotary evaporation and the precipitation was repeated. The combined precipitates were dissolved in water/MeCN and then lyophilized to give the crude protected foldamer as a yellow solid and further purified as a diethyl-phosphonate protected compound.

2.4.2 Removal of the diethyl-phosphonate protecting groups

Purified protected foldamers were dissolved in anhydrous chloroform (1 mL per 10 mg compound) and cooled to 0 °C. TMSBr (0.2 mL per 10 mg compound) was mixed with anhydrous chloroform (0.8 mL per 10 mg compound). The TMSBr-solution was added dropwise over 10 min to the reaction mixture. The reaction mixture was allowed to warm to room temperature and stirred under N_2-atmosphere for 1–3 d until an HPLC- and LCMS aliquot of the reaction mixture showed complete cleavage of all phosphonate-diethyl esters. The reaction mixture was evaporated *in vacuo* (40°C water bath) to give a yellow oil, then co-evaporated with DCM (2x) to give a yellow solid. The solid was suspended in water, basified to pH > 12 with triethylamine and stirred for 30 min. The suspension was filtered through nylon syringe filters (pore size: 0.22 µm) to give a pale-yellow solution that was freeze-dried to give the crude deprotected foldamers as yellow solids.

2.4.3 Cation exchange chromatography

Cation exchange chromatography was performed on Dowex© 50WX4 200-400 (H) resin. The resin was swollen in H_2O the orange solution was decanted. The resin was transferred into a glass column (0.5 cm diameter, height 15–20 cm) and washed with H_2O (gravity flow). It was further washed with two column volumes (CV) of 2 M HCl solution, then H_2O until pH = 6-7 (ca. 5-10 CV), then two CV of 2 M NH_4OAc solution, then 5 CV of H_2O. Purified triethylammonium salts of foldamers were dissolved in water and loaded on the column. The column was closed without flow for 2 h. The compound was eluted with water (20 mL) and lyophilized to give the purified foldamer NH_4^+-salt as yellow solid.

2.4.4 Synthesized foldamers

2.4.4.1 Compound **5a** protected chiral $^{m}QQ^{4}$ based 16mer

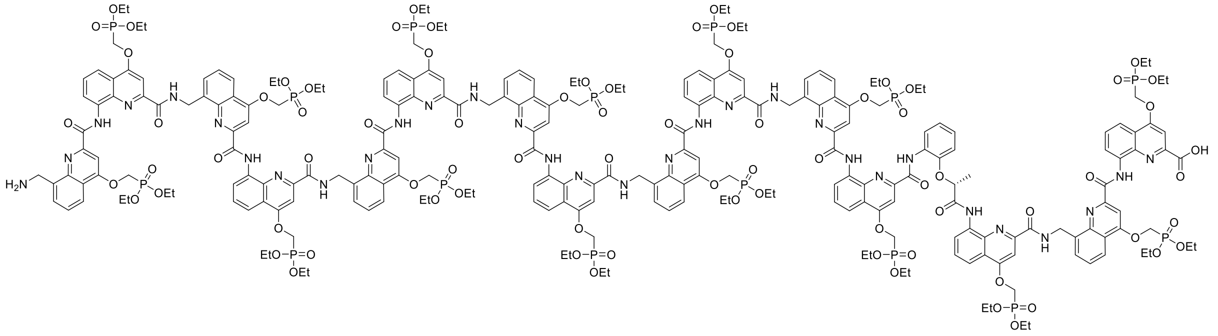

Oligomer **5a** was synthesized on LL-Wang resin (25.0 µmol) according to section 3.3.1. The compound was isolated by semi-preparative RP-HPLC (acidic conditions, linear gradient 33-48% B in A, column XBridge® Prep C8 OBD™) to give the title compound (59.0 mg, 11.1 µmol, 44%) as a yellow solid.
(ESI^+) *m/z* calc. for $C_{241}H_{283}N_{31}O_{78}P_{15}$: 1774.5060 $[M+3H]^{3+}$; found: 1774.5610.

2.4.4.2 Compound **5** deprotected chiral $^{m}QQ^{4}$ based 16mer

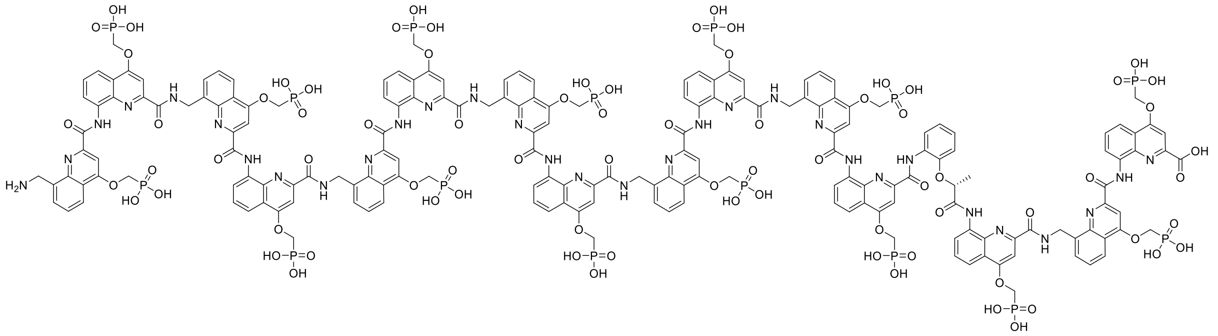

Oligomer **5a** was deprotected based on the protocol given in 3.3.2 (scale 27.0 mg, 5.10 µmol) and purified by semi-preparative RP-HPLC (TEAA buffer system, linear gradient 0-30% B in A (Kinetex C18 EVO column). From the lyophilized powder, the NEt_3 cations were exchanged with NH_4^+ (Section 3.3.3) to give the poly-ammonium salt of compound **5** as yellow solid (18.9 mg, 4.02 µmol, 78%).
(ESI^-) *m/z* calc. for $C_{181}H_{156}N_{31}O_{78}P_{15}$: 1118.8820 $[M-4H]^{4-}$; found: 1118.9263.

2.4.4.3 Compound **6a** protected chiral $^{m}QQ^{5}$ based 16mer

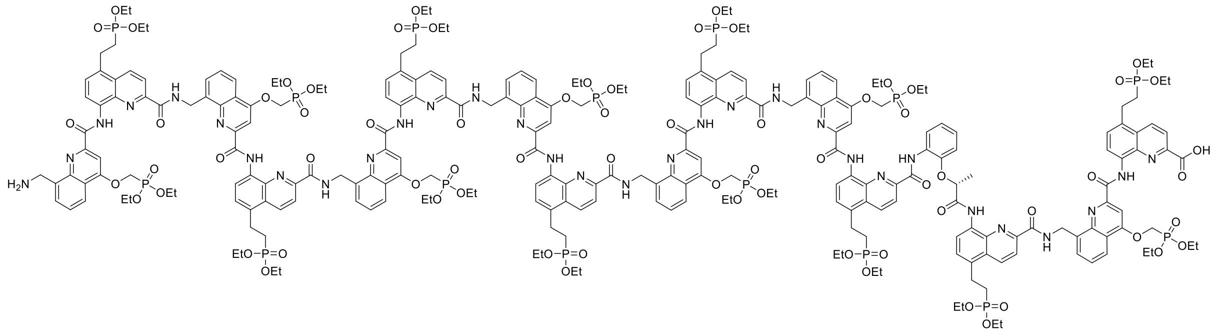

Oligomer **6a** was synthesized on LL-Wang resin (25.0 µmol) according to section 3.3.1. The compound was isolated by semi-preparative RP-HPLC (acidic conditions, linear gradient 35-55% B in A, column XBridge® Prep C8 OBD™) to give the title compound (82.5 mg, 15.5 µmol, 62%) as a yellow solid. (ESI^{+}) *m/z* calc. for $C_{249}H_{299}N_{31}O_{70}P_{15}$: 1769.2279 $[M+3H]^{3+}$; found: 1769.2901.

2.4.4.4 Compound **6** deprotected chiral $^{m}QQ^{5}$ based 16mer

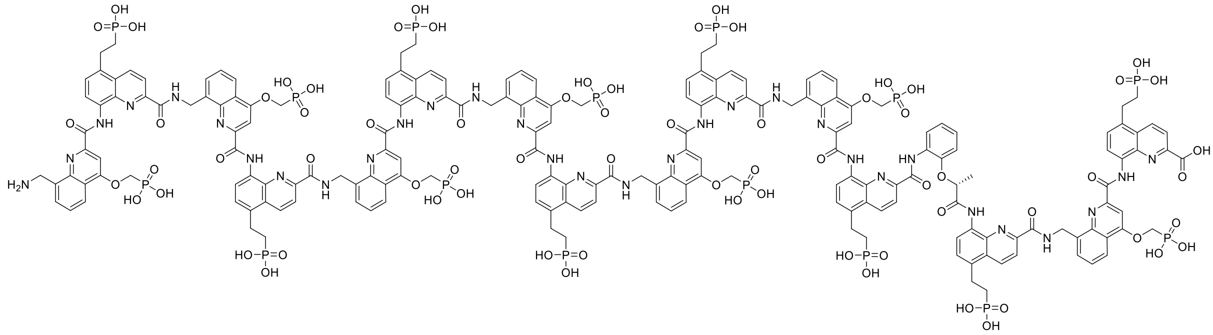

Oligomer **6a** was deprotected based on the protocol given in 3.3.2 (scale 25.0 mg, 4.70 µmol) and purified by semi-preparative RP-HPLC (TEAA buffer system, linear gradient 0-30% B in A (Kinetex C18 EVO column). From the lyophilized powder, the NEt_3 cations were exchanged with NH_4^+ (Section 3.3.3) to give the polyammonium-salt of compound **6** as yellow solid (18.2 mg, 3.87 µmol, 82%). (ESI^{-}) *m/z* calc. for $C_{189}H_{172}N_{31}O_{70}P_{15}$: 1114.9235 $[M-4H]^{4-}$; found: 1114.9294.

2.4.4.5 Compound **7a** protected chiral $^{m}QQ^{4}$-OMe 16mer

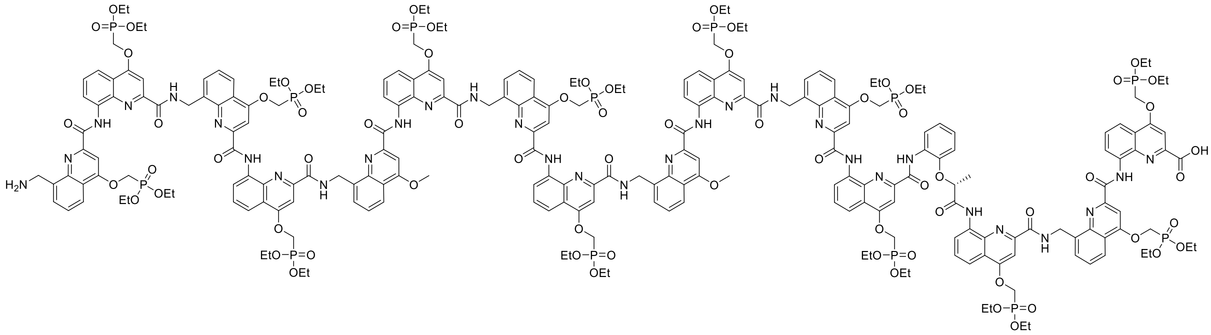

Oligomer **7a** was synthesized on TG-Wang resin (15.0 µmol) according to section 3.3.1. The compound was isolated by semi-preparative RP-HPLC (acidic conditions, linear gradient 35-55% B in A, column XBridge® Prep C8 OBD™) to give the title compound (40 mg, 8.0 µmol, 53%) as a yellow solid.
(ESI^{+}) *m/z* calc. for $C_{233}H_{265}N_{31}O_{72}P_{13}$: 1683.8200 $[M+3H]^{3+}$; found: 1683.8404.

2.4.4.6 Compound **7** deprotected chiral $^{m}QQ^{4}$-OMe 16mer

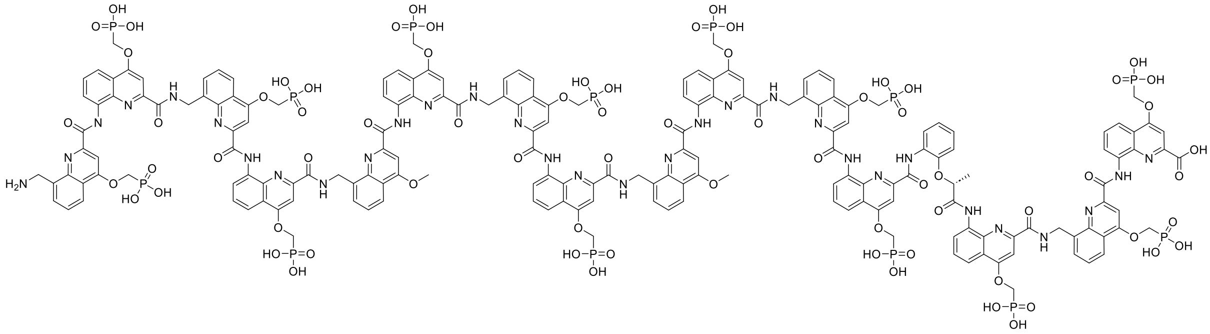

Oligomer **7a** was deprotected based on the protocol given in 3.3.2 (scale 11 mg, 2.20 µmol) and purified by semi-preparative RP-HPLC (TEAA buffer system, linear gradient 0-30% B in A (Kinetex C18 EVO column) and the cation was exchanged to NH_4^{+} (Section 3.3.3) to give polyammonium-salt of compound **7** as yellow solid (5.2 mg, 1.2 µmol, 55%).
(ESI^{-}) *m/z* calc. for $C_{181}H_{155}N_{31}O_{72}P_{13}$: 1078.8988 $[M-4H]^{4-}$; found: 1078.9162.

3 Spectra & Chromatograms

3.1 NMR spectra

3.1.1 Small molecules spectra

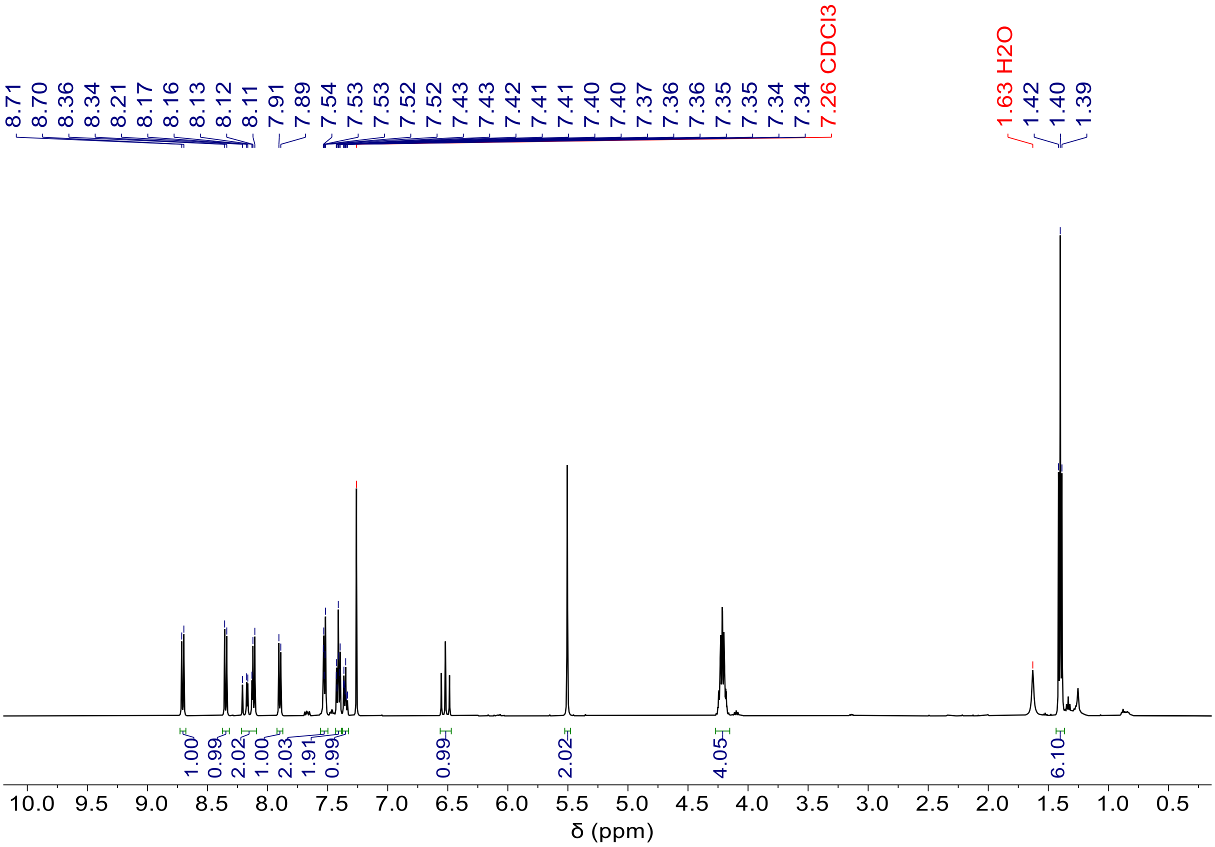

Figure S 18 ^{1}H NMR spectrum of compound **9** in $CDCl_3$.

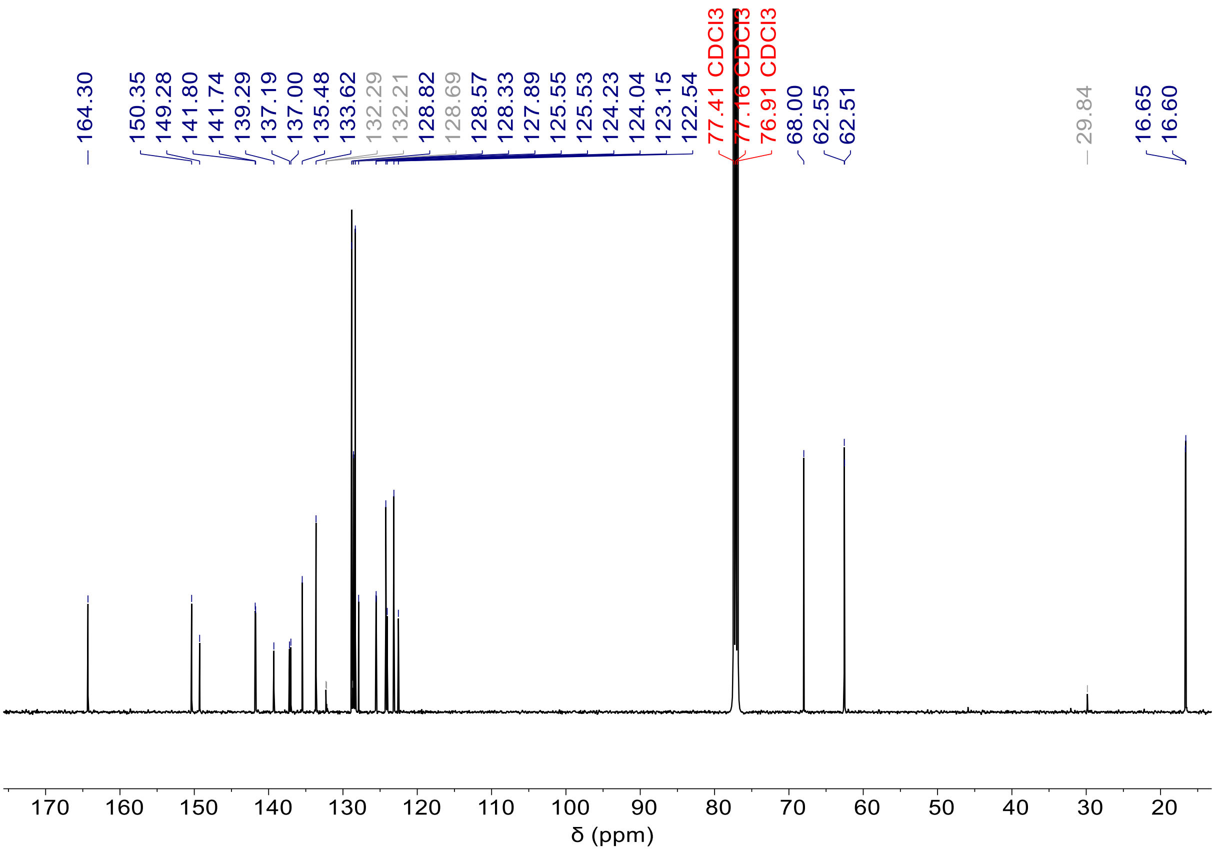

Figure S 19 ^{13}C NMR spectrum of compound **9** in $CDCl_3$.

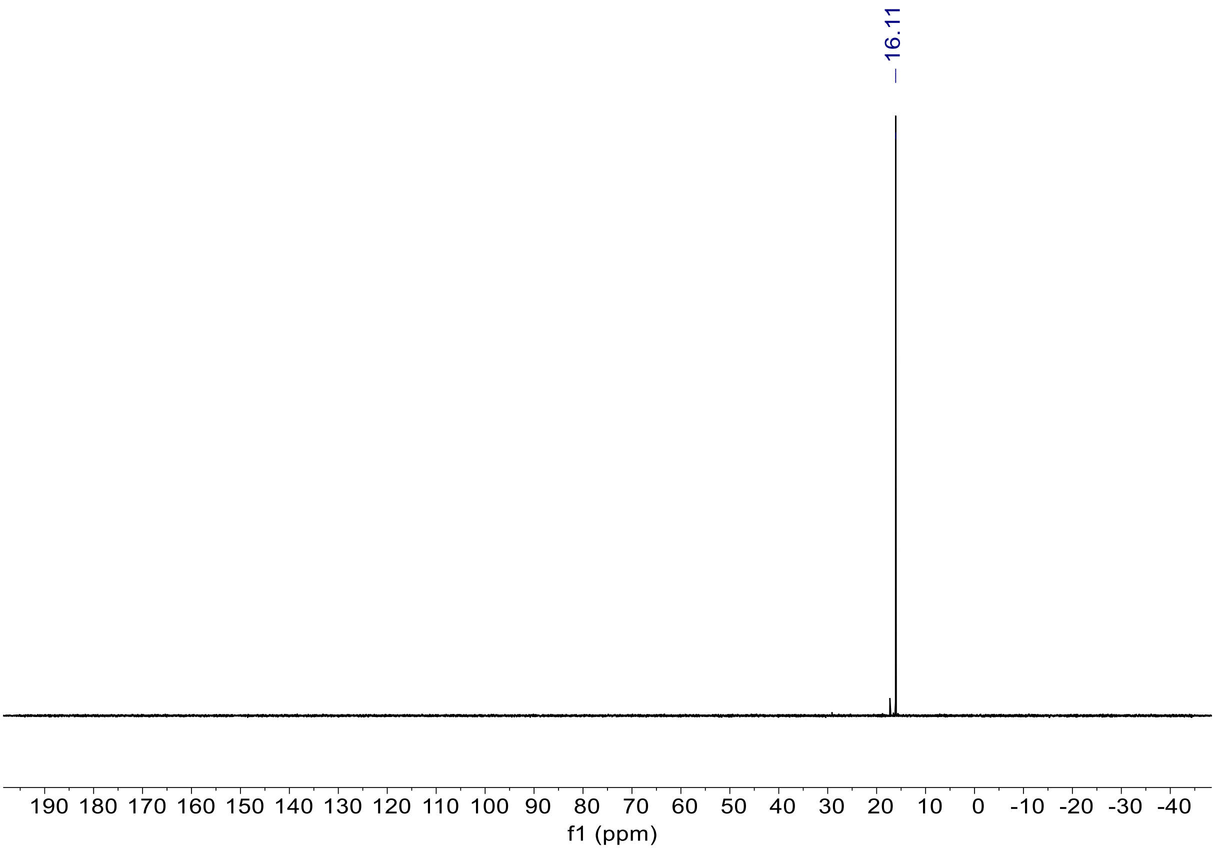

Figure S 20 ^{31}P NMR spectrum of compound **9** in $CDCl_3$.

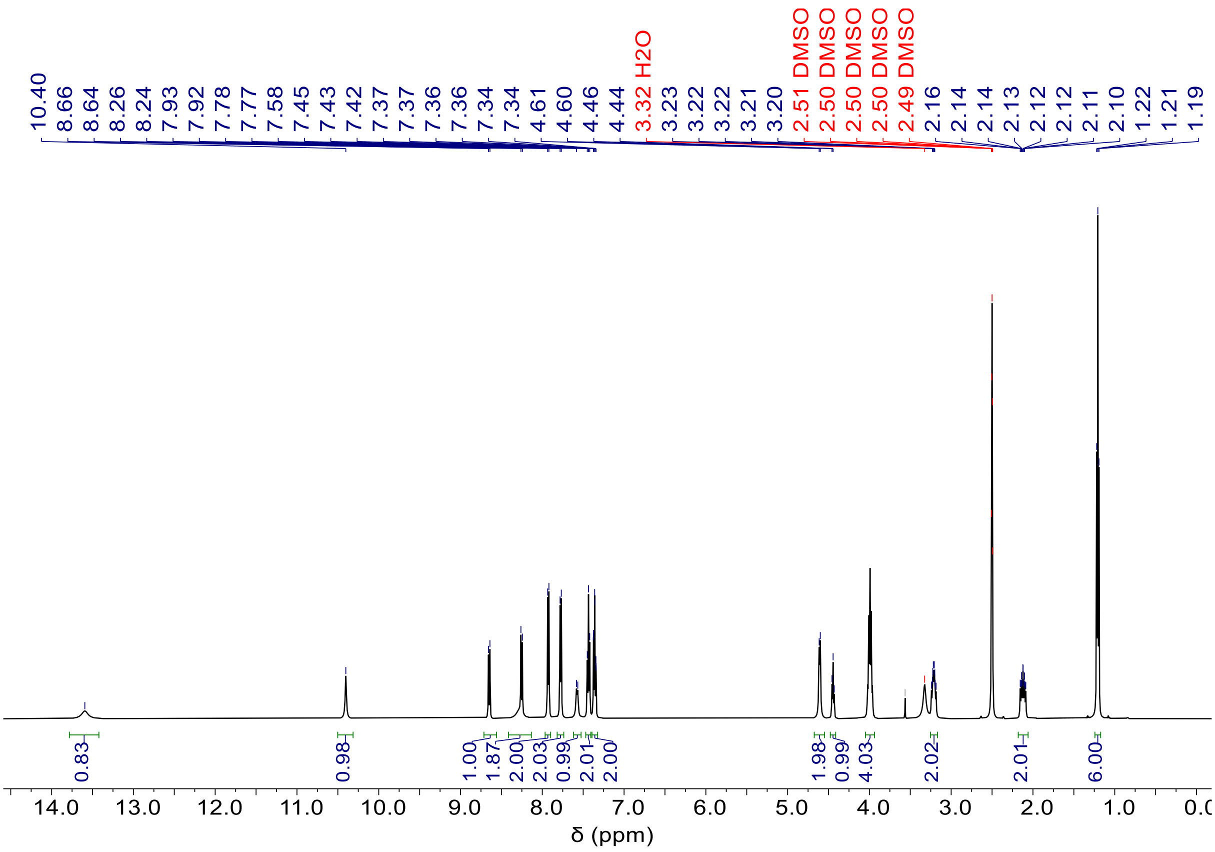

Figure S 21 ^{1}H NMR spectrum of compound **11** in DMSO-d_6.

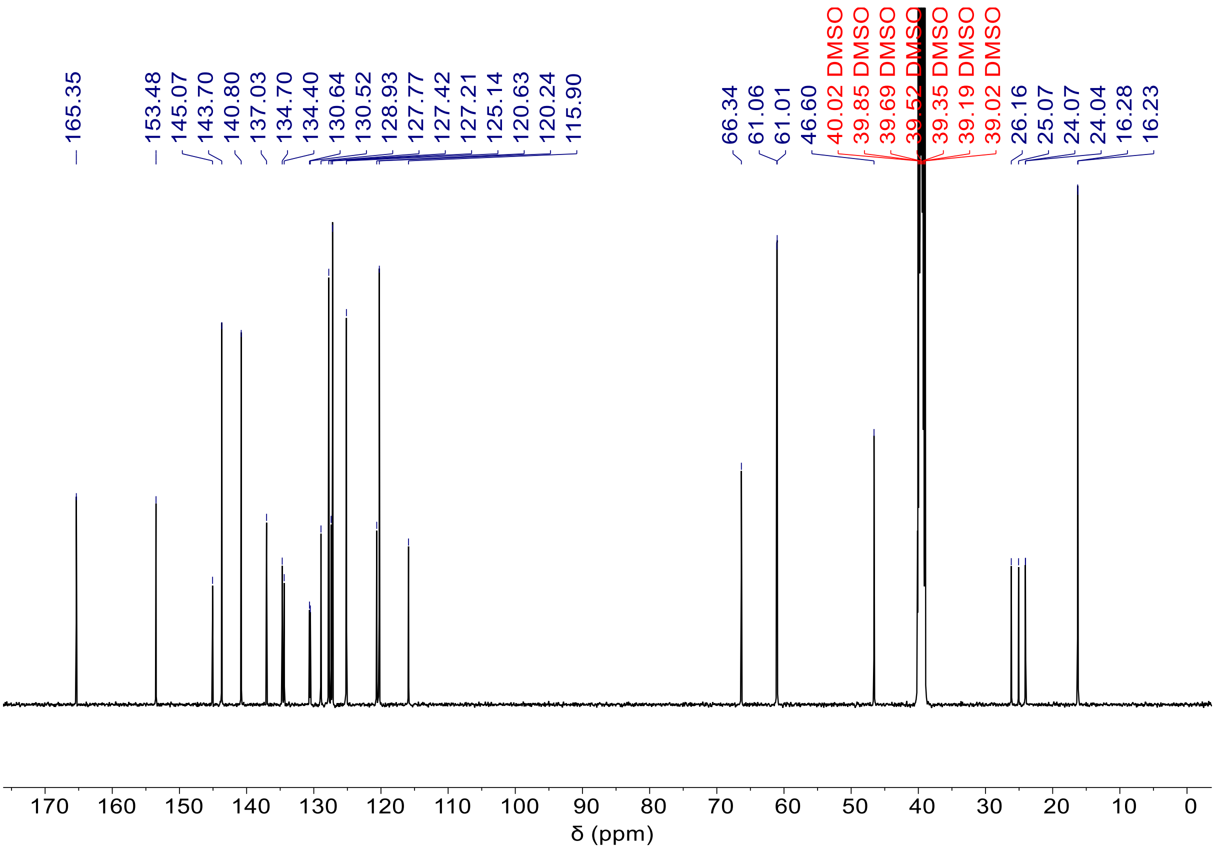

Figure S 22 ^{13}C NMR spectrum of compound **11** in DMSO-d_6.

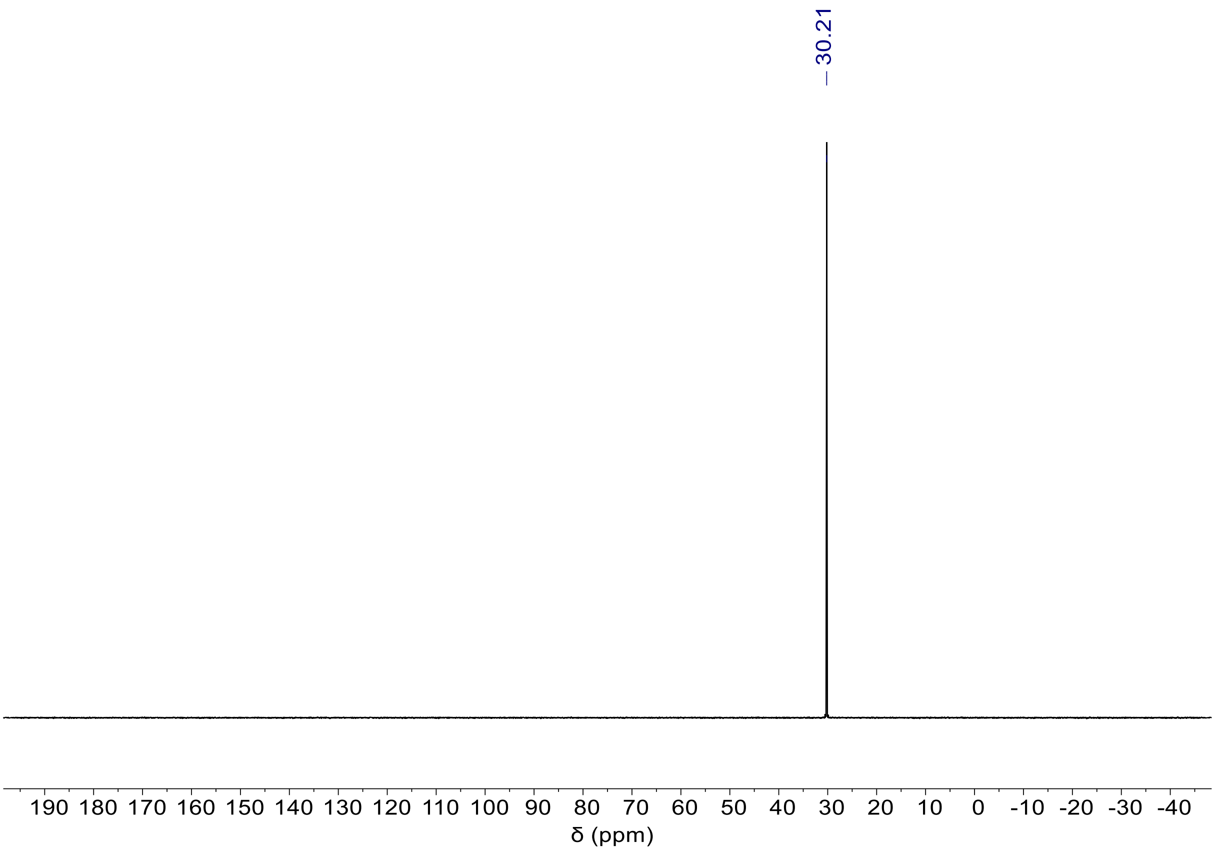

Figure S 23 ^{31}P NMR spectrum of compound **11** in DMSO-d_6.

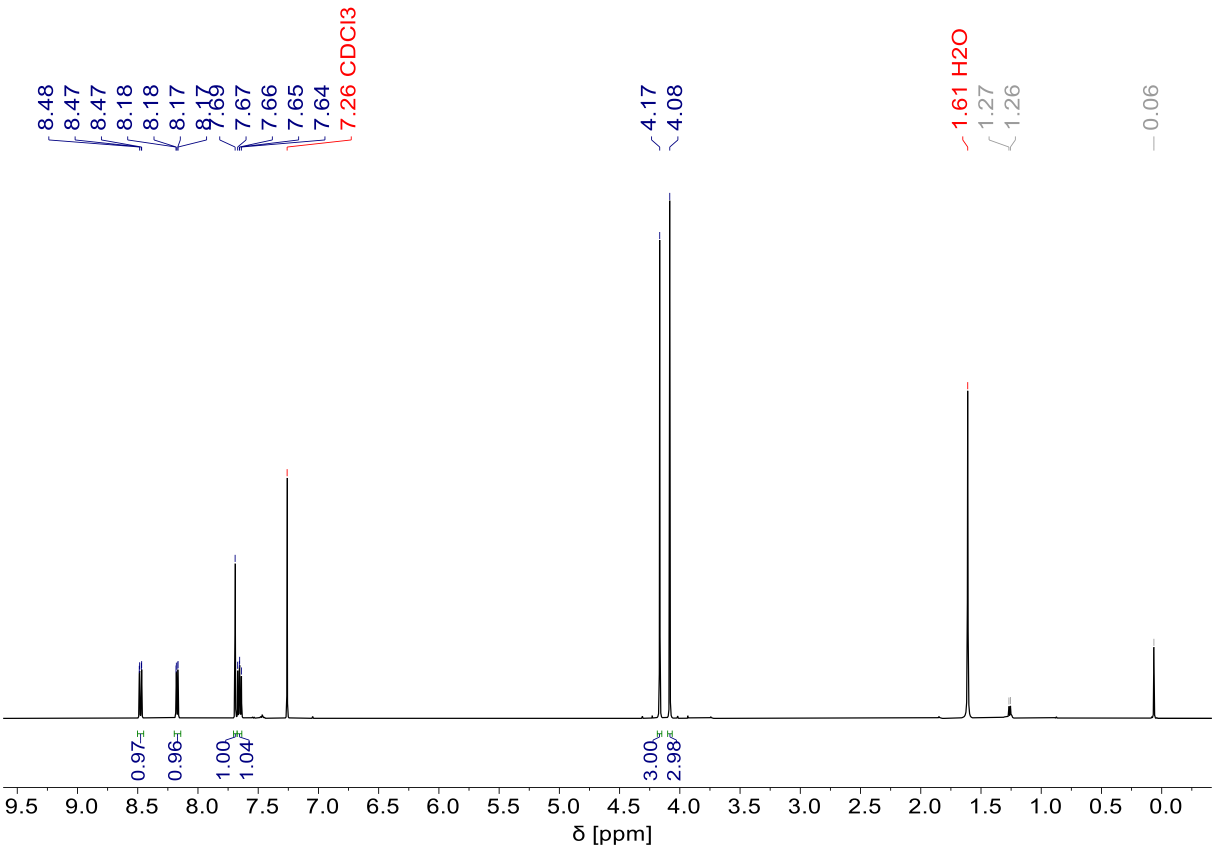

Figure S 24 ^{1}H NMR spectrum of compound **13** in $CDCl_3$.

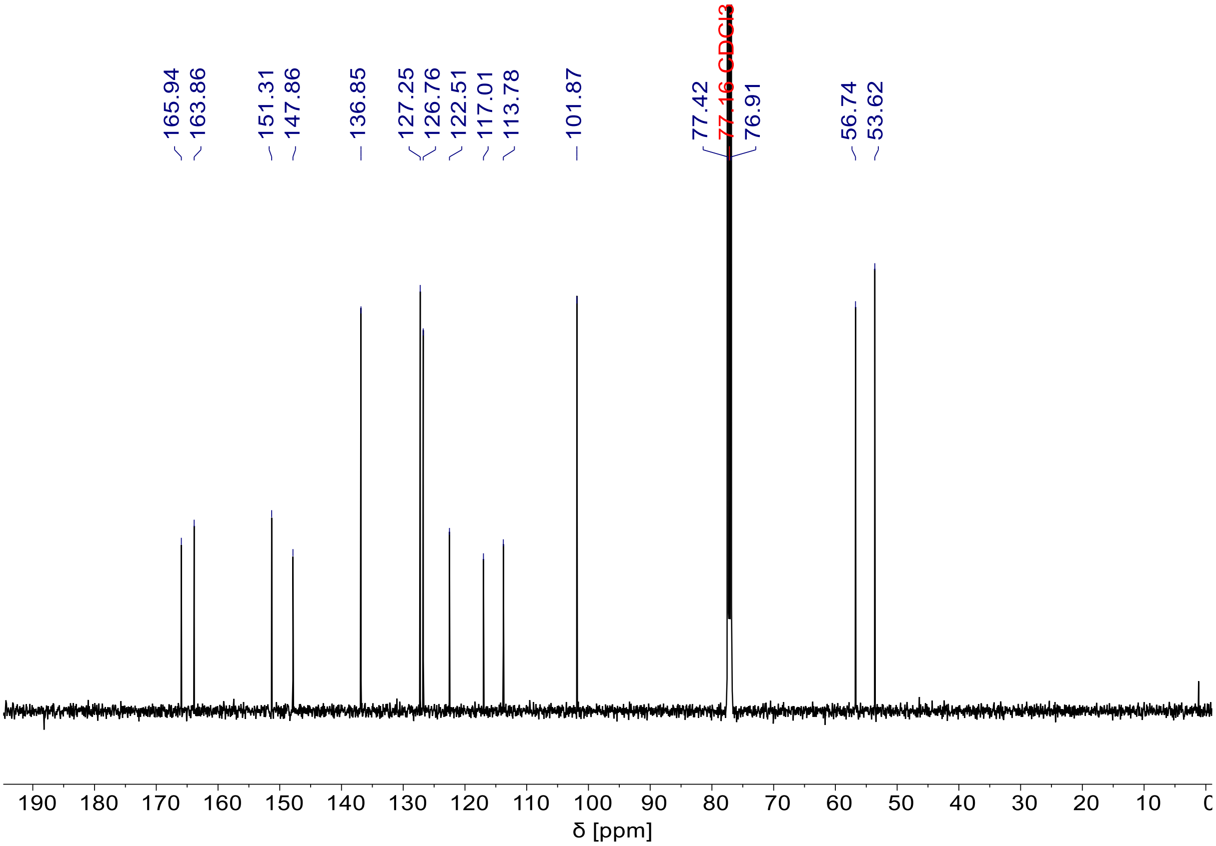

Figure S 25 ^{13}C NMR spectrum of compound **13** in $CDCl_3$.

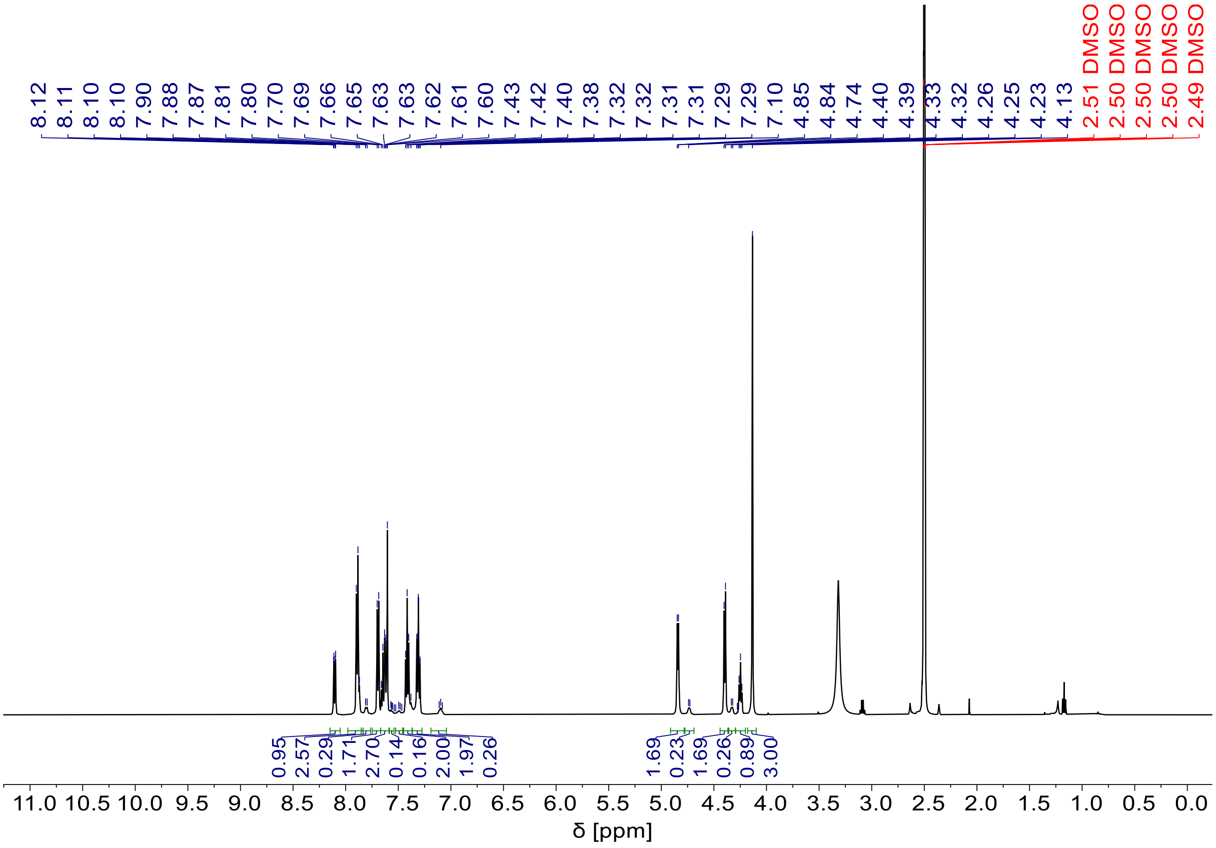

Figure S 26 ^{1}H NMR spectrum of compound **14** in DMSO-d_6.

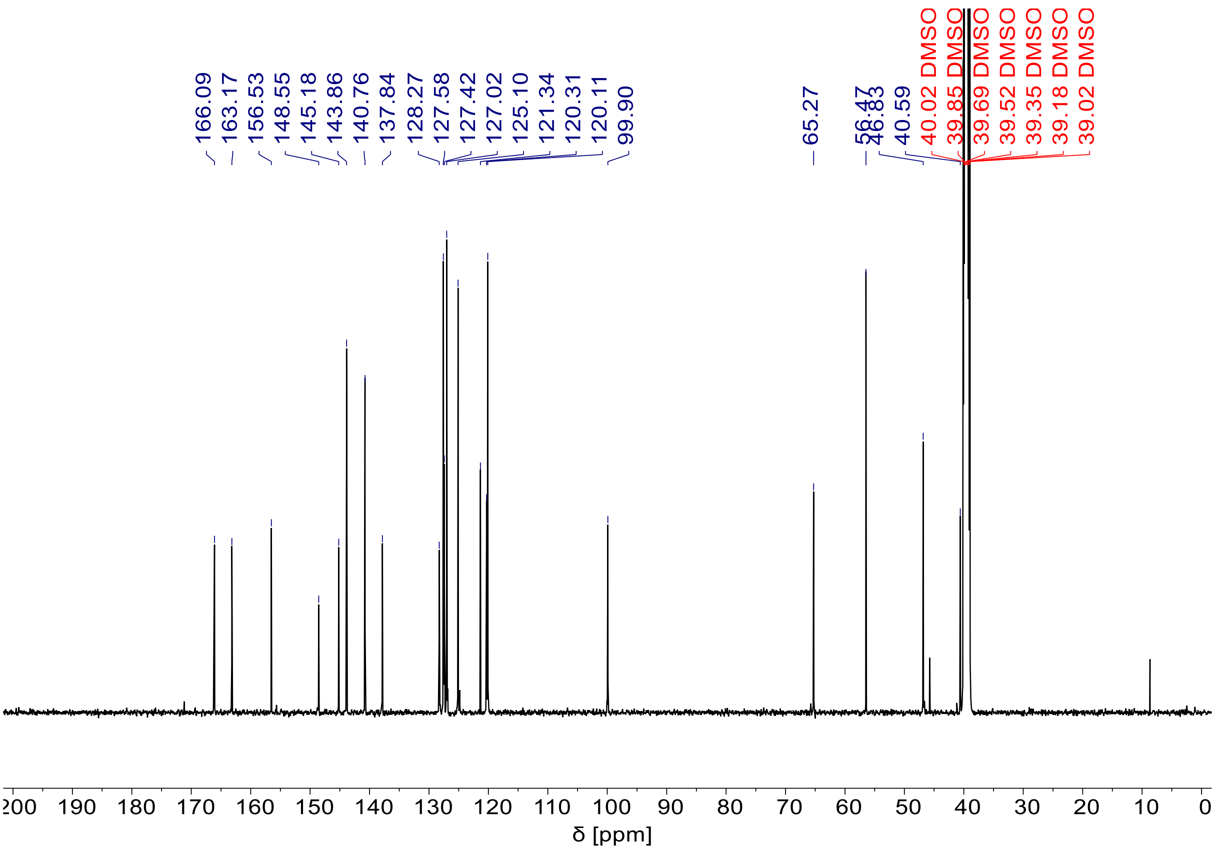

Figure S 27 ^{1}H NMR spectrum of compound **14** in DMSO-d_6.

3.1.2 Oligomer NMR spectra

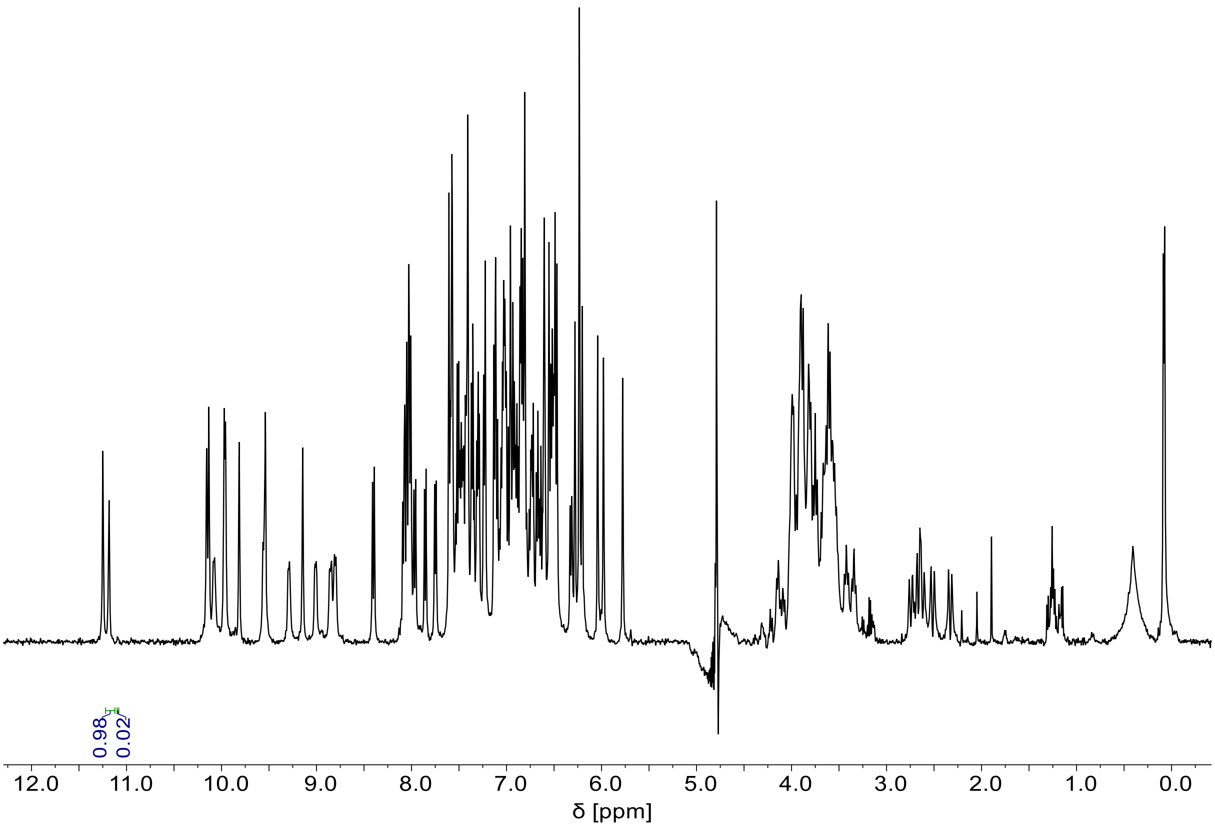

Figure S 28 ^{1}H NMR of spectrum compound **5** in 50 mM NH_4HCO_3 (H_2O/D_2O, 9:1, v/v) at 25 °C.

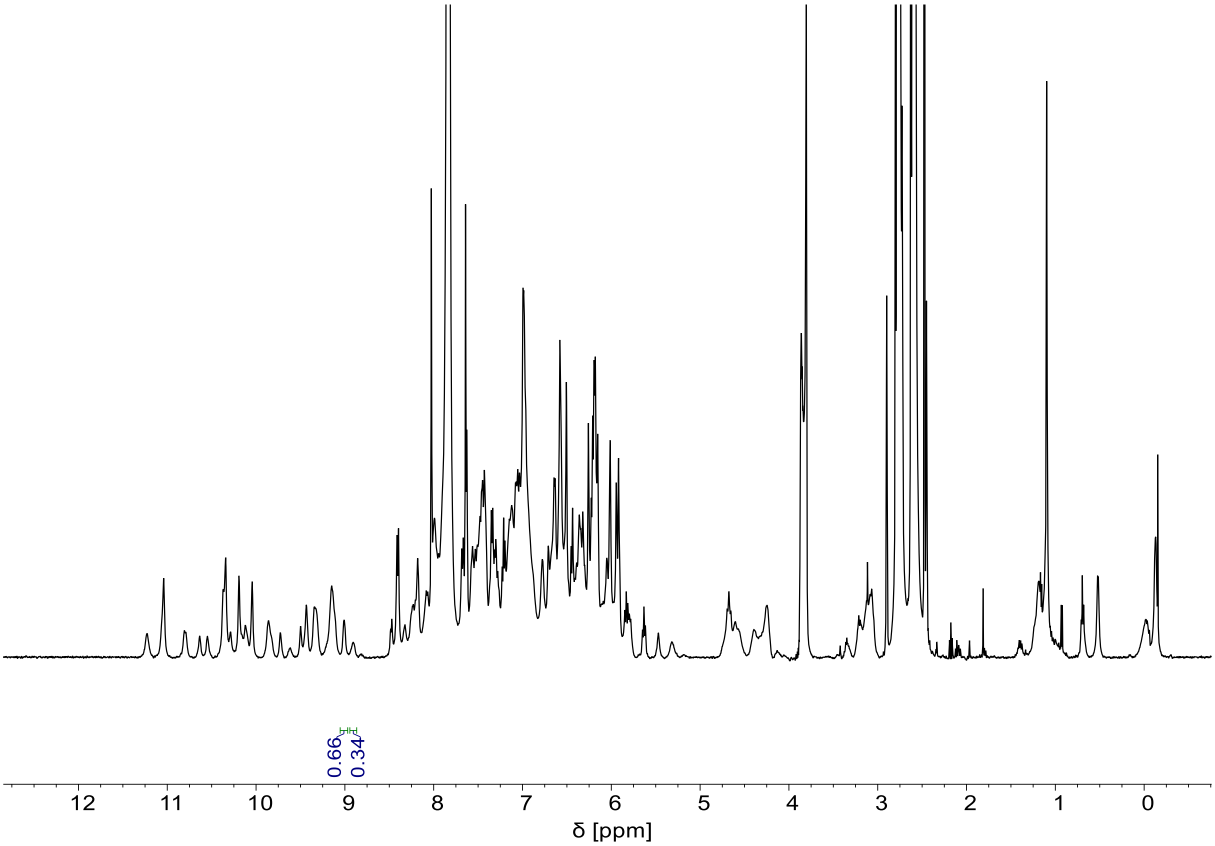

Figure S 29 ^{1}H NMR spectrum of compound **5** in (DMF-d_7/H_2O, 9:1, v/v) at 25 °C.

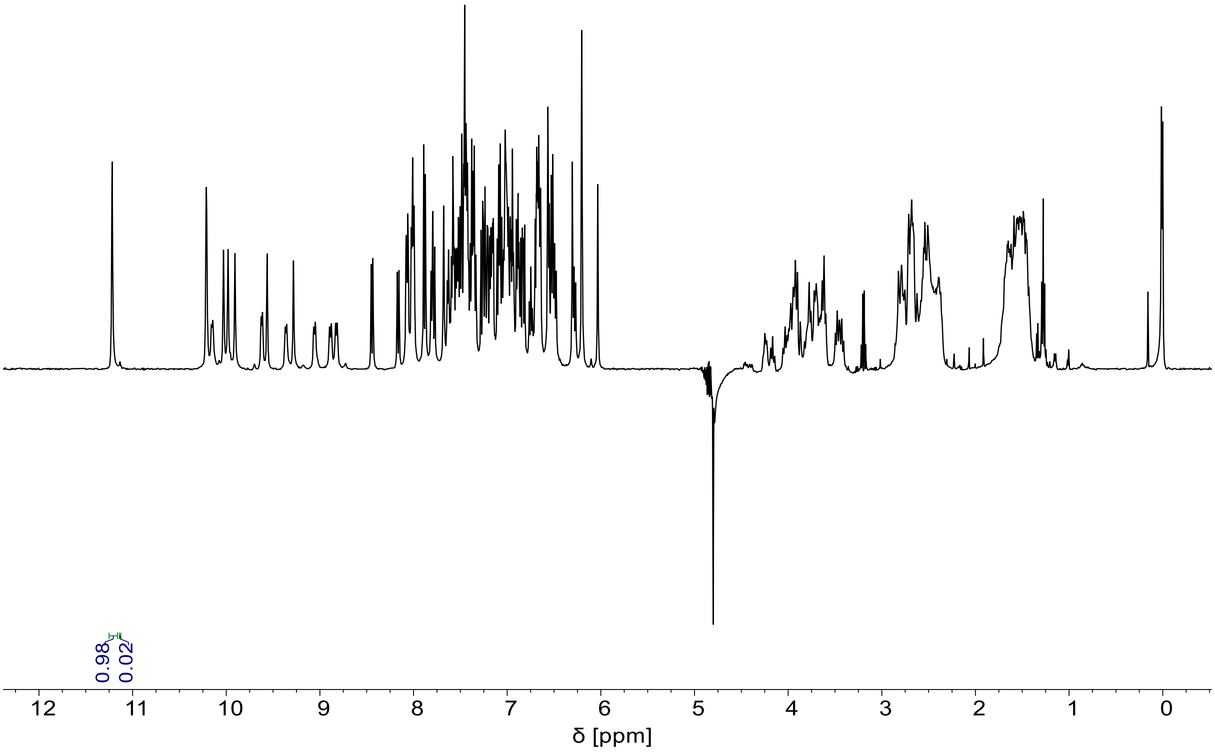

Figure S 30 ^{1}H NMR spectrum of compound **6** in 50 mM NH_4HCO_3 (H_2O/D_2O, 9:1, v/v) at 25 °C.

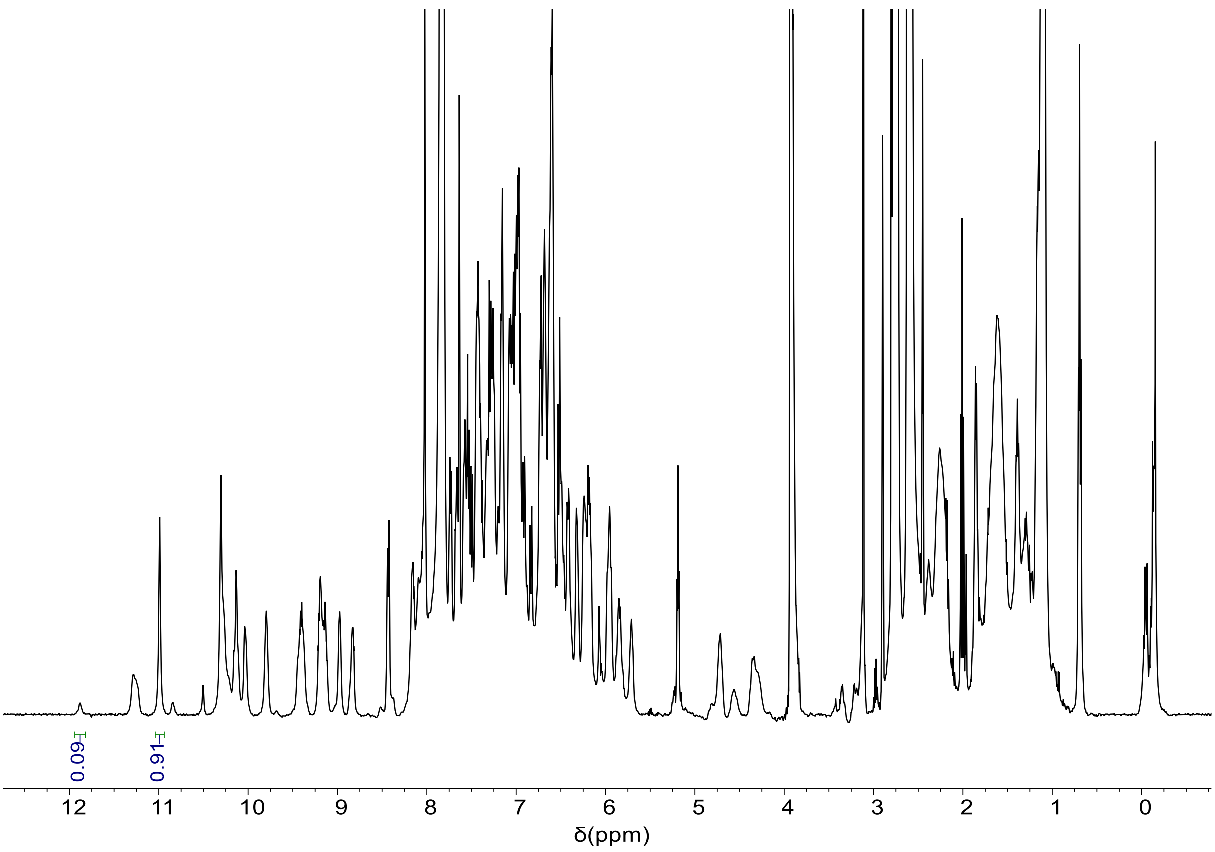

Figure S 31 ^{1}H NMR spectrum of compound **6** in (DMF-d_7/H_2O, 9:1, v/v) at 25 °C.

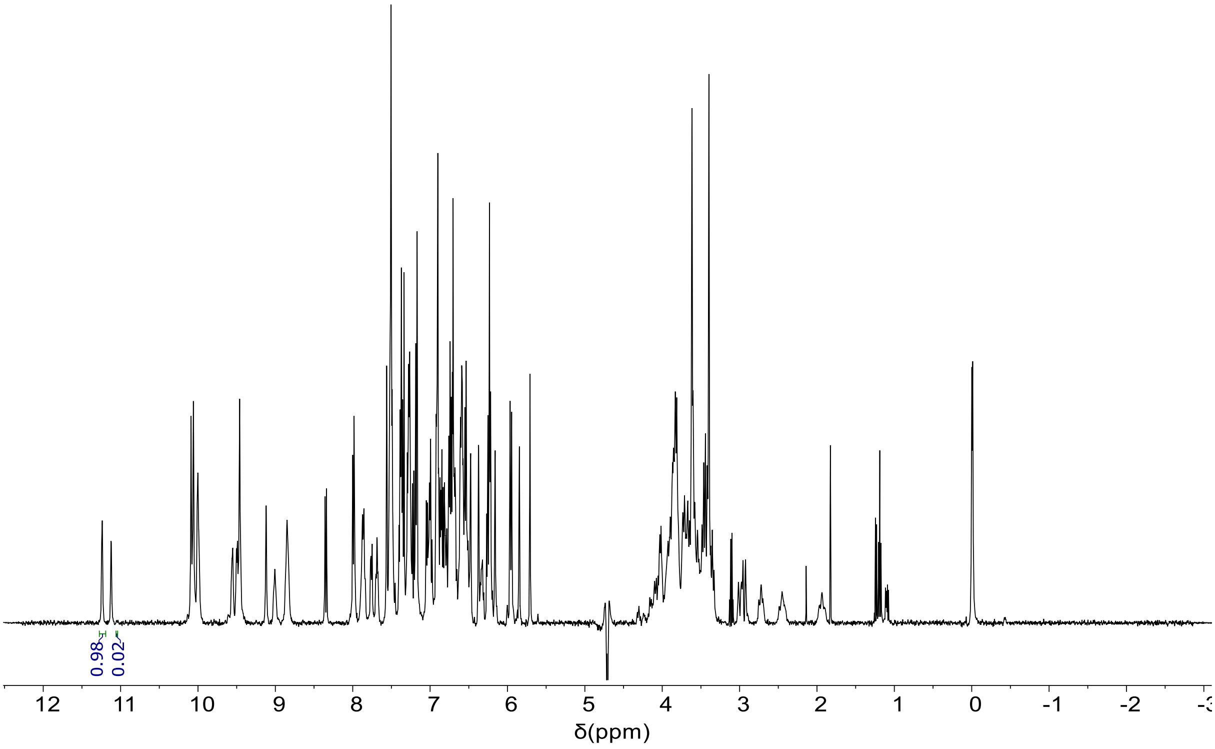

Figure S 32 ^{1}H NMR spectrum of compound **7** in 50 mM NH_4HCO_3 (H_2O/D_2O, 9:1, v/v) at 25 °C.

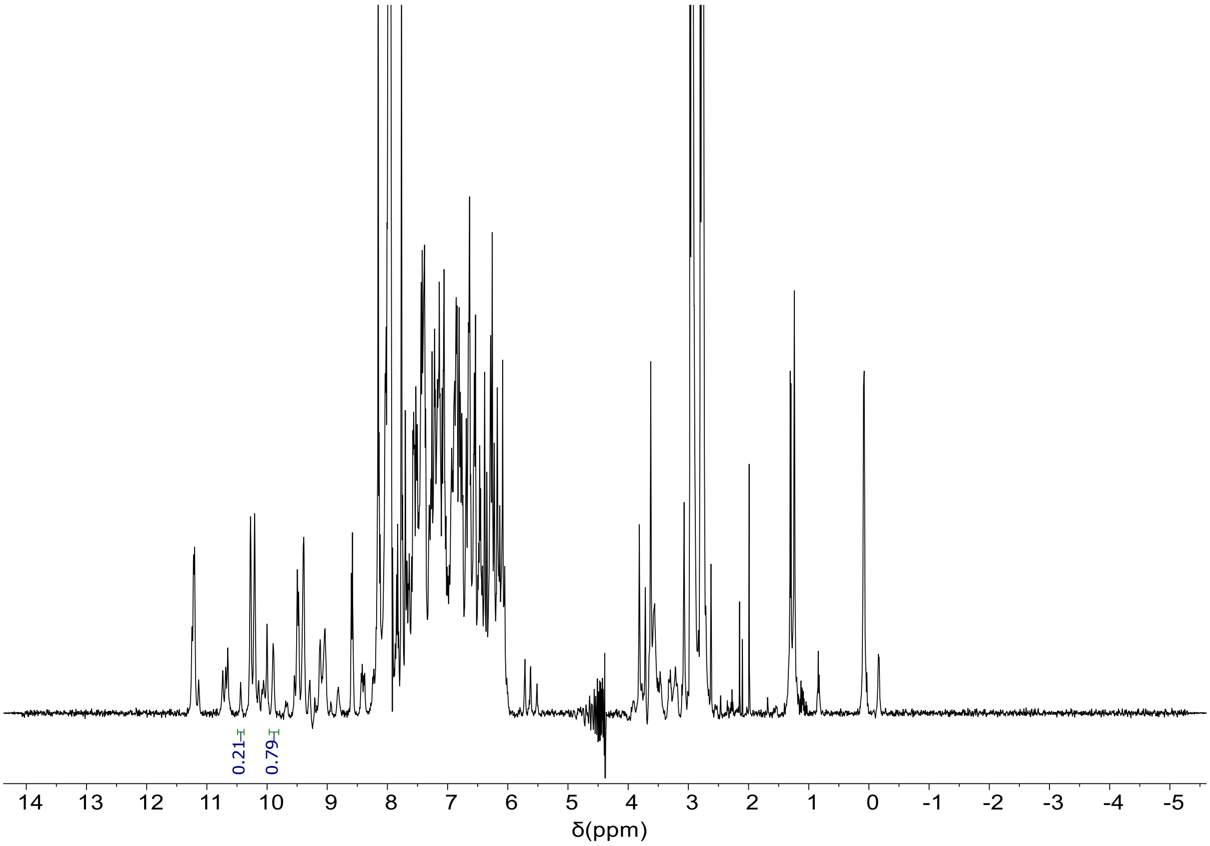

Figure S 33 ^{1}H NMR spectrum of compound **7** in (DMF-d_7/H_2O, 9:1, v/v) at 25 °C.

3.2 HPLC chromatograms

Compound **11**

Gradient: 50-100 % B in A, 0.1% TFA buffer

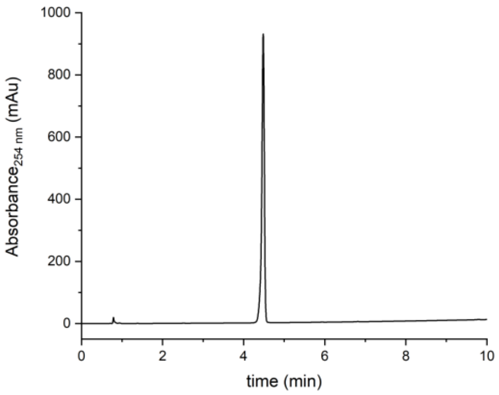

Compound **14**

Gradient: 50-100 % B in A, 0.1% TFA buffer

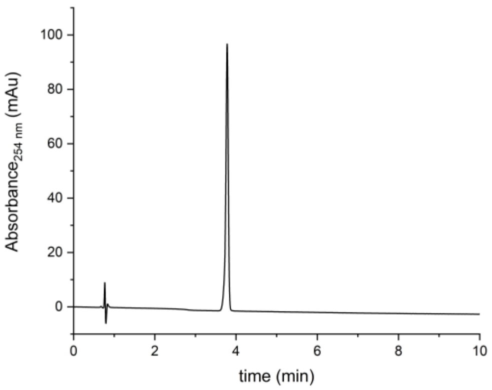

Compound **5a**

Gradient: 30-70 % B in A, 0.1% TFA buffer

Column: Nucleodur C18

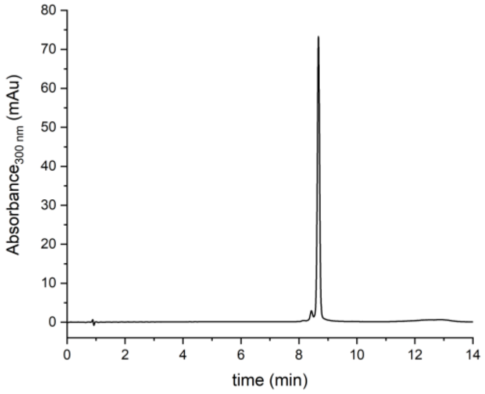

Compound **5**

Gradient: 0-100 % B in A, TEAA buffer

Column: Nucleodur C18

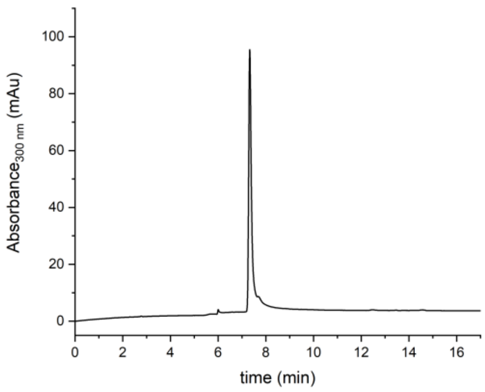

Compound **6a**

Gradient: 30-100 % B in A, 0.1% TFA buffer

Column: Nucleodur C18

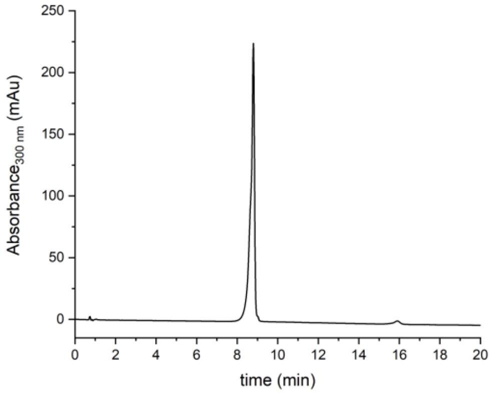

Compound **6**

Gradient: 0-100 % B in A, TEAA buffer

Column: Nucleodur C18

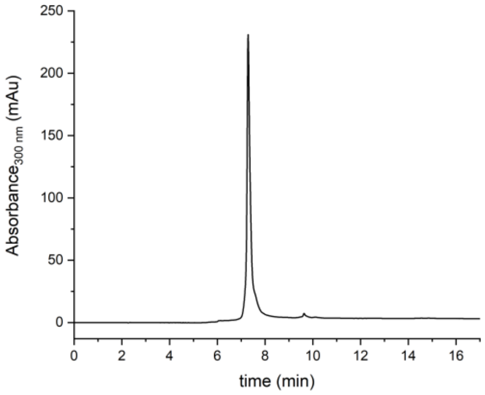

Compound **7a**

Gradient: 30-100 % B in A, 0.1% TFA buffer

Column: Nucleodur C18

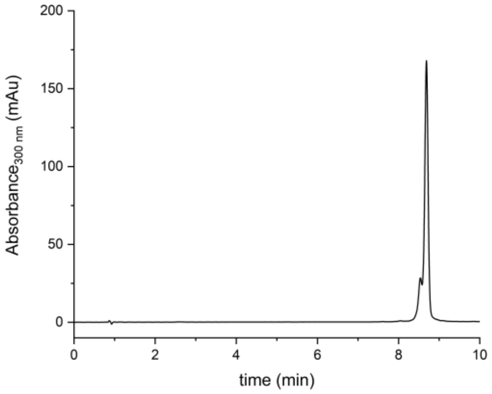

Compound **7**

Gradient: 0-100 % B in A, TEAA buffer

Column: Nucleodur C18

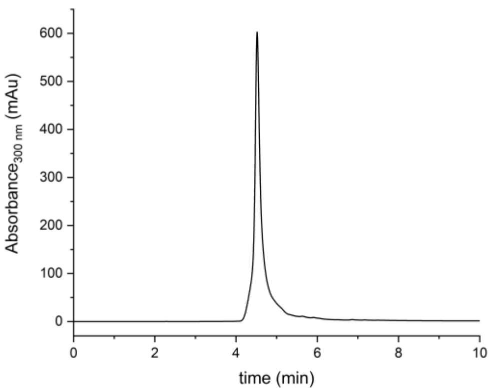

3.3 Mass spectra

3.3.1 Foldamer mass spectra

Compound **5a**

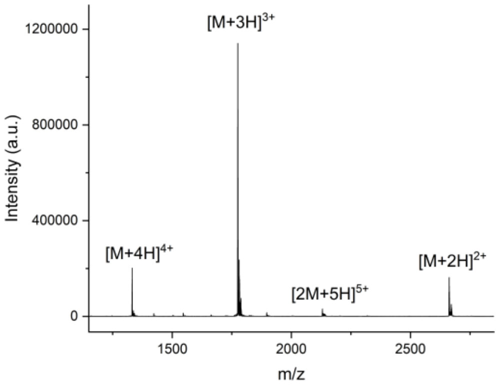

Compound **5a** Zoom

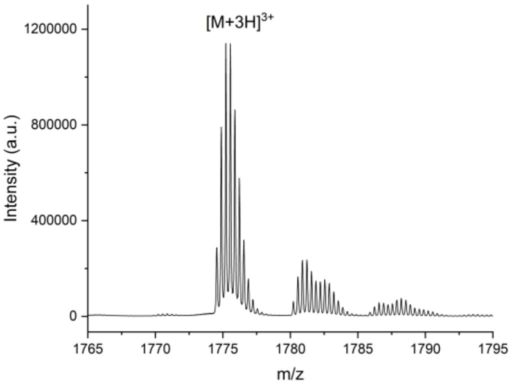

Compound **5**

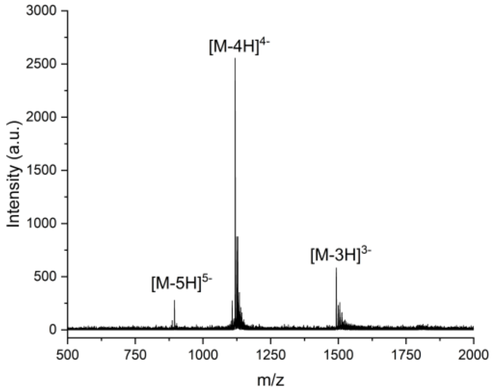

Compound **5** Zoom

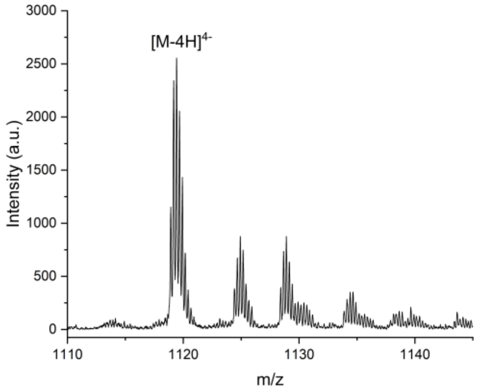

Compound **6a**

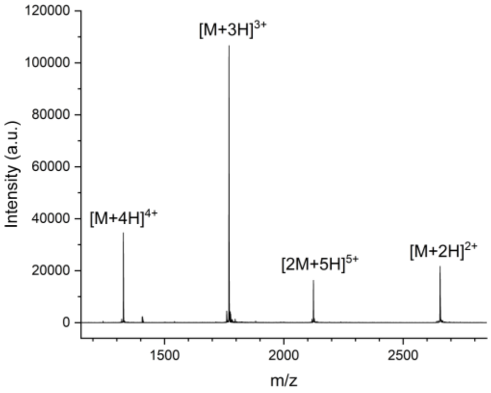

Compound **6a** Zoom

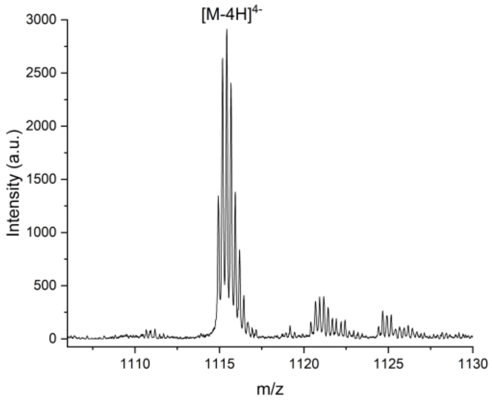

Compound **6**

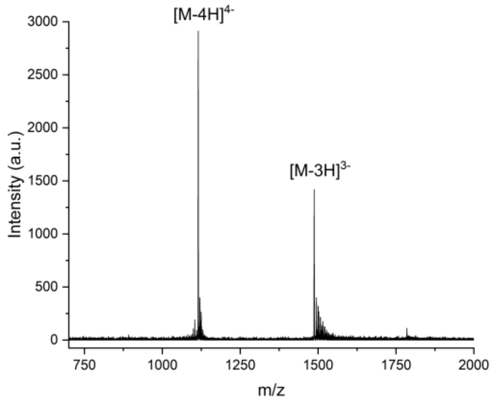

Compound **6** Zoom

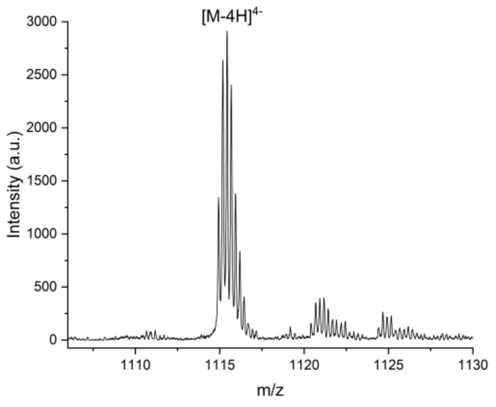

Compound **7a**

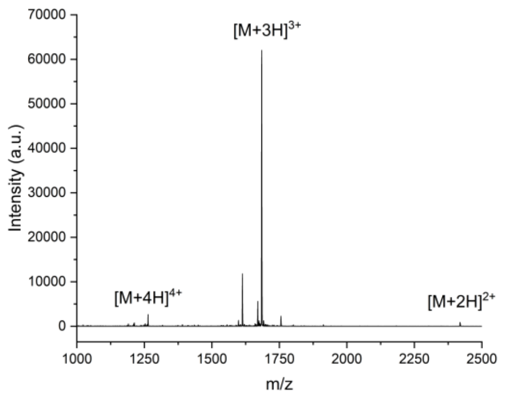

Compound **7a** Zoom

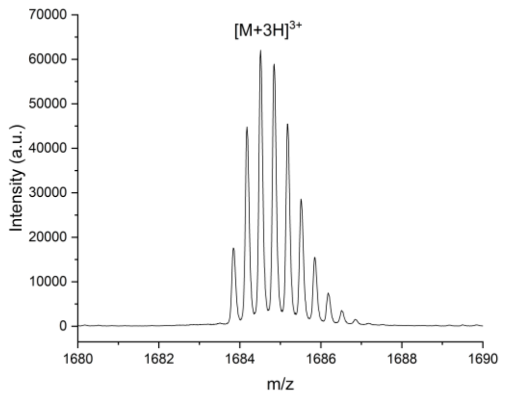

Compound **7**

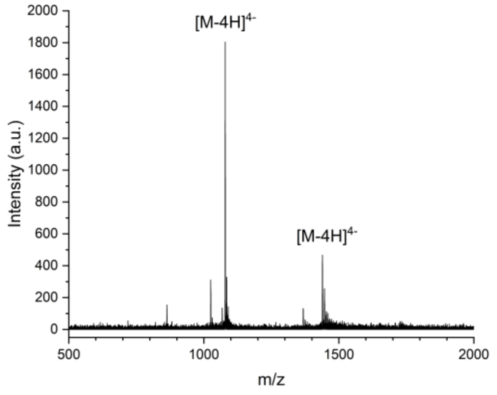

Compound **7** Zoom

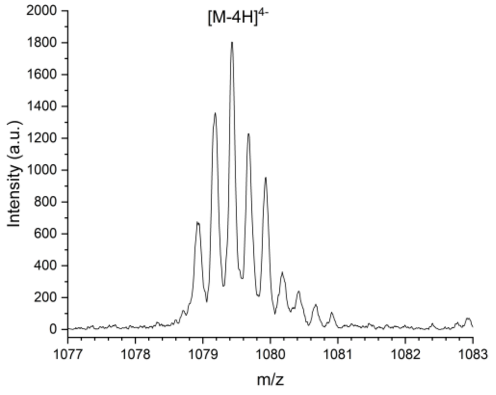

4 References

1. G. R. Fulmer, A. J. M. Miller, N. H. Sherden, H. E. Gottlieb, A. Nudelman, B. M. Stoltz, J. E. Bercaw and K. I. Goldberg, *Organometallics*, 2010, **29**, 2176-2179.
2. D. Carbajo, P. Prabhakaran, K. Ziach, P. K. Mandal, V. Dos Santos, C. Legeay, R. Vogel, V. Parissi, P. Pourquier and I. Huc, *Nucleic Acids Res.*, 2019, **47**, 5511-5521
3. V. Corvaglia, J. Wu, D. Deepak, M. Loos and I. Huc, *Chem. - Eur. J.*, 2024, **30**, e202303650.
4. D. Bindl, E. Heinemann, P. K. Mandal and I. Huc, *Chem. Commun.*, 2021, **57**, 5662-5665.
5. X. Hu, S. J. Dawson, P. K. Mandal, X. de Hatten, B. Baptiste and I. Huc, *Chem. Sci.*, 2017, **8**, 3741-3749.
6. K. Ziach, C. Chollet, V. Parissi, P. Prabhakaran, M. Marchivie, V. Corvaglia, P. P. Bose, K. Laxmi-Reddy, F. Godde, J.-M. Schmitter, S. Chaignepain, P. Pourquier and I. Huc, *Nat. Chem.*, 2018, **10**, 511-518.
7. V. Corvaglia, F. Sanchez, F. S. Menke, C. Douat and I. Huc, *Chem. - Eur. J.*, 2023, **29**, e202300898.
